# Higgs physics at the CLIC electron–positron linear collider

**DOI:** 10.1140/epjc/s10052-017-4968-5

**Published:** 2017-07-17

**Authors:** H. Abramowicz, A. Abusleme, K. Afanaciev, N. Alipour Tehrani, C. Balázs, Y. Benhammou, M. Benoit, B. Bilki, J.-J. Blaising, M. J. Boland, M. Boronat, O. Borysov, I. Božović-Jelisavčić, M. Buckland, S. Bugiel, P. N. Burrows, T. K. Charles, W. Daniluk, D. Dannheim, R. Dasgupta, M. Demarteau, M. A. Díaz Gutierrez, G. Eigen, K. Elsener, U. Felzmann, M. Firlej, E. Firu, T. Fiutowski, J. Fuster, M. Gabriel, F. Gaede, I. García, V. Ghenescu, J. Goldstein, S. Green, C. Grefe, M. Hauschild, C. Hawkes, D. Hynds, M. Idzik, G. Kačarević, J. Kalinowski, S. Kananov, W. Klempt, M. Kopec, M. Krawczyk, B. Krupa, M. Kucharczyk, S. Kulis, T. Laštovička, T. Lesiak, A. Levy, I. Levy, L. Linssen, S. Lukić, A. A. Maier, V. Makarenko, J. S. Marshall, V. J. Martin, K. Mei, G. Milutinović-Dumbelović, J. Moroń, A. Moszczyński, D. Moya, R. M. Münker, A. Münnich, A. T. Neagu, N. Nikiforou, K. Nikolopoulos, A. Nürnberg, M. Pandurović, B. Pawlik, E. Perez Codina, I. Peric, M. Petric, F. Pitters, S. G. Poss, T. Preda, D. Protopopescu, R. Rassool, S. Redford, J. Repond, A. Robson, P. Roloff, E. Ros, O. Rosenblat, A. Ruiz-Jimeno, A. Sailer, D. Schlatter, D. Schulte, N. Shumeiko, E. Sicking, F. Simon, R. Simoniello, P. Sopicki, S. Stapnes, R. Ström, J. Strube, K. P. Świentek, M. Szalay, M. Tesař, M. A. Thomson, J. Trenado, U. I. Uggerhøj, N. van der Kolk, E. van der Kraaij, M. Vicente Barreto Pinto, I. Vila, M. Vogel Gonzalez, M. Vos, J. Vossebeld, M. Watson, N. Watson, M. A. Weber, H. Weerts, J. D. Wells, L. Weuste, A. Winter, T. Wojtoń, L. Xia, B. Xu, A. F. Żarnecki, L. Zawiejski, I.-S. Zgura

**Affiliations:** 1 Raymond and Beverly Sackler School of Physics and Astronomy, Tel Aviv University, Tel Aviv, Israel; 2 Pontificia Universidad Católica de Chile, Santiago, Chile; 3 National Scientific and Educational Centre of Particle and High Energy Physics, Belarusian State University, Minsk, Belarus; 4 CERN, Geneva, Switzerland; 5 Monash University, Melbourne, Australia; 6 Département de Physique Nucléaire et Corpusculaire (DPNC), Université de Genève, Geneva, Switzerland; 7 Argonne National Laboratory, Argonne, IL USA; 8 Laboratoire d’Annecy-le-Vieux de Physique des Particules, Annecy-le-Vieux, France; 9 University of Melbourne, Melbourne, Australia; 10 IFIC, CSIC-University of Valencia, Valencia, Spain; 11 Vinča Institute of Nuclear Sciences, University of Belgrade, Belgrade, Serbia; 12 University of Liverpool, Liverpool, UK; 13 Faculty of Physics and Applied Computer Science, AGH University of Science and Technology, Crakow, Poland; 14 Oxford University, Oxford, UK; 15 The Henryk Niewodniczański Institute of Nuclear Physics Polish Academy of Sciences, Crakow, Poland; 16 Department of Physics and Technology, University of Bergen, Bergen, Norway; 17 Institute of Space Science, Bucharest, Romania; 18 Max-Planck-Institut für Physik, Munich, Germany; 19 DESY, Hamburg, Germany; 20 University of Bristol, Bristol, UK; 21 Cavendish Laboratory, University of Cambridge, Cambridge, UK; 22 University of Edinburgh, Edinburgh, UK; 23 School of Physics and Astronomy, University of Birmingham, Birmingham, UK; 24 Faculty of Physics, University of Warsaw, Warsaw, Poland; 25 Institute of Physics of the Academy of Sciences of the Czech Republic, Prague, Czech Republic; 26 IFCA, CSIC-University of Cantabria, Santander, Spain; 27 Karlsruher Institut für Technologie (KIT), Institut für Prozessdatenverarbeitung und Elektronik (IPE), Karlsruhe, Germany; 28 University of Glasgow, Glasgow, UK; 29 University of Barcelona, Barcelona, Spain; 30 Aarhus University, Aarhus, Denmark; 31 Physics Department, University of Michigan, Ann Arbor, MI USA

## Abstract

The Compact Linear Collider (CLIC) is an option for a future ${\mathrm{e}^{+}}{\mathrm{e}^{-}}$ collider operating at centre-of-mass energies up to $3\,\text {TeV}$, providing sensitivity to a wide range of new physics phenomena and precision physics measurements at the energy frontier. This paper is the first comprehensive presentation of the Higgs physics reach of CLIC operating at three energy stages: $\sqrt{s} = 350\,\text {GeV}$, 1.4 and $3\,\text {TeV}$. The initial stage of operation allows the study of Higgs boson production in Higgsstrahlung (${\mathrm{e}^{+}}{\mathrm{e}^{-}} \rightarrow {\mathrm{Z}} {\mathrm{H}}$) and ${\mathrm{W}} {\mathrm{W}}$-fusion (${\mathrm{e}^{+}}{\mathrm{e}^{-}} \rightarrow {\mathrm{H}} {{\nu }}_{\!\mathrm{e}} {\bar{{\nu }}}_{\!\mathrm{e}}$), resulting in precise measurements of the production cross sections, the Higgs total decay width $\varGamma _{{\mathrm{H}}}$, and model-independent determinations of the Higgs couplings. Operation at $\sqrt{s} > 1\,\text {TeV}$ provides high-statistics samples of Higgs bosons produced through ${\mathrm{W}} {\mathrm{W}}$-fusion, enabling tight constraints on the Higgs boson couplings. Studies of the rarer processes ${\mathrm{e}^{+}}{\mathrm{e}^{-}} \rightarrow \mathrm{t} {\bar{\mathrm{t}}} {\mathrm{H}}$ and ${\mathrm{e}^{+}}{\mathrm{e}^{-}} \rightarrow {\mathrm{H}} {\mathrm{H}} {{\nu }}_{\!\mathrm{e}} {\bar{{\nu }}}_{\!\mathrm{e}}$ allow measurements of the top Yukawa coupling and the Higgs boson self-coupling. This paper presents detailed studies of the precision achievable with Higgs measurements at CLIC and describes the interpretation of these measurements in a global fit.

## Introduction

The discovery of a Higgs boson [1, 2] at the Large Hadron Collider (LHC) provided confirmation of the electroweak symmetry breaking mechanism [3–8] of the Standard Model (SM). However, it is not yet known if the observed Higgs boson is the fundamental scalar of the SM or is either a more complex object or part of an extended Higgs sector. Precise studies of the properties of the Higgs boson at the LHC and future colliders are essential to understand its true nature.

The Compact Linear Collider (CLIC) is a mature option for a future multi-$\text {TeV}$ high-luminosity linear ${\mathrm{e}^{+}}{\mathrm{e}^{-}}$ collider that is currently under development at CERN. It is based on a novel two-beam acceleration technique providing accelerating gradients of 100 MV/m. Recent implementation studies for CLIC have converged towards a staged approach. In this scheme, CLIC provides high-luminosity ${\mathrm{e}^{+}}{\mathrm{e}^{-}}$ collisions at centre-of-mass energies from a few 100 $\text {GeV}$ up to 3 TeV. The ability of CLIC to collide ${\mathrm{e}^{+}}{\mathrm{e}^{-}}$ up to multi-TeV energy scales is unique. For the current study, the nominal centre-of-mass energy of the first energy stage is $\sqrt{s} =350\,\text {GeV}$. At this centre-of-mass energy, the Higgsstrahlung and ${\mathrm{W}} {\mathrm{W}}$-fusion processes have significant cross sections, providing access to precise measurement of the absolute values of the Higgs boson couplings to both fermions and bosons. Another advantage of operating CLIC at $\sqrt{s} \approx 350\,\text {GeV}$ is that it enables a programme of precision top quark physics, including a scan of the $\mathrm{t} {\bar{\mathrm{t}}}$ cross section close to the production threshold. In practice, the centre-of-mass energy of the second stage of CLIC operation will be motivated by both the machine design and results from the LHC. In this paper, it is assumed that the second CLIC energy stage has $\sqrt{s} =1.4\,\text {TeV}$ and that the ultimate CLIC centre-of-mass energy is $3\,\text {TeV}$. In addition to direct and indirect searches for Beyond the Standard Model (BSM) phenomena, these higher energy stages of operation provide a rich potential for Higgs physics beyond that accessible at lower energies, such as the direct measurement of the top Yukawa coupling and a direct probe of the Higgs potential through the measurement of the Higgs self-coupling. Furthermore, rare Higgs boson decays become accessible due to the higher integrated luminosities at higher energies and the increasing cross section for Higgs production in ${\mathrm{W}} {\mathrm{W}}$-fusion. The proposed staged approach spans around twenty years of running.

The following sections describe the experimental conditions at CLIC, an overview of Higgs production at CLIC, and the Monte Carlo samples, detector simulation, and event reconstruction used for the subsequent studies. Thereafter, Higgs production at $\sqrt{s} = 350\,\text {GeV}$, Higgs production in ${\mathrm{W}} {\mathrm{W}}$-fusion at $\sqrt{s} > 1\,\text {TeV}$, Higgs production in ${\mathrm{Z}} {\mathrm{Z}}$-fusion, the measurement of the top Yukawa coupling, double Higgs production, and measurements of the Higgs boson mass are presented. The paper concludes with a discussion of the measurement precisions on the Higgs couplings obtained in a combined fit to the expected CLIC results, and the systematic uncertainties associated with the measurements.

The detailed study of the CLIC potential for Higgs physics presented here supersedes earlier preliminary estimates [9]. The work is carried out by the CLIC Detector and Physics (CLICdp) collaboration.

## Experimental environment at CLIC

The experimental environment at CLIC is characterised by challenging conditions imposed by the CLIC accelerator technology, by detector concepts optimised for the precise reconstruction of complex final states in the multi-TeV energy range, and by the operation in several energy stages to maximise the physics potential.

### Accelerator and beam conditions

The CLIC accelerator design is based on a two-beam acceleration scheme. It uses a high-intensity drive beam to efficiently generate radio frequency (RF) power at 12 GHz. The RF power is used to accelerate the main particle beam that runs parallel to the drive beam. CLIC uses normal-conducting accelerator structures, operated at room temperature. These structures permit high accelerating gradients, while the short pulse duration discussed below limits ohmic losses to tolerable levels. The initial drive beams and the main electron/positron beams are generated in the central complex and are then injected at the ends of the two linac arms. The feasibility of the CLIC accelerator has been demonstrated through prototyping, simulations and large-scale tests, as described in the Conceptual Design Report [10]. In particular, the two-beam acceleration at gradients exceeding 100 MV/m has been demonstrated in the CLIC test facility, CTF3. High luminosities are achievable by very small beam emittances, which are generated in the injector complex and maintained during transport to the interaction point.

**Fig. 1:**
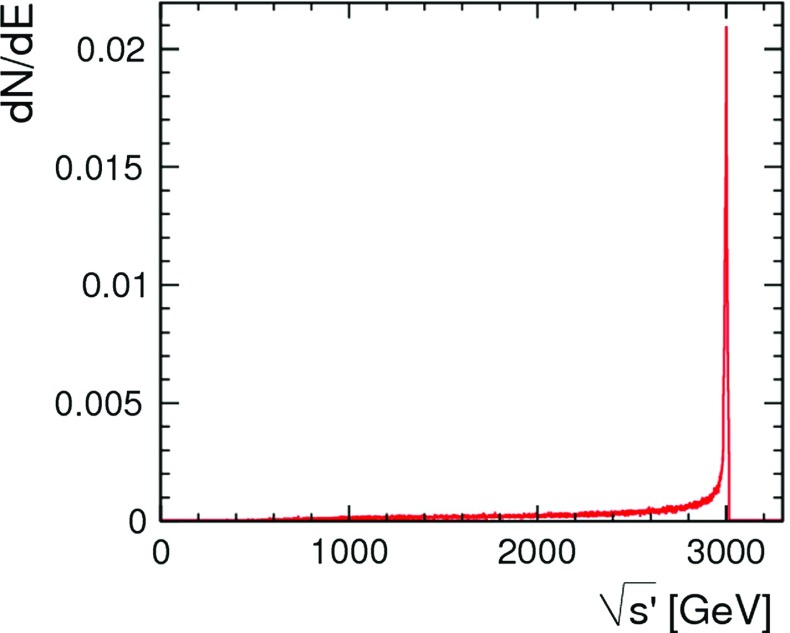
The luminosity spectrum for CLIC operating at $\sqrt{s} = 3\,\text {TeV}$, where $\sqrt{s^\prime }$ is the effective centre-of-mass energy after beamstrahlung and initial state radiation [11]

CLIC will be operated with a bunch train repetition rate of 50 Hz. Each bunch train consists of 312 individual bunches, with 0.5 ns between bunch crossings at the interaction point. The average number of hard ${\mathrm{e}^{+}}{\mathrm{e}^{-}}$ interactions in a single bunch train crossing is much less than one. However, for CLIC operation at $\sqrt{s} > 1\,\text {TeV}$, the highly-focussed intense beams lead to significant beamstrahlung (radiation of photons from electrons/positrons in the electric field of the other beam). Beamstrahlung results in high rates of incoherent electron–positron pairs and low-$Q^2$ *t*-channel multi-peripheral ${\upgamma } {\upgamma } \rightarrow \text {hadron}$ events, where $Q^2$ is the negative of the four-momentum squared of the virtual space-like photon. In addition, the energy loss through beamstrahlung generates a long lower-energy tail to the luminosity spectrum that extends well below the nominal centre-of-mass energy, as shown in Fig. 1. Both the CLIC detector design and the event reconstruction techniques employed are optimised to mitigate the influence of these backgrounds, which are most severe at the higher CLIC energies; this is discussed further in Sect. 4.2.

The baseline machine design allows for up to ±80% longitudinal electron spin-polarisation by using GaAs-type cathodes [10]; and provisions have been made to allow positron polarisation as an upgrade option. Most studies presented in this paper are performed for zero beam polarisation and are subsequently scaled to account for the increased cross sections with left-handed polarisation for the electron beam.

### Detectors at CLIC

The detector concepts used for the CLIC physics studies, described here and elsewhere [11], are based on the SiD [12, 13] and ILD [13, 14] detector concepts for the International Linear Collider (ILC). They were initially adapted for the CLIC $3\,\text {TeV}$ operation, which constitutes the most challenging environment for the detectors in view of the high beam-induced background levels. For most sub-detector systems, the $3\,\text {TeV}$ detector design is suitable at all energy stages, the only exception being the inner tracking detectors and the vertex detector, where the lower backgrounds at $\sqrt{s} =350\,\text {GeV}$ enable detectors to be deployed with a smaller inner radius.

**Fig. 2:**
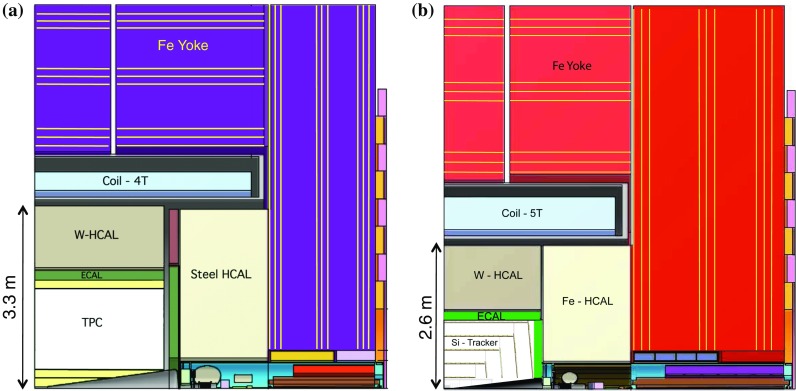
Longitudinal cross section of the top right quadrant of the CLIC_ILD (**a**) and CLIC_SiD (**b**) detector concepts

The key performance parameters of the CLIC detector concepts with respect to the Higgs programme are:

- excellent track-momentum resolution of $\sigma _{p_\mathrm{T}}/p_\mathrm{T}^2 \lesssim 2 \cdot 10^{-5}$ $\text {GeV} ^{-1}$, required for a precise reconstruction of leptonic ${\mathrm{Z}}$ decays in ${\mathrm{Z}} {\mathrm{H}}$ events;
- precise impact parameter resolution, defined by $a \lesssim 5\,\upmu \text {m}$ and $b \lesssim 15\,\upmu \text {m} \,\text {GeV}$ in $\sigma _{d_0}^2 = a^2 + b^2/(p^2\sin ^3\theta )$ to provide accurate vertex reconstruction, enabling flavour tagging with clean ${\mathrm{b}}$-, ${\mathrm{c}}$- and light-quark jet separation;
- jet-energy resolution $\sigma _E/E \lesssim 3.5\%$ for light-quark jet energies in the range 100 $\text {GeV}$ to 1 $\text {TeV}$, required for the reconstruction of hadronic ${\mathrm{Z}}$ decays in ${\mathrm{Z}} {\mathrm{H}}$ events and the separation of ${\mathrm{Z}} \rightarrow \mathrm{q}{\bar{\mathrm{q}}}$ and ${\mathrm{H}} \rightarrow \mathrm{q}{\bar{\mathrm{q}}}$ based on the reconstructed di-jet invariant mass;
- detector coverage for electrons extending to very low angles with respect to the beam axis, to maximise background rejection for ${\mathrm{W}} {\mathrm{W}}$-fusion events.

The main design driver for the CLIC (and ILC) detector concepts is the required jet-energy resolution. As a result, the CLIC detector concepts [11], CLIC_SiD and CLIC_ILD, are based on fine-grained electromagnetic and hadronic calorimeters (ECAL and HCAL), optimised for particle-flow reconstruction techniques. In the particle-flow approach, the aim is to reconstruct the individual final-state particles within a jet using information from the tracking detectors combined with that from the highly granular calorimeters [15–18]. In addition, particle-flow event reconstruction provides a powerful tool for the rejection of beam-induced backgrounds [11]. The CLIC detector concepts employ strong central solenoid magnets, located outside the HCAL, providing an axial magnetic field of 5 T in CLIC_SiD and 4 T in CLIC_ILD. The CLIC_SiD concept employs central silicon-strip tracking detectors, whereas CLIC_ILD assumes a large central gaseous Time Projection Chamber. In both concepts, the central tracking system is augmented with silicon-based inner tracking detectors. The two detector concepts are shown schematically in Fig. 2 and are described in detail in [11].

### Assumed staged running scenario

The studies presented in this paper are based on a scenario in which CLIC runs at three energy stages. The first stage is at $\sqrt{s} = 350\,\text {GeV}$, around the top-pair production threshold. The second stage is at $\sqrt{s} = 1.4\,\text {TeV}$; this energy is chosen because it can be reached with a single CLIC drive-beam complex. The third stage is at $\sqrt{s} = 3\,\text {TeV}$; the ultimate energy of CLIC. At each stage, four to five years of running with a fully commissioned accelerator is foreseen, providing integrated luminosities of $500\,\text {fb}^{-1}$, 1.5 and $2\,\text {ab}^{-1}$ at $350\,\text {GeV}$, 1.4 and $3\,\text {TeV}$, respectively.^1^ Cross sections and integrated luminosities for the three stages are summarised in Table 1.

**Table 1:** Leading-order, unpolarised cross sections for Higgsstrahlung, ${\mathrm{W}} {\mathrm{W}}$-fusion, and ${\mathrm{Z}} {\mathrm{Z}}$-fusion processes for $m_{{\mathrm{H}}} =126\,\text {GeV}$ at the three centre-of-mass energies discussed in this paper. $\sqrt{s^\prime }$ is the effective centre-of-mass energy of the ${\mathrm{e}^{+}}{\mathrm{e}^{-}}$ collision. The presented cross sections include the effects of ISR but exclude the effects of beamstrahlung. Also given are numbers of expected events, including the effects of ISR and the CLIC beamstrahlung spectrum. The presented cross sections and event numbers do not include possible enhancements from polarised beams

$\sqrt{s} =$	350 GeV	1.4 TeV	3 TeV
$\int \frac{d \mathscr {L}}{d s'} ds'$	500 $\text {fb}^{-1}$	1.5 $\text {ab}^{-1}$	2 $\text {ab}^{-1}$
$\sigma ({\mathrm{e}^{+}}{\mathrm{e}^{-}} \rightarrow {\mathrm{Z}} {\mathrm{H}})$	133 fb	8 fb	2 fb
$\sigma ({\mathrm{e}^{+}}{\mathrm{e}^{-}} \rightarrow {\mathrm{H}} {{\nu }}_{\!\mathrm{e}} {\bar{{\nu }}}_{\!\mathrm{e}})$	34 fb	276 fb	477 fb
$\sigma ({\mathrm{e}^{+}}{\mathrm{e}^{-}} \rightarrow {\mathrm{H}} {\mathrm{e}^{+}}{\mathrm{e}^{-}})$	7 fb	28 fb	48 fb
No. ${\mathrm{Z}} {\mathrm{H}}$ events	68,000	20,000	11,000
No. ${\mathrm{H}} {{\nu }}_{\!\mathrm{e}} {\bar{{\nu }}}_{\!\mathrm{e}}$ events	17,000	370,000	830,000
No. ${\mathrm{H}} {\mathrm{e}^{+}}{\mathrm{e}^{-}}$ events	3700	37,000	84,000

## Overview of Higgs production at CLIC

**Fig. 3:**
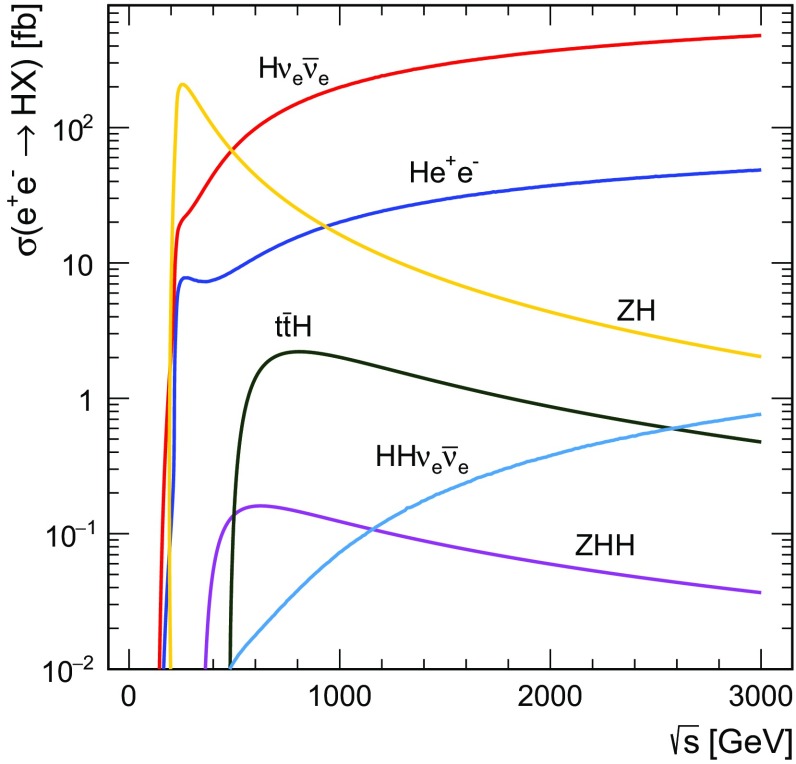
Cross section as a function of centre-of-mass energy for the main Higgs production processes at an ${\mathrm{e}^{+}}{\mathrm{e}^{-}}$ collider for a Higgs mass of $m_{{\mathrm{H}}} =126\,\text {GeV}$. The values shown correspond to unpolarised beams and do not include the effect of beamstrahlung

A high-energy ${\mathrm{e}^{+}}{\mathrm{e}^{-}}$ collider such as CLIC provides an experimental environment that allows the study of Higgs boson properties with high precision. The evolution of the leading-order ${\mathrm{e}^{+}}{\mathrm{e}^{-}}$ Higgs production cross sections with centre-of-mass energy, as computed using the Whizard 1.95 [20] program, is shown in Fig. 3 for a Higgs boson mass of $126\,\text {GeV}$ [21].

**Fig. 4:**
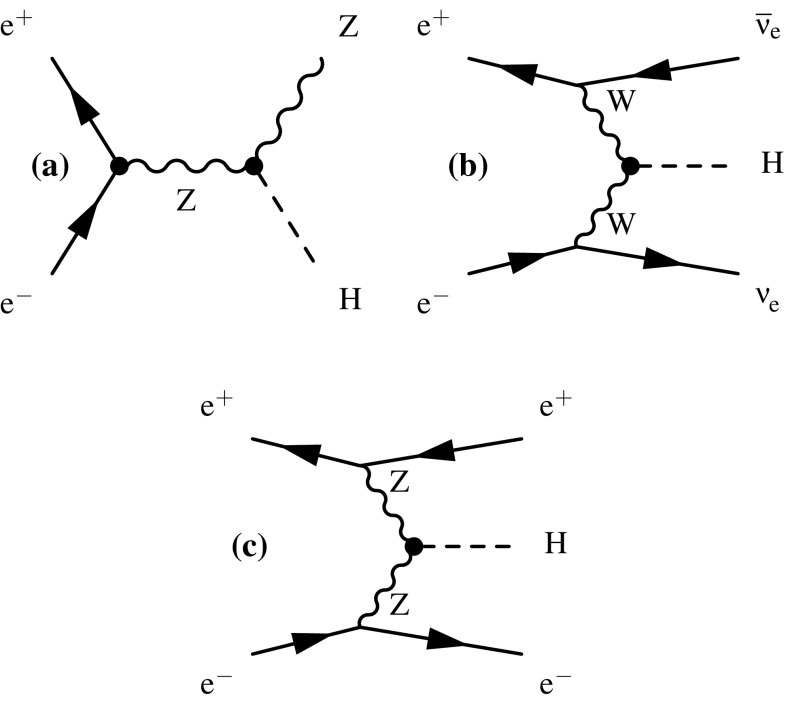
Leading-order Feynman diagrams of the highest cross section Higgs production processes at CLIC; Higgsstrahlung (**a**), ${\mathrm{W}} {\mathrm{W}}$-fusion (**b**) and ${\mathrm{Z}} {\mathrm{Z}}$-fusion (**c**)

**Fig. 5:**
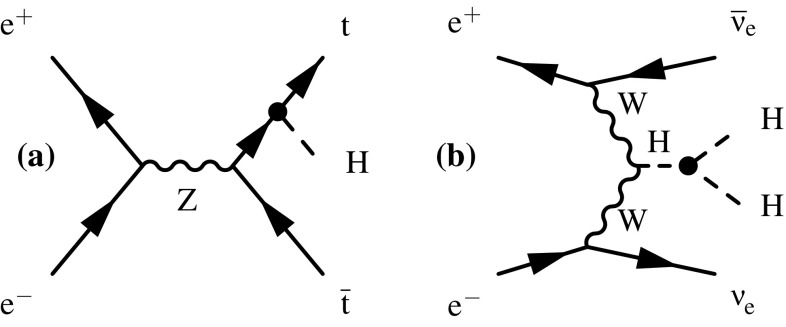
Feynman diagrams of the leading-order processes at CLIC involving (**a**) the top Yukawa coupling $g_{\mathrm{Htt}}$, and (**b**) the Higgs boson trilinear self-coupling $\lambda$

The Feynman diagrams for the three highest cross section Higgs production processes at CLIC are shown in Fig. 4. At $\sqrt{s} \approx 350\,\text {GeV}$, the Higgsstrahlung process (${\mathrm{e}^{+}}{\mathrm{e}^{-}} \rightarrow {\mathrm{Z}} {\mathrm{H}}$) has the largest cross section, but the ${\mathrm{W}} {\mathrm{W}}$-fusion process (${\mathrm{e}^{+}}{\mathrm{e}^{-}} \rightarrow {\mathrm{H}} {{\nu }}_{\!\mathrm{e}} {\bar{{\nu }}}_{\!\mathrm{e}}$) is also significant. The combined study of these two processes probes the Higgs boson properties (width and branching ratios) in a model-independent manner. In the higher energy stages of CLIC operation ($\sqrt{s} = 1.4\,\text {TeV}$ and $3\,\text {TeV}$), Higgs production is dominated by the ${\mathrm{W}} {\mathrm{W}}$-fusion process, with the ${\mathrm{Z}} {\mathrm{Z}}$-fusion process (${\mathrm{e}^{+}}{\mathrm{e}^{-}} \rightarrow {\mathrm{H}} {\mathrm{e}^{+}}{\mathrm{e}^{-}}$) also becoming significant. Here the increased ${\mathrm{W}} {\mathrm{W}}$-fusion cross section, combined with the high luminosity of CLIC, results in large data samples, allowing precise $\mathcal{{O}}(1\%)$ measurements of the couplings of the Higgs boson to both fermions and gauge bosons. In addition to the main Higgs production channels, rarer processes such as ${\mathrm{e}^{+}}{\mathrm{e}^{-}} \rightarrow \mathrm{t} {\bar{\mathrm{t}}} {\mathrm{H}}$ and ${\mathrm{e}^{+}}{\mathrm{e}^{-}} \rightarrow {\mathrm{H}} {\mathrm{H}} {{\nu }}_{\!\mathrm{e}} {\bar{{\nu }}}_{\!\mathrm{e}}$, provide access to the top Yukawa coupling and the Higgs trilinear self-coupling. Feynman diagrams for these processes are shown in Fig. 5. In all cases, the Higgs production cross sections can be increased with polarised electron (and positron) beams as discussed in Sect. 3.2.

Table 1 lists the expected numbers of ${\mathrm{Z}} {\mathrm{H}}$, ${\mathrm{H}} {{\nu }}_{\!\mathrm{e}} {\bar{{\nu }}}_{\!\mathrm{e}}$ and ${\mathrm{H}} {\mathrm{e}^{+}}{\mathrm{e}^{-}}$ events for the three main CLIC centre-of-mass energy stages. These numbers account for the effect of beamstrahlung and initial state radiation (ISR), which result in a tail in the distribution of the effective centre-of-mass energy $\sqrt{s^\prime }$. The impact of beamstrahlung on the expected numbers of events is mostly small. For example, it results in an approximately $10\%$ reduction in the numbers of ${\mathrm{H}} {{\nu }}_{\!\mathrm{e}} {\bar{{\nu }}}_{\!\mathrm{e}}$ events at $\sqrt{s} > 1\,\text {TeV}$ (compared to the beam spectrum with ISR alone), because the cross section rises relatively slowly with $\sqrt{s}$. The reduction of the effective centre-of-mass energies due to ISR and beamstrahlung increases the ${\mathrm{Z}} {\mathrm{H}}$ cross section at $\sqrt{s} = 1.4$ and $3\,\text {TeV}$.

**Fig. 6:**
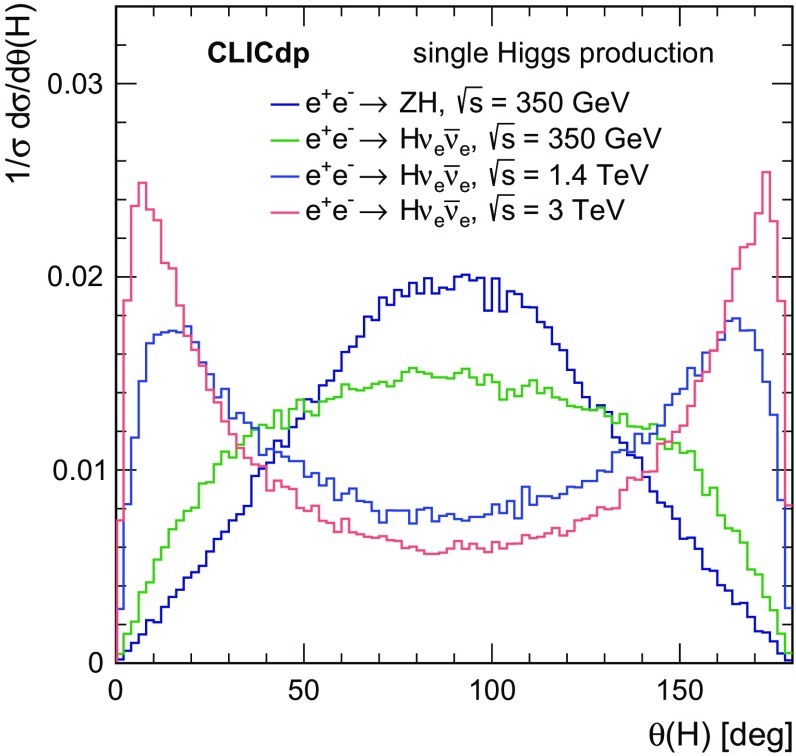
Polar angle distributions for single Higgs events at $\sqrt{s} = 350\,\text {GeV}$, 1.4 and $3\,\text {TeV}$, including the effects of the CLIC beamstrahlung spectrum and ISR. The distributions are normalised to unity

The polar angle distributions for single Higgs production obtained using Whizard 1.95 [20] for the CLIC centre-of-mass energies are shown in Fig. 6. Most Higgs bosons produced at $\sqrt{s} = 350\,\text {GeV}$ can be reconstructed in the central parts of the detectors while Higgs bosons produced in the ${\mathrm{W}} {\mathrm{W}}$-fusion process and their decay products tend towards the beam axis with increasing energy. Hence good detector capabilities in the forward regions are crucial at $\sqrt{s} = 1.4$ and $3\,\text {TeV}$.

A SM Higgs boson with mass of $m_{{\mathrm{H}}} =126\,\text {GeV}$ has a wide range of decay modes, as listed in Table 2, providing the possibility to test the SM predictions for the couplings of the Higgs to both gauge bosons and to fermions [22]. All the modes listed in Table 2 are accessible at CLIC.

**Table 2:** The investigated SM Higgs decay modes and their branching ratios as well as the total Higgs width for $m_{{\mathrm{H}}} =126\,\text {GeV}$ [22]

Decay mode	Branching ratio
${\mathrm{H}} \rightarrow {\mathrm{b}} {\bar{\mathrm{b}}}$	56.1%
${\mathrm{H}} \rightarrow {\mathrm{W}} {\mathrm{W}} ^*$	23.1%
${\mathrm{H}} \rightarrow {\mathrm{g}} {\mathrm{g}}$	8.5%
${\mathrm{H}} \rightarrow \uptau ^{+} \uptau ^{-}$	6.2%
${\mathrm{H}} \rightarrow {\mathrm{c}} {\bar{{\mathrm{c}}}}$	2.8%
${\mathrm{H}} \rightarrow {\mathrm{Z}} {\mathrm{Z}} ^*$	2.9%
${\mathrm{H}} \rightarrow {\upgamma } {\upgamma }$	0.23%
${\mathrm{H}} \rightarrow {\mathrm{Z}} {\upgamma }$	0.16%
${\mathrm{H}} \rightarrow {{{\upmu }}^{+} {{\upmu }}^{-}}$	0.021%
$\varGamma _{{\mathrm{H}}}$	4.2 $\text {MeV}$

### Motivation for $\sqrt{s} =350$ GeV CLIC operation

The choice of the CLIC energy stages is motivated by the desire to pursue a programme of precision Higgs physics and to operate the machine above $1\,\text {TeV}$ at the earliest possible time; no CLIC operation is foreseen below the top-pair production threshold.

From the Higgs physics perspective, operation at energies much below $1\,\text {TeV}$ is motivated by the direct and model-independent measurement of the coupling of the Higgs boson to the ${\mathrm{Z}}$, which can be obtained from the recoil mass distribution in ${\mathrm{Z}} {\mathrm{H}} \rightarrow {\mathrm{e}^{+}}{\mathrm{e}^{-}} {\mathrm{H}}$, ${\mathrm{Z}} {\mathrm{H}} \rightarrow {{{\upmu }}^{+} {{\upmu }}^{-}} {\mathrm{H}}$ and ${\mathrm{Z}} {\mathrm{H}} \rightarrow \mathrm{q}{\bar{\mathrm{q}}} {\mathrm{H}}$ production (see Sects. 5.1.1, 5.1.2). These measurements play a central role in the determination of the Higgs couplings at an ${\mathrm{e}^{+}}{\mathrm{e}^{-}}$ collider.

However, from a Higgs physics perspective, there is no advantage to running CLIC at around $\sqrt{s} = 250\,\text {GeV}$ where the ${\mathrm{Z}} {\mathrm{H}}$ production cross section is larger, compared to running at $\sqrt{s} = 350\,\text {GeV}$. Firstly, the reduction in cross section at $\sqrt{s} = 350\,\text {GeV}$ is compensated, in part, by the increased instantaneous luminosity achievable at a higher centre-of-mass energy. The instantaneous luminosity scales approximately linearly with the centre-of-mass energy, $\mathcal{{L}} \propto \gamma _{\mathrm{e}}$, where $\gamma _{\mathrm{e}}$ is the Lorentz factor for the beam electrons/positrons. For this reason, the precision on the coupling $g_{{\mathrm{H}} {\mathrm{Z}} {\mathrm{Z}}}$ at $350\,\text {GeV}$ is comparable to that achievable at $250\,\text {GeV}$ for the same period of operation. Secondly, the additional boost of the ${\mathrm{Z}}$ and ${\mathrm{H}}$ at $\sqrt{s} = 350\,\text {GeV}$ provides greater separation between the final-state jets from ${\mathrm{Z}}$ and ${\mathrm{H}}$ decays. Consequently, the measurements of $\sigma ({\mathrm{Z}} {\mathrm{H}})\times BR ({\mathrm{H}} \rightarrow X)$ are more precise at $\sqrt{s} = 350\,\text {GeV}$. Thirdly, and most importantly, operation of CLIC at $\sqrt{s} \approx 350\,\text {GeV}$ provides access to the ${\mathrm{e}^{+}}{\mathrm{e}^{-}} \rightarrow {\mathrm{H}} {{\nu }}_{\!\mathrm{e}} {\bar{{\nu }}}_{\!\mathrm{e}}$ fusion process; this improves the precision with which the total decay width $\varGamma _{{\mathrm{H}}}$ can be determined at CLIC. For the above reasons, the preferred option for the first stage of CLIC operation is $\sqrt{s} \approx 350\,\text {GeV}$.

Another advantage of $\sqrt{s} \approx 350\,\text {GeV}$ is that detailed studies of the top-pair production process can be performed in the initial stage of CLIC operation. Finally, the Higgs boson mass can be measured at $\sqrt{s} = 350\,\text {GeV}$ with similar precision as at $\sqrt{s} = 250\,\text {GeV}$.

### Impact of beam polarisation

The majority of CLIC Higgs physics studies presented in this paper are performed assuming unpolarised ${\mathrm{e}}^{+}$ and $\mathrm{e}^{-}$ beams. However, in the baseline CLIC design, the electron beam can be polarised up to $\pm 80\%$. There is also the possibility of positron polarisation at a lower level, although positron polarisation is not part of the baseline CLIC design. For an electron polarisation of $P_-$ and positron polarisation of $P_+$, the relative fractions of collisions in the different helicity states are:

$$\begin{aligned} {\mathrm{e}_{\mathrm{R}}^{-}} {\mathrm{e}_{\mathrm{R}}^{+}} \,:\,\ {\frac{1}{4}}(1+P_-)(1+P_+),&\ \ \ \ {\mathrm{e}_{\mathrm{R}}^{-}} \mathrm{e}_{\mathrm{L}}^{+}: \ {\frac{1}{4}}(1+P_-)(1-P_+), \\ \mathrm{e}_{\mathrm{L}}^{-} {\mathrm{e}_{\mathrm{R}}^{+}} \,:\,\ {\frac{1}{4}}(1-P_-)(1+P_+),&\ \ \ \ \mathrm{e}_{\mathrm{L}}^{-} \mathrm{e}_{\mathrm{L}}^{+}: \ {\frac{1}{4}}(1-P_-)(1-P_+). \end{aligned}$$

By selecting different beam polarisations it is possible to enhance/suppress different physical processes. The chiral nature of the weak coupling to fermions results in significant possible enhancements in ${\mathrm{W}} {\mathrm{W}}$-fusion Higgs production, as indicated in Table 3. The potential gains for the *s*-channel Higgsstrahlung process, ${\mathrm{e}^{+}}{\mathrm{e}^{-}} \rightarrow {\mathrm{Z}} {\mathrm{H}}$, are less significant, and the dependence of the ${\mathrm{e}^{+}}{\mathrm{e}^{-}} \rightarrow {\mathrm{H}} {\mathrm{e}^{+}}{\mathrm{e}^{-}}$ cross section on beam polarisation is even smaller. In practice, the balance between operation with different beam polarisations will depend on the CLIC physics programme taken as a whole, including the searches for and potential measurements of BSM particle production.

**Table 3:** The dependence of the event rates for the *s*-channel ${\mathrm{e}^{+}}{\mathrm{e}^{-}} \rightarrow {\mathrm{Z}} {\mathrm{H}}$ process and the pure *t*-channel ${\mathrm{e}^{+}}{\mathrm{e}^{-}} \rightarrow {\mathrm{H}} {{\nu }}_{\!\mathrm{e}} {\bar{{\nu }}}_{\!\mathrm{e}}$ and ${\mathrm{e}^{+}}{\mathrm{e}^{-}} \rightarrow {\mathrm{H}} {\mathrm{e}^{+}}{\mathrm{e}^{-}}$ processes for several example beam polarisations. The scale factors assume an effective weak mixing angle given by $\sin ^2\theta _{{\mathrm{W}}}^\text {eff} = 0.23146$ [23]. The numbers are approximate as they do not account for interference between ${\mathrm{e}^{+}}{\mathrm{e}^{-}} \!\rightarrow {\mathrm{Z}} {\mathrm{H}} \!\rightarrow {{\nu }}_{\!\mathrm{e}} {\bar{{\nu }}}_{\!\mathrm{e}} {\mathrm{H}}$ and ${\mathrm{e}^{+}}{\mathrm{e}^{-}} \!\rightarrow {\mathrm{H}} {{\nu }}_{\!\mathrm{e}} {\bar{{\nu }}}_{\!\mathrm{e}}$

Polarisation	Scaling factor		
$P(\mathrm{e}^-):P({\mathrm{e}}^{+})$	${\mathrm{e}^{+}}{\mathrm{e}^{-}} \!\rightarrow {\mathrm{Z}} {\mathrm{H}} \!\!$	${\mathrm{e}^{+}}{\mathrm{e}^{-}} \!\rightarrow {\mathrm{H}} {{\nu }}_{\!\mathrm{e}} {\bar{{\nu }}}_{\!\mathrm{e}} \!\!$	${\mathrm{e}^{+}}{\mathrm{e}^{-}} \!\rightarrow {\mathrm{H}} {\mathrm{e}^{+}}{\mathrm{e}^{-}} \!\!$
Unpolarised	1.00	1.00	1.00
$-80\%\,:\,0\%$	1.12	1.80	1.12
$-80\%\,:\,+30\%$	1.40	2.34	1.17
$-80\%\,:\,-30\%$	0.83	1.26	1.07
$+80\%\,:\,0\%$	0.88	0.20	0.88
$+80\%\,:\,+30\%$	0.69	0.26	0.92
$+80\%\,:\,-30\%$	1.08	0.14	0.84

### Overview of Higgs measurements at $\varvec{\sqrt{s}}=350$ GeV

The Higgsstrahlung process, ${\mathrm{e}^{+}}{\mathrm{e}^{-}} \rightarrow {\mathrm{Z}} {\mathrm{H}}$, provides an opportunity to study the couplings of the Higgs boson in an essentially model-independent manner. Such a model-independent measurement is unique to a lepton collider. Higgsstrahlung events can be selected based solely on the measurement of the four-momentum of the ${\mathrm{Z}}$ boson through its decay products, while the invariant mass of the system recoiling against the ${\mathrm{Z}}$ boson peaks at $m_{{\mathrm{H}}}$. The most distinct event topologies occur for ${\mathrm{Z}} \rightarrow {\mathrm{e}^{+}}{\mathrm{e}^{-}}$ and ${\mathrm{Z}} \rightarrow {{{\upmu }}^{+} {{\upmu }}^{-}}$ decays, which can be identified by requiring that the di-lepton invariant mass is consistent with $m_{{\mathrm{Z}}}$ (see Sect. 5.1.1). SM background cross sections are relatively low. A slightly less clean, but more precise, measurement is obtained from the recoil mass analysis for ${\mathrm{Z}} \rightarrow \mathrm{q}{\bar{\mathrm{q}}}$ decays (see Sect. 5.1.2).

Recoil-mass studies provide an absolute measurement of the total ${\mathrm{Z}} {\mathrm{H}}$ production cross section and a model-independent measurement of the coupling of the Higgs to the ${\mathrm{Z}}$ boson, $g_{{\mathrm{H}} {\mathrm{Z}} {\mathrm{Z}}}$. The combination of the leptonic and hadronic decay channels allows $g_{{\mathrm{H}} {\mathrm{Z}} {\mathrm{Z}}}$ to be determined with a precision of $0.8\%$. In addition, the recoil mass from ${\mathrm{Z}} \rightarrow \mathrm{q}{\bar{\mathrm{q}}}$ decays provides a direct search for possible Higgs decays to invisible final states, and can be used to constrain the invisible decay width of the Higgs, $\varGamma _\text {invis}$.

By identifying the individual final states for different Higgs decay modes, precise measurements of the Higgs boson branching fractions can be made. Because of the high flavour tagging efficiencies [11] achievable at CLIC, the ${\mathrm{H}} \rightarrow {\mathrm{b}} {\bar{\mathrm{b}}}$ and ${\mathrm{H}} \rightarrow {\mathrm{c}} {\bar{{\mathrm{c}}}}$ decays can be cleanly separated. Neglecting the Higgs decays into light quarks, the branching ratio of ${\mathrm{H}} \rightarrow {\mathrm{g}} {\mathrm{g}}$ can also be inferred and ${\mathrm{H}} \rightarrow \uptau ^{+} \uptau ^{-}$ decays can be identified.

Although the cross section is lower, the *t*-channel ${\mathrm{W}} {\mathrm{W}}$-fusion process ${\mathrm{e}^{+}}{\mathrm{e}^{-}} \rightarrow {\mathrm{H}} {{\nu }}_{\!\mathrm{e}} {\bar{{\nu }}}_{\!\mathrm{e}}$ is an important part of the CLIC Higgs physics programme at $\sqrt{s} \approx 350\,\text {GeV}$. Because the visible final state consists of the Higgs boson decay products alone, the direct reconstruction of the invariant mass of the Higgs boson or its decay products plays a central role in the event selection. The combination of Higgs production and decay data from Higgsstrahlung and ${\mathrm{W}} {\mathrm{W}}$-fusion processes provides a model-independent extraction of Higgs couplings.

#### Extraction of Higgs couplings

At the LHC, only the ratios of the Higgs boson couplings can be inferred from the data in a model-independent way.

In contrast, at an electron–positron collider such as CLIC, absolute measurements of the couplings to the Higgs boson can be determined using the total ${\mathrm{e}^{+}}{\mathrm{e}^{-}} \rightarrow {\mathrm{Z}} {\mathrm{H}}$ cross section determined from recoil mass analyses. This allows the coupling of the Higgs boson to the ${\mathrm{Z}}$ to be determined with a precision of better than $1\%$ in an essentially model-independent manner. Once the coupling to the ${\mathrm{Z}}$ is known, the Higgs coupling to the ${\mathrm{W}}$ can be determined from, for example, the ratios of Higgsstrahlung to ${\mathrm{W}} {\mathrm{W}}$-fusion cross sections:

$$\begin{aligned} \frac{\sigma ({\mathrm{e}^{+}}{\mathrm{e}^{-}} \rightarrow {\mathrm{Z}} {\mathrm{H}})\times BR ({\mathrm{H}} \rightarrow {\mathrm{b}} {\bar{\mathrm{b}}}) }{ \sigma {({\mathrm{e}^{+}}{\mathrm{e}^{-}} \rightarrow {{\nu }}_{\!\mathrm{e}} {\bar{{\nu }}}_{\!\mathrm{e}} {\mathrm{H}})} \times BR ({\mathrm{H}} \rightarrow {\mathrm{b}} {\bar{\mathrm{b}}}) } \propto \left( \frac{g_{{\mathrm{H}} {\mathrm{Z}} {\mathrm{Z}}}}{g_{{\mathrm{H}} {\mathrm{W}} {\mathrm{W}}}} \right) ^2. \end{aligned}$$

Knowledge of the Higgs total decay width, extracted from the data, allows absolute measurements of the other Higgs couplings.

For a Higgs boson mass of around $126\,\text {GeV}$, the total Higgs decay width in the SM ($\varGamma _{{\mathrm{H}}}$) is less than $5\,\text {MeV}$ and cannot be measured directly at an ${\mathrm{e}^{+}}{\mathrm{e}^{-}}$ linear collider. However, as the absolute couplings of the Higgs boson to the ${\mathrm{Z}}$ and ${\mathrm{W}}$ can be determined, the total decay width of the Higgs boson can be determined from ${\mathrm{H}} \rightarrow {\mathrm{W}} {\mathrm{W}} ^*$ or ${\mathrm{H}} \rightarrow {\mathrm{Z}} {\mathrm{Z}} ^*$ decays. For example, the measurement of the Higgs decay to ${\mathrm{W}} {\mathrm{W}} ^*$ in the ${\mathrm{W}} {\mathrm{W}}$-fusion process determines:

$$\begin{aligned} \sigma ({\mathrm{H}} {{\nu }}_{\!\mathrm{e}} {\bar{{\nu }}}_{\!\mathrm{e}})\times BR ({\mathrm{H}} \rightarrow {\mathrm{W}} {\mathrm{W}} ^*) \propto \frac{g^4_{{\mathrm{H}} {\mathrm{W}} {\mathrm{W}}}}{\varGamma _{{\mathrm{H}}}}, \end{aligned}$$

and thus the total width can be determined utilising the model-independent measurement of $g_{{\mathrm{H}} {\mathrm{W}} {\mathrm{W}}}$. In practice, a fit (see Sect. 12) is performed to all of the experimental measurements involving the Higgs boson couplings.

### Overview of Higgs measurements at $\sqrt{s} > 1$ TeV

For CLIC operation above $1\,\text {TeV}$, the large number of Higgs bosons produced in the ${\mathrm{W}} {\mathrm{W}}$-fusion process allow relative couplings of the Higgs boson to the ${\mathrm{W}}$ and ${\mathrm{Z}}$ bosons to be determined at the $\mathcal{{O}}(1\%)$ level. These measurements provide a strong test of the SM prediction for:

$$\begin{aligned} g_{{\mathrm{H}} {\mathrm{W}} {\mathrm{W}}} / g_{{\mathrm{H}} {\mathrm{Z}} {\mathrm{Z}}} = \cos ^2\theta _\mathrm {W}, \end{aligned}$$

where $\theta _\mathrm {W}$ is the weak-mixing angle. Furthermore, the exclusive Higgs decay modes can be studied with significantly higher precision than at $\sqrt{s} =350\,\text {GeV}$. For example, CLIC operating at $3\,\text {TeV}$ yields a statistical precision of $2\%$ on the ratio $g_{{\mathrm{H}} {\mathrm{c}} {\mathrm{c}}}/g_{{\mathrm{H}} {\mathrm{b}} {\mathrm{b}}}$, providing a direct comparison of the SM coupling predictions for up-type and down-type quarks. In the context of the model-independent measurements of the Higgs branching ratios, the measurement of $\sigma ({\mathrm{H}} {{\nu }}_{\!\mathrm{e}} {\bar{{\nu }}}_{\!\mathrm{e}})\times BR ({\mathrm{H}} \rightarrow {\mathrm{W}} {\mathrm{W}} ^*)$ is particularly important. For CLIC operation at $\sqrt{s} \approx 1.4\,\text {TeV}$, the large number of events allows this cross section to be determined with a precision of $1\%$ (see Sect. 6.3). When combined with the measurements at $\sqrt{s} \approx 350\,\text {GeV}$, this places a strong constraint on $\varGamma _{{\mathrm{H}}}$.

Although the ${\mathrm{W}} {\mathrm{W}}$-fusion process has the largest cross section for Higgs production above $1\,\text {TeV}$, other processes are also important. For example, measurements of the ${\mathrm{Z}} {\mathrm{Z}}$-fusion process provide further constraints on the $g_{{\mathrm{H}} {\mathrm{Z}} {\mathrm{Z}}}$ coupling. Moreover, CLIC operation at $\sqrt{s} = 1.4\,\text {TeV}$ enables a determination of the top Yukawa coupling from the process ${\mathrm{e}^{+}}{\mathrm{e}^{-}} \rightarrow \mathrm{t} {\bar{\mathrm{t}}} {\mathrm{H}} \rightarrow {\mathrm{b}} {\mathrm{W}} ^+{\bar{\mathrm{b}}} {\mathrm{W}} ^-{\mathrm{H}}$ with a precision of $4.2\%$ (see Sect. 8). Finally, the self-coupling of the Higgs boson at the ${\mathrm{H}} {\mathrm{H}} {\mathrm{H}}$ vertex is measurable in 1.4 and $3\,\text {TeV}$ operation.

In the SM, the Higgs boson originates from a doublet of complex scalar fields $\phi$ described by the potential:

$$\begin{aligned} V(\phi ) = \mu ^2\phi ^\dagger \phi + \lambda (\phi ^\dagger \phi )^2 , \end{aligned}$$

where $\mu$ and $\lambda$ are the parameters of the Higgs potential, with $\mu ^2 < 0$ and $\lambda > 0$. The measurement of the strength of the Higgs self-coupling provides direct access to the coupling $\lambda$ assumed in the Higgs mechanism. For $m_{{\mathrm{H}}}$ of around $126\,\text {GeV}$, the measurement of the Higgs boson self-coupling at the LHC will be extremely challenging, even with $3000\,\text {fb}^{-1}$ of data (see for example [24]). At a linear collider, the trilinear Higgs self-coupling can be measured through the ${\mathrm{e}^{+}}{\mathrm{e}^{-}} \rightarrow {\mathrm{Z}} {\mathrm{H}} {\mathrm{H}}$ and ${\mathrm{e}^{+}}{\mathrm{e}^{-}} \rightarrow {\mathrm{H}} {\mathrm{H}} {{\nu }}_{\!\mathrm{e}} {\bar{{\nu }}}_{\!\mathrm{e}}$ processes. The ${\mathrm{e}^{+}}{\mathrm{e}^{-}} \rightarrow {\mathrm{Z}} {\mathrm{H}} {\mathrm{H}}$ process at $\sqrt{s} =500\,\text {GeV}$ has been studied in the context of the ILC, where the results show that a very large integrated luminosity is required [25]. However for $\sqrt{s} \ge 1\,\text {TeV}$, the sensitivity for the process ${\mathrm{e}^{+}}{\mathrm{e}^{-}} \rightarrow {\mathrm{H}} {\mathrm{H}} {{\nu }}_{\!\mathrm{e}} {\bar{{\nu }}}_{\!\mathrm{e}}$ increases with increasing centre-of-mass energy and the measurement of the Higgs boson self-coupling (see Sect. 9) forms a central part of the CLIC Higgs physics programme. Ultimately a precision of approximately $20\%$ on $\lambda$ can be achieved.

## Event generation, detector simulation and reconstruction

The results presented in this paper are based on detailed Monte Carlo (MC) simulation studies including the generation of a complete set of relevant SM background processes, Geant4  [26, 27] based simulations of the CLIC detector concepts, and a full reconstruction of the simulated events.

### Event generation

Because of the presence of beamstrahlung photons in the colliding electron and positron beams, it is necessary to generate MC event samples for ${\mathrm{e}^{+}}{\mathrm{e}^{-}}$, ${\mathrm{e}}^{+} {\upgamma }$, ${\upgamma } \mathrm{e}^-$, and ${\upgamma } {\upgamma }$ interactions. The main physics backgrounds, with up to six particles in the final state, are generated using the Whizard 1.95 [20] program. In all cases the expected energy spectra for the CLIC beams, including the effects from beamstrahlung and the intrinsic machine energy spread, are used for the initial-state electrons, positrons and beamstrahlung photons. In addition, low-$Q^2$ processes with quasi-real photons are described using the Weizsäcker-Williams approximation as implemented in Whizard. The process of fragmentation and hadronisation is simulated using Pythia 6.4 [28] with a parameter set tuned to OPAL ${\mathrm{e}^{+}}{\mathrm{e}^{-}}$ data recorded at LEP [29] (see [11] for details). The decays of $\uptau$ leptons are simulated using Tauola  [30]. The mass of the Higgs boson is taken to be $126\,\text {GeV}$ 2 and the decays of the Higgs boson are simulated using Pythia with the branching fractions listed in [22]. The events from the different Higgs production channels are simulated separately. The background samples do not include Higgs processes. MC samples for the measurement of the top Yukawa coupling measurement (see Sect. 8) with eight final-state fermions are obtained using the PhysSim  [31] package; again Pythia is used for fragmentation, hadronisation and the Higgs boson decays.

### Simulation and reconstruction

The Geant4 detector simulation toolkits Mokka  [32] and Slic  [33] are used to simulate the detector response to the generated events in the CLIC_ILD and CLIC_SiD concepts, respectively. The QGSP_BERT physics list is used to model the hadronic interactions of particles in the detectors. The digitisation, namely the translation of the raw simulated energy deposits into detector signals, and the event reconstruction are performed using the Marlin  [34] and org.lcsim [35] software packages. Particle flow reconstruction is performed using PandoraPFA  [15, 16, 36].

Vertex reconstruction and heavy flavour tagging are performed using the LcfiPlus program [37]. This consists of a topological vertex finder that reconstructs secondary interactions, and a multivariate classifier that combines several jet-related variables such as track impact parameter significance, decay length, number of tracks in vertices, and vertex masses, to tag bottom, charm, and light-quark jets. The detailed training of the multivariate classifiers for the flavour tagging is performed separately for each centre-of-mass energy and each final state of interest.

Because of the 0.5 ns bunch spacing in the CLIC beams, the pile-up of beam-induced backgrounds can affect the event reconstruction and needs to be accounted for. Realistic levels of pile-up from the most important beam-induced background, the ${\upgamma } {\upgamma } \rightarrow \text {hadrons}$ process, are included in all the simulated event samples to ensure that the impact on the event reconstruction is correctly modelled. The ${\upgamma } {\upgamma } \rightarrow \text {hadrons}$ events are simulated separately and a randomly chosen subset, corresponding to 60 bunch crossings, is superimposed on the physics event before the digitisation step [38]. 60 bunch crossings is equivalent to 30 ns, which is much longer than the assumed offline event reconstruction window of 10 ns around the hard physics event, so this is a good approximation [11]. For the $\sqrt{s} = 350\,\text {GeV}$ samples, where the background rates are lower, 300 bunch crossings are overlaid on the physics event. The impact of the background is small at $\sqrt{s} = 350\,\text {GeV}$, and is most significant at $\sqrt{s} =3\,\text {TeV}$, where approximately $1.2\,\text {TeV}$ of energy is deposited in the calorimeters in a time window of 10 ns. A dedicated reconstruction algorithm identifies and removes approximately $90\%$ of these out-of-time background particles using criteria based on the reconstructed transverse momentum $p_\mathrm {T}$ of the particles and the calorimeter cluster time. A more detailed description can be found in [11].

Jet finding is performed on the objects reconstructed by particle flow, using the FastJet  [39] package. Because of the presence of pile-up from ${\upgamma } {\upgamma } \rightarrow \text {hadrons}$, it was found that the Durham [40] algorithm employed at LEP is not optimal for CLIC studies. Instead, the hadron-collider inspired $k_t$ algorithm [41, 42], with the distance parameter *R* based on $\varDelta \eta$ and $\varDelta \phi$, is found to give better performance since it increases distances in the forward region, thus reducing the clustering of the (predominantly low transverse momentum) background particles together with those from the hard ${\mathrm{e}^{+}}{\mathrm{e}^{-}}$ interaction. Instead, particles that are found by the $k_t$ algorithm to be closer to the beam axis than to any other particles, and that are thus likely to have originated from beam-beam backgrounds, are removed from the event. As a result of using the *R*-based $k_t$ algorithm, the impact of the pile-up from ${\upgamma } {\upgamma } \rightarrow \text {hadrons}$ is largely mitigated, even without the timing and momentum cuts described above. Further details are given in [11]. The choice of *R* is optimised separately for different analyses. In many of the following studies, events are forced into a particular *N*-jet topology. The variable $y_{ij}$ is the smallest $k_{t}$ distance when combining *j* jets to $i = (j-1)$ jets. These resolution parameters are widely used in a number of event selections, allowing events to be categorised into topologically different final states. In several studies it is found to be advantageous first to apply the $k_t$ algorithm to reduce the beam-beam backgrounds, and then to use only the remaining objects as input to the Durham algorithm.

**Fig. 7:**
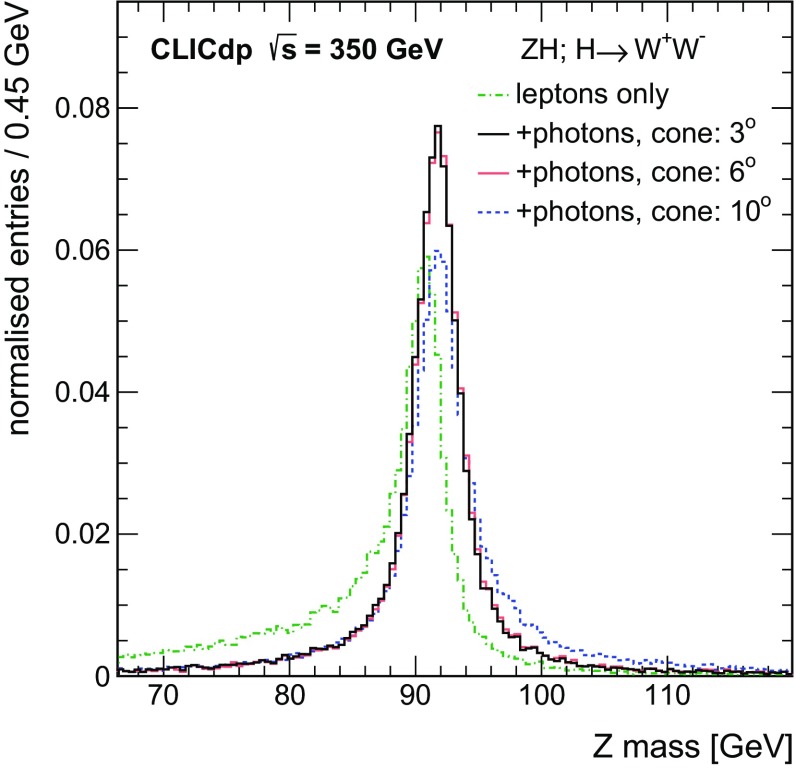
Reconstructed invariant mass of ${\mathrm{Z}} \rightarrow {\mathrm{e}^{+}}{\mathrm{e}^{-}}$ candidates in ${\mathrm{e}^{+}}{\mathrm{e}^{-}} \rightarrow {\mathrm{Z}} {\mathrm{H}} \rightarrow {\mathrm{Z}} {\mathrm{W}} {\mathrm{W}} ^*$ events at $\sqrt{s} =350\,\text {GeV}$. Bremsstrahlung photons in cones of different opening angles around the electron direction are recovered as described in the text. All distributions are normalised to unity

To recover the effect of bremsstrahlung photons radiated from reconstructed leptons, all photons in a cone around the flight direction of a lepton candidate are added to its four-momentum. The impact of the bremsstrahlung recovery on the reconstruction of the ${\mathrm{Z}} \rightarrow {\mathrm{e}^{+}}{\mathrm{e}^{-}}$ decays is illustrated in Fig. 7. The bremsstrahlung effect leads to a tail at lower values in the ${\mathrm{Z}}$ candidate invariant mass distribution. This loss can be recovered by the procedure described above. It is also visible that a too large opening angle of the recovery cone leads to a tail at higher masses; typically, an opening angle of $3^\circ$ is chosen.

The event simulation and reconstruction of the large data samples used in this study was performed using the iLCDirac [43, 44] grid production tools.

## Higgs production at $\sqrt{s} =350$ GeV

The study of the Higgsstrahlung process is central to the precision Higgs physics programme at any future high-energy electron–positron collider [45]. This section presents studies of ${\mathrm{e}^{+}}{\mathrm{e}^{-}} \rightarrow {\mathrm{Z}} {\mathrm{H}}$ at $\sqrt{s} =350\,\text {GeV}$ with a focus on model-independent measurements of ${\mathrm{Z}} {\mathrm{H}}$ production from the kinematic properties of the Z decay products. Complementary information obtained from Higgs production through WW-fusion at $\sqrt{s} =350\,\text {GeV}$ is also presented. All analyses at $\sqrt{s} =350\,\text {GeV}$ described in this paper use the CLIC_ILD detector model.

### Recoil mass measurements of ${\mathrm{e}^{+}}{\mathrm{e}^{-}} \rightarrow {\mathrm{Z}} {\mathrm{H}}$

In the process ${\mathrm{e}^{+}}{\mathrm{e}^{-}} \rightarrow {\mathrm{Z}} {\mathrm{H}}$, it is possible to identify efficiently ${\mathrm{Z}} \rightarrow {\mathrm{e}^{+}}{\mathrm{e}^{-}}$ and ${\mathrm{Z}} \rightarrow {{{\upmu }}^{+} {{\upmu }}^{-}}$ decays with a selection efficiency that is essentially independent of the ${\mathrm{H}}$ decay mode. The four-momentum of the (Higgs boson) system recoiling against the ${\mathrm{Z}}$ can be obtained from $E_{rec} = \sqrt{s}- E_{{\mathrm{Z}}}$ and $\mathbf {p}_{rec} = -\mathbf {p}_{{\mathrm{Z}}}$, and the recoil mass, $m_\mathrm {rec}$, peaks sharply around $m_{{\mathrm{H}}}$. The recoil mass analysis for leptonic decays of the ${\mathrm{Z}}$ is described in Sect. 5.1.1. While these measurements provide a clean model-independent probe of ${\mathrm{Z}} {\mathrm{H}}$ production, they are limited by the relatively small leptonic branching ratios of the ${\mathrm{Z}}$. Studies of ${\mathrm{Z}} {\mathrm{H}}$ production with ${\mathrm{Z}} \rightarrow \mathrm{q}{\bar{\mathrm{q}}}$ are inherently less clean, but are statistically more powerful. Despite the challenges related to the reconstruction of hadronic ${\mathrm{Z}}$ decays in the presence of various Higgs decay modes, a precise and nearly model-independent probe of ${\mathrm{Z}} {\mathrm{H}}$ production can be obtained by analysing the recoil mass in hadronic ${\mathrm{Z}}$ decays, as detailed in Sect. 5.1.2. When all these measurements are taken together, a model-independent measurement of the $g_{{\mathrm{H}} {\mathrm{Z}} {\mathrm{Z}}}$ coupling constant with a precision of $<1\%$ can be inferred [45].

#### Leptonic decays: ${\mathrm{Z}} \rightarrow {\mathrm{e}^{+}}{\mathrm{e}^{-}}$ and ${\mathrm{Z}} \rightarrow {{{\upmu }}^{+} {{\upmu }}^{-}}$

The signature for ${\mathrm{e}^{+}}{\mathrm{e}^{-}} \rightarrow {\mathrm{Z}} {\mathrm{H}}$ production with ${\mathrm{Z}} \rightarrow {\mathrm{e}^{+}}{\mathrm{e}^{-}}$ or ${\mathrm{Z}} \rightarrow {{{\upmu }}^{+} {{\upmu }}^{-}}$ is a pair of oppositely charged high-$p_\mathrm {T}$ leptons, with an invariant mass consistent with that of the ${\mathrm{Z}}$ boson, $m_{\mathrm{l} \mathrm{l}} \approx m_{{\mathrm{Z}}}$, and a recoil mass, calculated from the four-momenta of the leptons alone, consistent with the Higgs mass, $m_\mathrm {rec} \approx m_{{\mathrm{H}}}$ [46]. Backgrounds from two-fermion final states ${\mathrm{e}^{+}}{\mathrm{e}^{-}} \rightarrow {\mathrm{l}}^{+} {\mathrm{l}}^{-}$ ($\mathrm{l} = \mathrm{e}, {\upmu }, \uptau$) are trivial to remove. The dominant backgrounds are from four-fermion processes with final states consisting of a pair of oppositely-charged leptons and any other possible fermion pair. For both the ${{{{\upmu }}^{+} {{\upmu }}^{-}}} X$ and ${\mathrm{e}^{+}}{\mathrm{e}^{-}} X$ channels, the total four-fermion background cross section is approximately one thousand times greater than the signal cross section.

The event selection employs preselection cuts and a multivariate analysis. The preselection requires at least one negatively and one positively charged lepton of the lepton flavour of interest (muons or electrons) with an invariant mass loosely consistent with the mass of the ${\mathrm{Z}}$ boson, $40\,\text {GeV} {<} m_{\mathrm{l} \mathrm{l}} < 126\,\text {GeV}$. For signal events, the lepton identification efficiencies are $99\%$ for muons and $90\%$ for electrons. Backgrounds from two-fermion processes are essentially eliminated by requiring that the di-lepton system has $p_\mathrm {T} >60\,\text {GeV}$. Four-fermion backgrounds are suppressed by requiring $95\,\text {GeV} {<} m_\mathrm {rec} < 290\,\text {GeV}$. The lower bound suppresses ${\mathrm{e}^{+}}{\mathrm{e}^{-}} \rightarrow {\mathrm{Z}} {\mathrm{Z}}$ production. The upper bound is significantly greater than the Higgs boson mass, to allow for the possibility of ${\mathrm{Z}} {\mathrm{H}}$ production with ISR or significant beamstrahlung, which, in the recoil mass analysis, results in a tail to the recoil mass distribution, as it is the mass of the ${\mathrm{H}} {\upgamma }$ system that is estimated.

**Table 4:** Preselection and selection efficiencies for the ${\mathrm{Z}} {\mathrm{H}}$ signal and most important background processes in the leptonic recoil mass analysis. The numbers of events correspond to 500 $\text {fb}^{-1}$ at $\sqrt{s} =350\,\text {GeV}$

Process	$\sigma /\text {fb}$	$\varepsilon _\text {presel}$ (%)	$\varepsilon _\text {BDT}$ (%)	$N_\text {BDT}$
${\mathrm{Z}} {\mathrm{H}}; {\mathrm{Z}} \rightarrow {{{\upmu }}^{+} {{\upmu }}^{-}}$	4.6	84	65	1253
${{{{\upmu }}^{+} {{\upmu }}^{-}}}\text {f}\overline{\text {f}}$	4750	0.8	10	1905
${\mathrm{Z}} {\mathrm{H}}; {\mathrm{Z}} \rightarrow {\mathrm{e}^{+}}{\mathrm{e}^{-}}$	4.6	73	51	858
${\mathrm{e}^{+}}{\mathrm{e}^{-}} \text {f}\overline{\text {f}}$	4847	1.2	5.4	1558

**Fig. 8:**
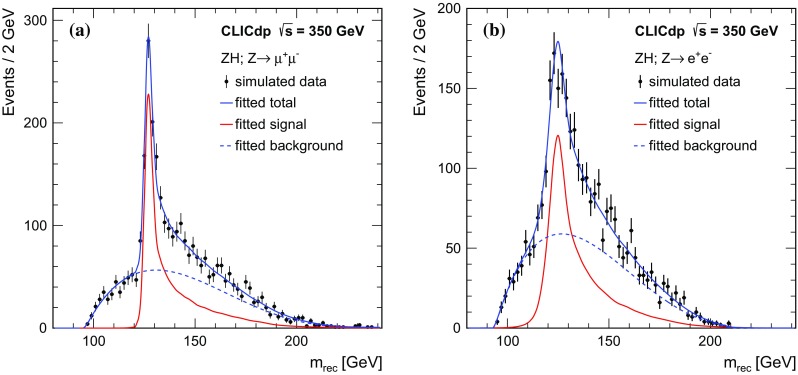
Reconstructed recoil mass distributions of ${\mathrm{e}^{+}}{\mathrm{e}^{-}} \rightarrow {\mathrm{Z}} {\mathrm{H}}$ events at $\sqrt{s} =350\,\text {GeV}$, where ${\mathrm{Z}} {\mathrm{H}} \rightarrow {{{\upmu }}^{+} {{\upmu }}^{-}} X$ (**a**) and ${\mathrm{Z}} {\mathrm{H}} \rightarrow {\mathrm{e}^{+}}{\mathrm{e}^{-}} X$ with bremsstrahlung recovery (**b**). All distributions are normalised to an integrated luminosity of $500\,\text {fb}^{-1}$

Events passing the preselection cuts are categorised using a multivariate analysis of seven discriminating variables: the transverse momentum ($p_\mathrm {T}$) and invariant mass ($m_{\mathrm{l} \mathrm{l}}$) of the candidate ${\mathrm{Z}}$; the cosine of the polar angle $(|\cos \theta |)$ of the candidate ${\mathrm{Z}}$; the acollinearity and acoplanarity of the leptons; the imbalance between the transverse momenta of the two selected leptons $({p_\mathrm {T}}_1 - {p_\mathrm {T}}_2)$; and the transverse momentum of the highest energy photon in the event. The event selection employs a Boosted Decision Tree (BDT) as implemented in Tmva  [47]. The resulting selection efficiencies are summarised in Table 4. For both final states, the number of selected background events is less than twice the number of selected signal events. The impact of the background is reduced using a fit to the recoil mass distribution.

A fit to the recoil mass distribution of the selected events (in both the ${\mathrm{Z}} \rightarrow {\mathrm{e}^{+}}{\mathrm{e}^{-}}$ and ${\mathrm{Z}} \rightarrow {{{\upmu }}^{+} {{\upmu }}^{-}}$ channels) is used to extract measurements of the ${\mathrm{Z}} {\mathrm{H}}$ production cross section and the Higgs boson mass. The shape of the background contribution is parameterised using a fourth order polynomial and the shape of the signal distribution is modelled using Simplified Kernel Estimation [48–50] that provides a description of the ${\mathrm{Z}} {\mathrm{H}}$ recoil mass distribution in which the Higgs mass can subsequently be varied. The accuracy with which the Higgs mass and the number of signal events (and hence the ${\mathrm{Z}} {\mathrm{H}}$ production cross section) can be measured, is determined using 1000 simulated test data samples. Each test sample was created by adding the high statistics selected signal sample (scaled to the correct normalisation) to the smooth fourth-order polynomial background,then applying Poisson fluctuations to individual bins to create a representative $500\,\text {fb}^{-1}$ data sample. Each of the 1000 simulated data samples created in this way is fitted allowing the Higgs mass, the signal normalisation and the background normalisation to vary. Figure 8a displays the results of fitting a typical test sample for the ${{{{\upmu }}^{+} {{\upmu }}^{-}}} X$ channel, while Fig. 8b displays the results for the ${\mathrm{e}^{+}}{\mathrm{e}^{-}} X$ channel. In the ${\mathrm{e}^{+}}{\mathrm{e}^{-}} X$ channel fits are performed with, and without, applying an algorithm to recover bremsstrahlung photons. The resulting measurement precisions for the ${\mathrm{Z}} {\mathrm{H}}$ cross section and the Higgs boson mass are summarised in Table 5. In the ${\mathrm{e}^{+}}{\mathrm{e}^{-}} X$ channel, the bremsstrahlung recovery leads to a moderate improvement on the expected precision for the cross section measurement and a similar degradation in the expected precision for the mass determination, because it significantly increases the number of events in the peak of the recoil mass distribution, but also increases the width of this peak. For an integrated luminosity of $500\,\text {fb}^{-1}$ at $\sqrt{s} =350\,\text {GeV}$, the combined precision on the Higgs boson mass is:

$$\begin{aligned} \varDelta (m_{{\mathrm{H}}}) = 110\,\text {MeV}, \end{aligned}$$

and the combined precision on the ${\mathrm{Z}} {\mathrm{H}}$ cross section is:

$$\begin{aligned} \frac{\varDelta {\sigma ({\mathrm{Z}} {\mathrm{H}})}}{\sigma ({\mathrm{Z}} {\mathrm{H}})}&= 3.8\%. \end{aligned}$$

The expected precision with (without) bremsstrahlung recovery in the ${\mathrm{e}^{+}}{\mathrm{e}^{-}} X$ channel was used in the combination for the cross section (mass).

**Table 5:** Summary of measurement precisions from the leptonic recoil mass analyses in the ${{{{\upmu }}^{+} {{\upmu }}^{-}}} X$ and ${\mathrm{e}^{+}}{\mathrm{e}^{-}} X$ channels for an integrated luminosity of $500\,\text {fb}^{-1}$ at $350\,\text {GeV}$

Channel	Quantity	Precision
${{{{\upmu }}^{+} {{\upmu }}^{-}}} X$	$m_{{\mathrm{H}}}$	122 MeV
	$\sigma ({\mathrm{Z}} {\mathrm{H}})$	4.72%
${\mathrm{e}^{+}}{\mathrm{e}^{-}} X$	$m_{{\mathrm{H}}}$	278 MeV
	$\sigma ({\mathrm{Z}} {\mathrm{H}})$	7.21%
${\mathrm{e}^{+}}{\mathrm{e}^{-}} X$	$m_{{\mathrm{H}}}$	359 MeV
+ bremsstrahlung recovery	$\sigma ({\mathrm{Z}} {\mathrm{H}})$	6.60%

#### Hadronic decays: ${\mathrm{Z}} \rightarrow \mathrm{q}{\bar{\mathrm{q}}}$

In the process ${\mathrm{e}^{+}}{\mathrm{e}^{-}} \rightarrow {\mathrm{Z}} {\mathrm{H}}$, it is possible to cleanly identify ${\mathrm{Z}} \rightarrow {\mathrm{e}^{+}}{\mathrm{e}^{-}}$ and ${\mathrm{Z}} \rightarrow {{{\upmu }}^{+} {{\upmu }}^{-}}$ decays regardless of the decay mode of the Higgs boson and, consequently, the selection efficiency is almost independent of the Higgs decay mode. In contrast, for ${\mathrm{Z}} \rightarrow \mathrm{q}{\bar{\mathrm{q}}}$ decays, the selection efficiency shows a stronger dependence on the Higgs decay mode [45]. For example, ${\mathrm{e}^{+}}{\mathrm{e}^{-}} \rightarrow ({\mathrm{Z}} \rightarrow \mathrm{q}{\bar{\mathrm{q}}})({\mathrm{H}} \rightarrow {\mathrm{b}} {\bar{\mathrm{b}}})$ events consist of four jets and the reconstruction of the ${\mathrm{Z}}$ boson is complicated by ambiguities in associations of particles with jets and the three-fold ambiguity in associating four jets with the hadronic decays of the ${\mathrm{Z}}$ and ${\mathrm{H}}$. For this reason, it is much more difficult to construct a selection based only on the reconstructed ${\mathrm{Z}} \rightarrow \mathrm{q}{\bar{\mathrm{q}}}$ decay that has a selection efficiency independent of the Higgs decay mode. The strategy adopted is to first reject events consistent with a number of clear background topologies using the information from the whole event; and then to identify ${\mathrm{e}^{+}}{\mathrm{e}^{-}} \rightarrow ({\mathrm{Z}} \rightarrow \mathrm{q}{\bar{\mathrm{q}}}){\mathrm{H}}$ events solely based on the properties from the candidate ${\mathrm{Z}} \rightarrow \mathrm{q}{\bar{\mathrm{q}}}$ decay.

The $({\mathrm{Z}} \rightarrow \mathrm{q}{\bar{\mathrm{q}}}){\mathrm{H}}$ event selection proceeds in three separate stages. In the first stage, to allow for possible BSM invisible Higgs decay modes, events are divided into candidate visible Higgs decays and candidate invisible Higgs decays, in both cases produced along with a ${\mathrm{Z}} \rightarrow \mathrm{q}{\bar{\mathrm{q}}}$. Events are categorised as potential visible Higgs decays if they are not compatible with a clear two-jet topology:

- $$\log _{10}(y_{23})> -2.0$$    or    $$\log _{10}(y_{34})> -3.0$$.

All other events are considered as candidates for an invisible Higgs decay analysis, based on that described in Sect. 5.1.3, although with looser requirements to make the overall analysis more inclusive.

Preselection cuts then reduce the backgrounds from large cross section processes such as ${\mathrm{e}^{+}}{\mathrm{e}^{-}} \rightarrow \mathrm{q}{\bar{\mathrm{q}}}$ and ${\mathrm{e}^{+}}{\mathrm{e}^{-}} \rightarrow \mathrm{q}{\bar{\mathrm{q}}} \mathrm{q}{\bar{\mathrm{q}}}$. The preselection variables are formed by forcing each event into three, four and five jets. In each case, the best candidate for being a hadronically decaying ${\mathrm{Z}}$ boson is chosen as the jet pair giving the di-jet invariant mass ($m_{\mathrm{q} {\bar{\mathrm{q}}}}$) closest to $m_{{\mathrm{Z}}}$, considering only jets with more than three charged particles. The invariant mass of the system recoiling against the ${\mathrm{Z}}$ boson candidate, $m_\mathrm {rec}$, is calculated assuming $E_\text {rec} =\sqrt{s}- E_{\mathrm{q}{\bar{\mathrm{q}}}}$ and $\mathbf {p}_\text {rec} = -\mathbf {p}_{\mathrm{q}{\bar{\mathrm{q}}}}$. In addition, the invariant mass of all the visible particles not originating from the candidate ${\mathrm{Z}} \rightarrow \mathrm{q}{\bar{\mathrm{q}}}$ decay, $m_\mathrm {vis}$, is calculated. It is important to note that $m_\mathrm {vis}$ is only used to reject specific background topologies in the preselection and is not used in the main selection as it depends strongly on the type of Higgs decay. The preselection cuts are:

- $$70\,\text {GeV}< m_{\mathrm{q} {\bar{\mathrm{q}}}} < 110\,\text {GeV} $$ and $$80\,\text {GeV}< m_\mathrm {rec} < 200\,\text {GeV} $$;
- the background from ${\mathrm{e}^{+}}{\mathrm{e}^{-}} \rightarrow \mathrm{q}{\bar{\mathrm{q}}}$ is suppressed by removing events with overall $p_\mathrm {T} < 20\,\text {GeV}$ and either $|\cos \theta _\text {mis}|>0.90$ or $\log _{10}(y_{34})>-2.5$, where $\theta _\text {mis}$ is the polar angle of the missing momentum vector;
- events with little missing transverse momentum ($p_\mathrm {T} < 20\,\text {GeV}$) are forced into four jets and are rejected if the reconstructed di-jet invariant masses (and particle types) are consistent with the expectations for ${\mathrm{e}^{+}}{\mathrm{e}^{-}} \rightarrow \mathrm{q}{\bar{\mathrm{q}}} \mathrm{l} \mathrm{l}$, ${\mathrm{e}^{+}}{\mathrm{e}^{-}} \rightarrow {\mathrm{Z}} {\mathrm{Z}} \rightarrow \mathrm{q}{\bar{\mathrm{q}}} \mathrm{q}{\bar{\mathrm{q}}}$, ${\mathrm{e}^{+}}{\mathrm{e}^{-}} \rightarrow {\mathrm{W}} {\mathrm{W}} \rightarrow \mathrm{q}{\bar{\mathrm{q}}} \mathrm{q}{\bar{\mathrm{q}}}$.

The final step in the event selection is a multivariate analysis. In order not to bias the event selection efficiencies for different Higgs decay modes, only variables related to the candidate ${\mathrm{Z}} \rightarrow \mathrm{q}{\bar{\mathrm{q}}}$ decay are used in the selection. Forcing the event into four jets is the right approach for $({\mathrm{Z}} \rightarrow \mathrm{q}{\bar{\mathrm{q}}}){\mathrm{H}}$ events where the Higgs decays to two-body final states, but not necessarily for final states such as ${\mathrm{H}} \rightarrow {\mathrm{W}} {\mathrm{W}} ^*\rightarrow \mathrm{q}{\bar{\mathrm{q}}} \mathrm{q}{\bar{\mathrm{q}}}$, where there is the chance that one of the jets from the ${\mathrm{W}} {\mathrm{W}} ^*$ decay will be merged with one of the jets from the ${\mathrm{Z}} \rightarrow \mathrm{q}{\bar{\mathrm{q}}}$, potentially biasing the selection against ${\mathrm{H}} \rightarrow {\mathrm{W}} {\mathrm{W}} ^*$ decays. To mitigate this effect, the ${\mathrm{Z}}$ candidate for the event selection can either be formed from the four-jet topology as described above, or can be formed from a jet pair after forcing the event into a five-jet topology. The latter case is only used when $\log _{10}(y_{45}) > -3.5$ and the five-jet reconstruction gives better ${\mathrm{Z}}$ and ${\mathrm{H}}$ candidates than the four-jet reconstruction. Attempting to reconstruct events in the six-jet topology is not found to improve the overall analyses. Having chosen the best ${\mathrm{Z}}$ candidate in the event (from either the four-jet or five-jet reconstruction), it is used to form variables for the multivariate selection; information about the remainder of the event is not used.

**Fig. 9:**
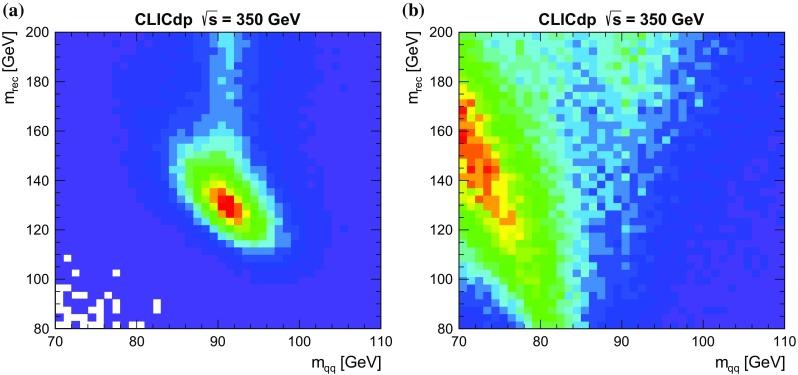
Reconstructed di-jet invariant mass versus reconstructed recoil mass distributions for ${\mathrm{Z}} {\mathrm{H}} \rightarrow \mathrm{q}{\bar{\mathrm{q}}} X$ candidate events at $\sqrt{s} =350\,\text {GeV}$, showing ${\mathrm{Z}} {\mathrm{H}}$ signal events (**a**) and all background processes (**b**). In both cases the plots show all events passing the preselection

A relative likelihood selection is used to classify all events passing the preselection cuts. Two event categories are considered: the ${\mathrm{e}^{+}}{\mathrm{e}^{-}} \rightarrow {\mathrm{Z}} {\mathrm{H}} \rightarrow \mathrm{q}{\bar{\mathrm{q}}} {\mathrm{H}}$ signal and all non-Higgs background processes. The relative likelihood for an event being signal is defined as:

$$\begin{aligned} \mathcal{{L}} = \frac{L_\text {signal} }{L_\text {signal} + L_\text {back}}, \end{aligned}$$

where the individual absolute likelihood *L* for each event type is estimated from normalised probability distributions, $P_i(x_i)$, of the discriminating variables $x_i$ for that event type:

$$\begin{aligned} L = \sigma _\text {presel} \times \prod _i^{N} P_i(x_i) , \end{aligned}$$

where $\sigma _\text {presel}$ is the cross section after the preselection cuts. The discriminating variables used, all of which are based on the candidate ${\mathrm{Z}} \rightarrow \mathrm{q}{\bar{\mathrm{q}}}$ decay, are: the 2D distribution of $m_{\mathrm{q} {\bar{\mathrm{q}}}}$ and $m_\mathrm {rec}$; the polar angle of the ${\mathrm{Z}}$ candidate, $|\cos \theta _{{\mathrm{Z}}}|$; and the modulus of angle of jets from the ${\mathrm{Z}}$ decay relative to its direction after boosting into its rest frame, $|\cos \theta _{\mathrm{q}}|$. The clearest separation between signal and background is obtained from $m_{\mathrm{q} {\bar{\mathrm{q}}}}$ and the recoil mass $m_\mathrm {rec}$, as shown in Fig. 9 for events passing the preselection. The signal is clearly peaked at $m_{\mathrm{q} {\bar{\mathrm{q}}}} \approx m_{{\mathrm{Z}}}$ and $m_\mathrm {rec} \approx m_{{\mathrm{H}}}$. The use of 2D mass distributions accounts for the most significant correlations between the likelihood variables.

In this high-statistics limit, the fractional error on the number of signal events (where the Higgs decays to visible final states), $s_\text {vis}$, given a background *b* is:

$$\begin{aligned} \frac{\varDelta s_\text {vis}}{s_\text {vis}} = \frac{\sqrt{s_\text {vis}+b}}{s_\text {vis}}, \end{aligned}$$

and this is minimised with the selection requirement $\mathcal{{L}}>0.65$. The selection efficiencies and expected numbers of events for the signal dominated region, $\mathcal{{L}}>0.65$, are listed in Table 6, corresponding to a fractional error on the number of signal events of $1.9\%$. By fitting the shape of the likelihood distribution to signal and background contributions, this uncertainty is reduced to:

$$\begin{aligned} \frac{\varDelta s_\text {vis} }{s_\text {vis}} = 1.7\% . \end{aligned}$$

This is an example of a measurement for which it will be particularly important to tune the background modelling using high-statistics processes.

**Table 6:** Summary of the $({\mathrm{Z}} \rightarrow \mathrm{q}{\bar{\mathrm{q}}})({\mathrm{H}} \rightarrow \text {vis.})$ event selection at $\sqrt{s} =350\,\text {GeV}$, giving the raw cross sections, preselection efficiency, overall selection efficiency for a likelihood cut of $\mathcal{{L}}>0.65$ and the expected numbers of events passing the event selection for an integrated luminosity of $500\,\text {fb}^{-1}$

Process	$\sigma /\text {fb}$	$\varepsilon _\text {presel}$ (%)	$\varepsilon _{\mathcal{{L}}>0.65}$ (%)	$N_{\mathcal{{L}}>0.65}$
$\mathrm{q} {\bar{\mathrm{q}}}$	25,200	0.4	17	8525
$\mathrm{q} {\bar{\mathrm{q}}} \mathrm{l} {\nu }$	5910	11	1.7	5767
$\mathrm{q} {\bar{\mathrm{q}}} \mathrm{q} {\bar{\mathrm{q}}}$	5850	3.8	13	14,142
$\mathrm{q} {\bar{\mathrm{q}}} \mathrm{l} \mathrm{l}$	1700	1.5	15	1961
$\mathrm{q} {\bar{\mathrm{q}}} {\nu } \bar{\nu }$	325	0.6	6.2	60
${\mathrm{H}} {{\nu }}_{\!\mathrm{e}} {\bar{{\nu }}}_{\!\mathrm{e}}$	52	2.5	9.2	60
${\mathrm{Z}} {\mathrm{H}}$; ${\mathrm{Z}} \rightarrow \mathrm{q} {\bar{\mathrm{q}}}$	93	42.0	54	10,568

#### Invisible Higgs decays

The above recoil mass analysis of leptonic decays of the ${\mathrm{Z}}$ boson in ${\mathrm{e}^{+}}{\mathrm{e}^{-}} \rightarrow {\mathrm{Z}} {\mathrm{H}}$ events provides a measurement of the Higgsstrahlung cross section, independent of the Higgs boson decay model. The recoil mass technique can also be used to search for BSM decay modes of the Higgs boson into long-lived neutral “invisible” final states [45]. At an ${\mathrm{e}^{+}}{\mathrm{e}^{-}}$ collider, a search for invisible Higgs decays is possible by identification of ${\mathrm{e}^{+}}{\mathrm{e}^{-}} \rightarrow {\mathrm{Z}} {\mathrm{H}}$ events with a visible ${\mathrm{Z}} \rightarrow \mathrm{q}{\bar{\mathrm{q}}}$ decay and missing energy. Such events would typically produce a clear two-jet topology with invariant mass consistent with $m_{{\mathrm{Z}}}$, significant missing energy and a recoil mass corresponding to the Higgs mass. Higgsstrahlung events with leptonic ${\mathrm{Z}}$ decays, which have a much smaller branching ratio, are not included in the current analysis.

**Fig. 10:**
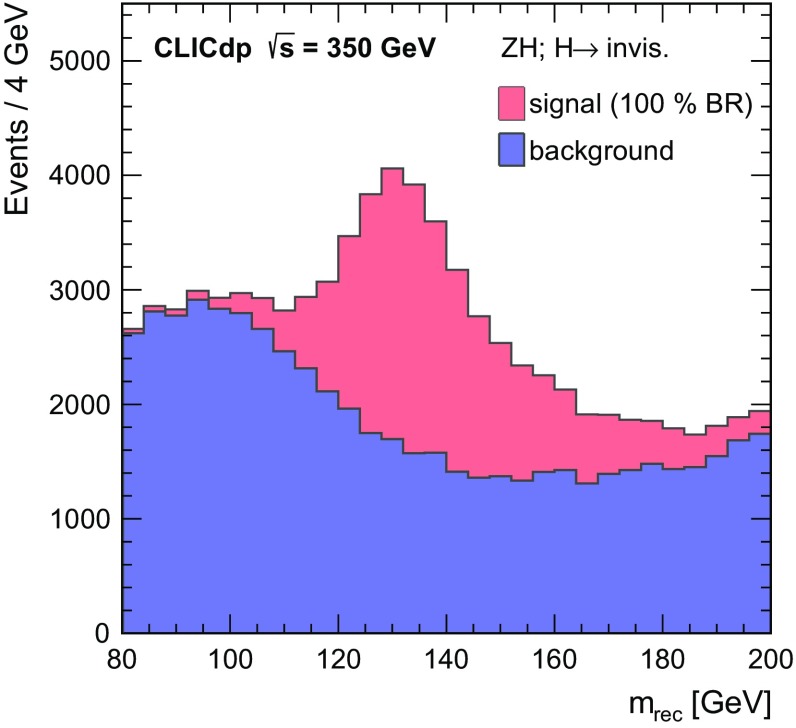
Reconstructed recoil mass distributions of ${\mathrm{e}^{+}}{\mathrm{e}^{-}} \rightarrow {\mathrm{Z}} {\mathrm{H}}$ events at $\sqrt{s} =350\,\text {GeV}$, showing the ${\mathrm{H}} \rightarrow \text {invis.}$ signal, assuming $BR ({\mathrm{H}} \rightarrow \text {invis.}) = 100\%$, and SM backgrounds as stacked histograms. The distributions are normalised to an integrated luminosity of $500\,\text {fb}^{-1}$

To identify candidate invisible Higgs decays, a loose preselection is imposed requiring: (i) a clear two-jet topology, defined by $\log _{10}(y_{23}) < -2.0$ and $\log _{10}(y_{34}) < -3.0$, using the minimal $k_t$ distances discussed in Sect. 4.2; (ii) a di-jet invariant mass consistent with $m_{{\mathrm{Z}}}$, $84\,\text {GeV}< m_{\mathrm{q} {\bar{\mathrm{q}}}} < 104\,\text {GeV}$; and (iii) the reconstructed momentum of the candidate ${\mathrm{Z}}$ boson pointing away from the beam direction, $|\cos \theta _{{\mathrm{Z}}}| < 0.7$. After the preselection, a BDT multivariate analysis technique is applied using the Tmva package [47] to further separate the invisible Higgs signal from the SM background. In addition to $m_{\mathrm{q} {\bar{\mathrm{q}}}}$, $|\cos \theta _{{\mathrm{Z}}}|$ and $\log _{10}(y_{23})$, four other discriminating variables are employed: $m_\mathrm {rec}$, the recoil mass of the invisible system recoiling against the observed ${\mathrm{Z}}$ boson; $|\cos \theta _{\mathrm{q}}|$, the decay angle of one of the quarks in the ${\mathrm{Z}}$ rest frame, relative to the direction of flight of the ${\mathrm{Z}}$ boson; $p_\mathrm {T}$, the magnitude of the transverse momentum of the Z boson; and $E_\mathrm {vis}$, the visible energy in the event. As an example, Fig. 10 shows the recoil mass distribution for the simulated invisible Higgs decays and the total SM background. The reconstructed recoil mass for events with invisible Higgs decays peaks near $m_{{\mathrm{H}}}$. The cut applied on the BDT output is chosen to minimise the statistical uncertainty on the cross section for invisible Higgs decays.

In the case where the branching ratio to BSM invisible final states is zero (or very small), the uncertainty on the invisible branching ratio is determined by the statistical fluctuations on the background after the event selection:

$$\begin{aligned} \varDelta BR ({\mathrm{H}} \rightarrow \text {invis.}) = \frac{\sqrt{b} }{s(100\%)} , \end{aligned}$$

where *b* is the expected number of selected SM background events and $s(100\%)$ is the expected number of selected Higgsstrahlung events assuming all Higgs bosons decay invisibly, i.e. $BR ({\mathrm{H}} \rightarrow \text {invis.}) = 100\%$. Table 7 summarises the invisible Higgs decay event selection; the dominant background processes arise from the final states $\mathrm{q}{\bar{\mathrm{q}}} \mathrm{l} {\nu }$ and $\mathrm{q}{\bar{\mathrm{q}}} \nu \bar{\nu }$. The resulting one sigma uncertainty on $BR ({\mathrm{H}} \rightarrow \text {invis.})$ is 0.57% (in the case where the invisible Higgs branching ratio is small) and the corresponding 90% C.L. upper limit (500 $\text {fb}^{-1}$ at $\sqrt{s}$ =350 GeV) on the invisible Higgs branching ratio in the modified frequentist approach [51] is:

$$\begin{aligned} BR ({\mathrm{H}} \rightarrow \text {invis.}) < 0.97\% \quad \text{ at } \ 90\% \ \text {C.L.} \end{aligned}$$

It should be noted that the SM Higgs decay chain ${\mathrm{H}} \rightarrow {\mathrm{Z}} {\mathrm{Z}} ^*\rightarrow \nu \bar{\nu }\nu \bar{\nu }$ has a combined branching ratio of 0.1% and is not measurable.

**Table 7:** Summary of the invisible Higgs decay event selection at $\sqrt{s} =350\,\text {GeV}$, giving the raw cross sections, preselection efficiency, selection efficiency for a BDT cut of $\text {BDT}>0.088$, and the expected numbers of events passing the event selection for an integrated luminosity of $500\,\text {fb}^{-1}$. For the invisible Higgs decay signal the number of selected events corresponds to a $BR$ of 100%. Contributions from all other backgrounds are found to be negligibly small

Process	$\sigma /\text {fb}$	$\varepsilon _\text {presel}$ (%)	$\varepsilon _{\text {BDT}>0.088}$ (%)	$N_{\text {BDT}>0.088}$
$\mathrm{q}{\bar{\mathrm{q}}} \mathrm{l} {\nu }$	5910	0.68	4.5	900
$\mathrm{q} {\bar{\mathrm{q}}} {\nu } \bar{\nu }$	325	17	8.9	2414
${\mathrm{Z}} {\mathrm{H}}$ (SM decays)	93.4	0.2	23	21
${\mathrm{H}} \rightarrow \text {invis.}$		41	51	9956

#### Model-independent ${\mathrm{Z}} {\mathrm{H}}$ cross section

By combining the two analyses for ${\mathrm{Z}} {\mathrm{H}}$ production where ${\mathrm{Z}} \rightarrow \mathrm{q}{\bar{\mathrm{q}}}$ and the Higgs decays either to invisible final states (see Sect. 5.1.3) or to visible final states (see Sect. 5.1.2), it is possible to determine the absolute cross section for ${\mathrm{e}^{+}}{\mathrm{e}^{-}}$ $\rightarrow {\mathrm{Z}} {\mathrm{H}}$ in an essentially model-independent manner:

$$\begin{aligned} \sigma ({\mathrm{Z}} {\mathrm{H}}) = \frac{\sigma _\text {vis} + \sigma _\text {invis}}{BR ({\mathrm{Z}} \rightarrow \mathrm{q}{\bar{\mathrm{q}}})}. \end{aligned}$$

Here a slightly modified version of the invisible Higgs analysis is employed. With the exception of the cuts on $y_{23}$ and $y_{34}$, the invisible Higgs analysis employs the same preselection as for the visible Higgs analysis and a likelihood multivariate discriminant is used.

Since the fractional uncertainties on the total cross section from the visible and invisible cross sections are 1.7 and $0.6\%$ respectively, the fractional uncertainty on the total cross section will be (at most) the quadrature sum of the two fractional uncertainties, namely $1.8\%$. This measurement is only truly model-independent if the overall selection efficiencies are independent of the Higgs decay mode. For all final state topologies, the combined (visible + invisible) selection efficiency lies is the range 19–26% regardless of the Higgs decay mode, covering a very wide range of event topologies. To assess the level of model independence, the Higgs decay modes in the MC samples are modified and the total (visible + invisible) cross section is extracted assuming the SM Higgs branching ratio. Table 8 shows the resulting biases in the extracted total cross section for the case when a $BR ({\mathrm{H}} \rightarrow X) \rightarrow BR ({\mathrm{H}} \rightarrow X) + 0.05$. Even for these very large modifications of the Higgs branching ratios over a wide range of final-state topologies – including the extreme cases highlighted at the bottom of Table 8 such as ${\mathrm{H}} \rightarrow {\mathrm{W}} {\mathrm{W}} ^*\rightarrow \mathrm{q}{\bar{\mathrm{q}}} \mathrm{q}{\bar{\mathrm{q}}}$, which has six jets in the final state, and ${\mathrm{H}} \rightarrow {\mathrm{W}} {\mathrm{W}} ^*\rightarrow \uptau {\nu } \uptau {\nu }$, which has a lot of missing energy – the resulting biases in the extracted total ${\mathrm{Z}} {\mathrm{H}}$ cross section are less than $1\%$ (compared to the $1.8\%$ statistical uncertainty). However, such large deviations would have significant observable effects on exclusive Higgs branching ratio analyses (at both LHC and CLIC) and it is concluded that the analysis gives an effectively model-independent measurement of the $({\mathrm{Z}} \rightarrow \mathrm{q}{\bar{\mathrm{q}}}){\mathrm{H}}$ cross section.

**Table 8:** Biases in the extracted ${\mathrm{H}} ({\mathrm{Z}} \rightarrow \mathrm{q}{\bar{\mathrm{q}}})$ cross section if the Higgs branching ratio to a specific final state is increased by 5%, i.e. $BR ({\mathrm{H}} \rightarrow X) \rightarrow BR ({\mathrm{H}} \rightarrow X) + 0.05$

Decay mode	$\varDelta$ ($BR$) (%)	$\sigma ^{\text {vis}} + \sigma ^{\text {invis}}$ Bias (%)
${\mathrm{H}} \rightarrow \text {invis}$	$+5$	$-0.01$
${\mathrm{H}} \rightarrow \mathrm{q}{\bar{\mathrm{q}}}$	$+5$	$+0.05$
${\mathrm{H}} \rightarrow {\mathrm{W}} {\mathrm{W}} ^*$	$+5$	$-0.18$
${\mathrm{H}} \rightarrow {\mathrm{Z}} {\mathrm{Z}} ^*$	$+5$	$-0.30$
${\mathrm{H}} \rightarrow \uptau ^{+} \uptau ^{-}$	$+5$	$+0.60$
${\mathrm{H}} \rightarrow {\upgamma } {\upgamma }$	$+5$	$+0.79$
${\mathrm{H}} \rightarrow {\mathrm{Z}} {\upgamma }$	$+5$	$-0.74$
${\mathrm{H}} \rightarrow {\mathrm{W}} {\mathrm{W}} ^*\rightarrow \mathrm{q}{\bar{\mathrm{q}}} \mathrm{q}{\bar{\mathrm{q}}}$	$+5$	$-0.49$
${\mathrm{H}} \rightarrow {\mathrm{W}} {\mathrm{W}} ^*\rightarrow \mathrm{q}{\bar{\mathrm{q}}} \mathrm{l} {\nu }$	$+5$	$+0.10$
${\mathrm{H}} \rightarrow {\mathrm{W}} {\mathrm{W}} ^*\rightarrow \uptau {\nu } \uptau {\nu }$	$+5$	$-0.98$

Combining the model-independent measurements of the ${\mathrm{Z}} {\mathrm{H}}$ cross section from ${\mathrm{Z}} \rightarrow {\mathrm{l}}^{+} {\mathrm{l}}^{-}$ and ${\mathrm{Z}} \rightarrow \mathrm{q}{\bar{\mathrm{q}}}$ gives an absolute measurement of the ${\mathrm{Z}} {\mathrm{H}}$ cross section with a precision of:

$$\begin{aligned} \frac{\varDelta \sigma ({{\mathrm{Z}} {\mathrm{H}}})}{\sigma ({\mathrm{Z}} {\mathrm{H}})} = 1.65\% , \end{aligned}$$

and, consequently, the absolute coupling of the ${\mathrm{H}}$ boson to the ${\mathrm{Z}}$ boson is determined to:

$$\begin{aligned} \frac{\varDelta g_{{\mathrm{H}} {\mathrm{Z}} {\mathrm{Z}}}}{g_{{\mathrm{H}} {\mathrm{Z}} {\mathrm{Z}}}} = 0.8\% . \end{aligned}$$

The hadronic recoil mass analysis was repeated for collision energies of $\sqrt{s} =250\,\text {GeV}$ and $\sqrt{s} =420\,\text {GeV}$ [45]. Compared with $\sqrt{s} =350\,\text {GeV}$, the sensitivity is significantly worse in both cases.

### Exclusive Higgs branching ratio measurements at $\sqrt{s} =350\,\text {GeV}$

The previous section described inclusive measurements of the ${\mathrm{e}^{+}}{\mathrm{e}^{-}} \rightarrow {\mathrm{Z}} {\mathrm{H}}$ production cross section, which provide a model-independent determination of the coupling at the ${\mathrm{H}} {\mathrm{Z}} {\mathrm{Z}}$ vertex. In contrast, measurements of Higgs production and decay to exclusive final states provide a determination of the product $\sigma ({\mathrm{Z}} {\mathrm{H}}) \times BR ({\mathrm{H}} \rightarrow X)$, where *X* is a particular final state. This section focuses on the exclusive measurements of the Higgs decay branching ratios at $\sqrt{s} =350\,\text {GeV}$. Higgs boson decays to ${\mathrm{b}} {\bar{\mathrm{b}}}$, ${\mathrm{c}} {\bar{{\mathrm{c}}}}$ and ${\mathrm{g}} {\mathrm{g}}$ are studied in Sect. 5.2.1. The measurement of ${\mathrm{H}} \rightarrow \uptau ^{+} \uptau ^{-}$ decays is described in Sect. 5.2.2, and the ${\mathrm{H}} \rightarrow {\mathrm{W}} {\mathrm{W}} ^*$ decay mode is described in Sect. 5.2.3.

#### ${\mathrm{H}} \rightarrow {\mathrm{b}} {\bar{\mathrm{b}}},~{\mathrm{c}} {\bar{{\mathrm{c}}}}$ and ${\mathrm{g}} {\mathrm{g}}$

As can be seen from Table 1, at $\sqrt{s} =350\,\text {GeV}$ the cross section for ${\mathrm{e}^{+}}{\mathrm{e}^{-}} \rightarrow {\mathrm{Z}} {\mathrm{H}}$ (Higgsstrahlung) is approximately four times greater than the ${\mathrm{e}^{+}}{\mathrm{e}^{-}} \rightarrow {\mathrm{H}} {{\nu }}_{\!\mathrm{e}} {\bar{{\nu }}}_{\!\mathrm{e}}$ (mostly ${\mathrm{W}} {\mathrm{W}}$-fusion) cross section for unpolarised beams (or approximately a factor 2.5 with $-80\%$ electron beam polarisation). For Higgsstrahlung, the signature of ${\mathrm{H}} \rightarrow {\mathrm{b}} {\bar{\mathrm{b}}} , {\mathrm{c}} {\bar{{\mathrm{c}}}} , {\mathrm{g}} {\mathrm{g}}$ events depends on the ${\mathrm{Z}}$ decay mode.

**Table 9:** Summary of the expected numbers of events for the different Higgs and non-Higgs final states passing the hadronic Higgs decay signal selection for $500\,\text {fb}^{-1}$ at $\sqrt{s} =350\,\text {GeV}$ (unpolarised beams). No preselection is applied in this analysis

Process	$\sigma$/fb	$\epsilon _\text {BDT}$, classified as		$N_\text {BDT}$, classified as	
		${\mathrm{H}} \nu \bar{\nu }$ (%)	${\mathrm{H}} \mathrm{q}{\bar{\mathrm{q}}}$ (%)	${\mathrm{H}} \nu \bar{\nu }$	${\mathrm{H}} \mathrm{q}{\bar{\mathrm{q}}}$
${\mathrm{e}^{+}}{\mathrm{e}^{-}} \rightarrow {\mathrm{H}} \nu \bar{\nu }; {\mathrm{H}} \rightarrow {\mathrm{b}} {\bar{\mathrm{b}}}$	28.9	55	0	8000	0
${\mathrm{e}^{+}}{\mathrm{e}^{-}} \rightarrow {\mathrm{H}} \nu \bar{\nu }; {\mathrm{H}} \rightarrow {\mathrm{c}} {\bar{{\mathrm{c}}}}$	1.46	51	0	372	0
${\mathrm{e}^{+}}{\mathrm{e}^{-}} \rightarrow {\mathrm{H}} \nu \bar{\nu }; {\mathrm{H}} \rightarrow {\mathrm{g}} {\mathrm{g}}$	4.37	58	0	1270	0
${\mathrm{e}^{+}}{\mathrm{e}^{-}} \rightarrow {\mathrm{H}} \nu \bar{\nu }; {\mathrm{H}} \rightarrow \text {other}$	16.8	6.1	0	513	0
${\mathrm{e}^{+}}{\mathrm{e}^{-}} \rightarrow {\mathrm{H}} \mathrm{q}{\bar{\mathrm{q}}}; {\mathrm{H}} \rightarrow {\mathrm{b}} {\bar{\mathrm{b}}}$	52.3	0	42	0	11,100
${\mathrm{e}^{+}}{\mathrm{e}^{-}} \rightarrow {\mathrm{H}} \mathrm{q}{\bar{\mathrm{q}}}; {\mathrm{H}} \rightarrow {\mathrm{c}} {\bar{{\mathrm{c}}}}$	2.64	0	33	0	434
${\mathrm{e}^{+}}{\mathrm{e}^{-}} \rightarrow {\mathrm{H}} \mathrm{q}{\bar{\mathrm{q}}}; {\mathrm{H}} \rightarrow {\mathrm{g}} {\mathrm{g}}$	7.92	0	37	0	1480
${\mathrm{e}^{+}}{\mathrm{e}^{-}} \rightarrow {\mathrm{H}} \mathrm{q}{\bar{\mathrm{q}}}; {\mathrm{H}} \rightarrow \text {other}$	30.5	0.12	13	20	1920
${\mathrm{e}^{+}}{\mathrm{e}^{-}} \rightarrow \mathrm{q}{\bar{\mathrm{q}}} \nu \bar{\nu }$	325	1.3	0	2110	0
${\mathrm{e}^{+}}{\mathrm{e}^{-}} \rightarrow \mathrm{q}{\bar{\mathrm{q}}} \mathrm{l} {\nu }$	5910	0.07	0.002	2090	60
${\mathrm{e}^{+}}{\mathrm{e}^{-}} \rightarrow \mathrm{q}{\bar{\mathrm{q}}} \mathrm{l} \mathrm{l}$	1700	0.012	0.01	104	89
${\mathrm{e}^{+}}{\mathrm{e}^{-}} \rightarrow \mathrm{q}{\bar{\mathrm{q}}} \mathrm{q}{\bar{\mathrm{q}}}$	5530	0.001	0.36	30	9990
${\mathrm{e}^{+}}{\mathrm{e}^{-}} \rightarrow \mathrm{q}{\bar{\mathrm{q}}}$	24,400	0.01	0.093	1230	11,400

To maximise the statistical power of the ${\mathrm{H}} \rightarrow {\mathrm{b}} {\bar{\mathrm{b}}} , {\mathrm{c}} {\bar{{\mathrm{c}}}} , {\mathrm{g}} {\mathrm{g}}$ branching ratio measurements, two topologies are considered: four jets, and two jets plus missing momentum (from the unobserved neutrinos). The impact of Higgsstrahlung events with leptonic ${\mathrm{Z}}$ decays is found to be negligible. The jets plus missing momentum final state contains approximately equal contributions from Higgsstrahlung and ${\mathrm{W}} {\mathrm{W}}$-fusion events, although the event kinematics are very different. All events are initially reconstructed assuming both topologies; at a later stage of the event selection, events are assigned to either ${\mathrm{H}} \mathrm{q}{\bar{\mathrm{q}}}$, ${\mathrm{H}} \nu \bar{\nu }$, or background. To minimize the impact of ISR on the jet reconstruction, photons with a reconstructed energy higher than $15\,\text {GeV}$ are removed from the events first.

The hadronic final states are reconstructed using the Durham algorithm. For the four-jet topology, the most probable Z and Higgs boson candidates are selected by choosing the jet combination that minimises:

$$\begin{aligned} \chi ^2 = (m_{ij}-m_{{\mathrm{H}}})^2/\sigma ^2_{{\mathrm{H}}} + (m_{kl}-m_{{\mathrm{Z}}})^2/\sigma ^2_{{\mathrm{Z}}} , \end{aligned}$$

where $m_{ij}$ and $m_{kl}$ are the invariant masses of the jet pairs used to reconstruct the Higgs and ${\mathrm{Z}}$ boson candidates, respectively, and $\sigma _{{\mathrm{H}}, {\mathrm{Z}}}$ are the estimated invariant mass resolutions for Higgs and Z boson candidates. In the case of the two jets plus missing energy final state, either from ${\mathrm{Z}} {\mathrm{H}}$ with ${\mathrm{Z}} \rightarrow \nu \bar{\nu }$ or from ${\mathrm{H}} \nu \bar{\nu }$, the event is clustered into two jets forming the H candidate.

To help veto backgrounds with leptonic final states, isolated electrons or muons with $E > 10\,\text {GeV}$ are identified with the additional requirement that there should be less than $20\,\text {GeV}$ of energy from other particles within a cone with an opening angle of $20^\circ$ around the lepton direction. All events are then classified by gradient boost decision trees employing reconstructed kinematic variables from each of the two event topology hypotheses described above. The variables used include jet energies, event shape variables (such as thrust and sphericity), the masses of H and Z candidates, their decay angles and transverse momenta, and the number of isolated leptons in the final state. The total number of variables is about 50, which is larger than in other studies presented in this paper, because each event is reconstructed assuming two different final state configurations and information from the ${\mathrm{H}}$ candidate decay can be included here, in contrast with the recoil mass analyses described in Sect. 5.1.

Two separate BDT classifiers are used, one for each signal final state (${\mathrm{H}} \mathrm{q}{\bar{\mathrm{q}}}$ and ${\mathrm{H}} \nu \bar{\nu }$), irrespective of the nature of the hadronic Higgs decay mode. Two-fermion ($\mathrm{q}{\bar{\mathrm{q}}}$) and four-fermion ($\mathrm{q}{\bar{\mathrm{q}}} \nu \bar{\nu }$, $\mathrm{q}{\bar{\mathrm{q}}} \mathrm{l} {\nu }$, $\mathrm{q}{\bar{\mathrm{q}}} \mathrm{l} \mathrm{l}$ and $\mathrm{q}{\bar{\mathrm{q}}} \mathrm{q}{\bar{\mathrm{q}}}$) final states and other Higgs decay modes are taken as background for both classifiers. In addition, the other signal mode is included in the background for a given classifier. The training is performed using a dedicated training sample, simultaneously training both classifiers. At this point, no flavour tagging information is used.

Each event is evaluated with both classifiers. An event is only accepted if exactly one of the signal classifiers is above a positive threshold and the other classifier is below a corresponding negative threshold. The event is then tagged as a candidate for the corresponding signal process. If none of the classifiers passes the selection threshold, the event is considered as background and is rejected from the analysis. The number of events for which both signal classifiers are above the positive threshold is negligible. Table 9 summarises the classification of all events into the two signal categories, with event numbers based on an integrated luminosity of $500\,\text {fb}^{-1}$.

The second stage of the analysis is to measure the contributions of the hadronic Higgs decays into the ${\mathrm{H}} \rightarrow {\mathrm{b}} {\bar{\mathrm{b}}}$, ${\mathrm{H}} \rightarrow {\mathrm{c}} {\bar{{\mathrm{c}}}}$ and ${\mathrm{H}} \rightarrow {\mathrm{g}} {\mathrm{g}}$ exclusive final states, separated into the two production modes Higgsstrahlung and ${\mathrm{W}} {\mathrm{W}}$-fusion. This is achieved by a multi-dimensional template fit using flavour tagging information and, in the case of the ${\mathrm{H}} \nu \bar{\nu }$ final state, the transverse momentum of the Higgs candidate.

**Fig. 11:**
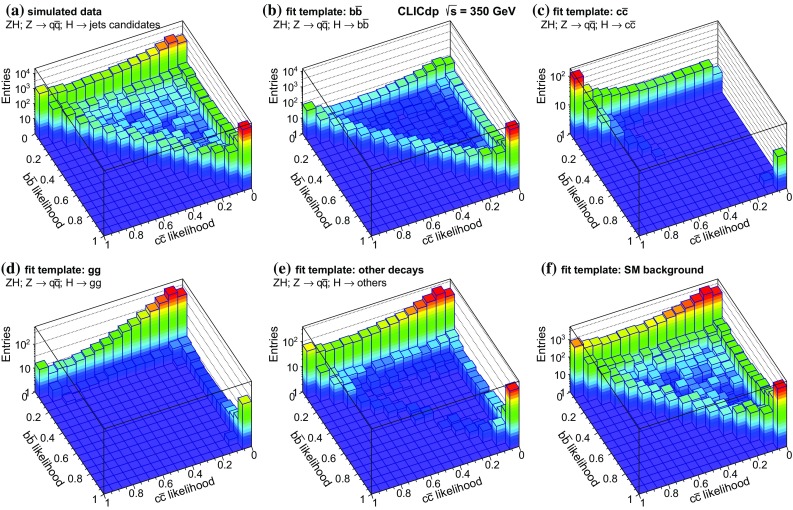
${\mathrm{b}} {\bar{\mathrm{b}}}$ likelihood versus ${\mathrm{c}} {\bar{{\mathrm{c}}}}$ likelihood distributions for ${\mathrm{e}^{+}}{\mathrm{e}^{-}} \rightarrow {\mathrm{Z}} {\mathrm{H}}$ events at $\sqrt{s} =350\,\text {GeV}$, for (**a**) all events and for the different event classes: (**b**) H$\rightarrow$b$\bar{\mathrm{b}}$, (**c**) H$\rightarrow$c$\bar{\mathrm{c}}$, (**d**) H$\rightarrow$gg, background from (**e**) other Higgs decays and (**f**) non-Higgs SM background. All distributions are normalised to an integrated luminosity of $500\,\text {fb}^{-1}$

The jets forming the Higgs candidate are classified with the LcfiPlus flavour tagging package. Each jet pair is assigned a ${\mathrm{b}} {\bar{\mathrm{b}}}$ likelihood and a ${\mathrm{c}} {\bar{{\mathrm{c}}}}$ likelihood:

$$\begin{aligned} {\mathrm{b}} {\bar{\mathrm{b}}} ~\text {likelihood} = \frac{b_1 b_2}{b_1 b_2 + (1 - b_1) (1 - b_2)}, \end{aligned}$$

$$\begin{aligned} {\mathrm{c}} {\bar{{\mathrm{c}}}} ~\text {likelihood} = \frac{c_1 c_2}{c_1 c_2 + (1 - c_1) (1 - c_2)}, \end{aligned}$$

where $b_1$ and $b_2$ ($c_1$ and $c_2$) are the b-tag (c-tag) values obtained for the two jets forming the Higgs candidate.

The resulting two-dimensional distributions of the ${\mathrm{b}} {\bar{\mathrm{b}}}$ and ${\mathrm{c}} {\bar{{\mathrm{c}}}}$ likelihoods in ${\mathrm{H}} \mathrm{q}{\bar{\mathrm{q}}}$ events are shown in Fig. 11, where separation between the different event categories can be seen. These distributions form the templates used to determine the contribution of the different signal categories for the ${\mathrm{H}} \mathrm{q}{\bar{\mathrm{q}}}$ final states.

**Fig. 12:**
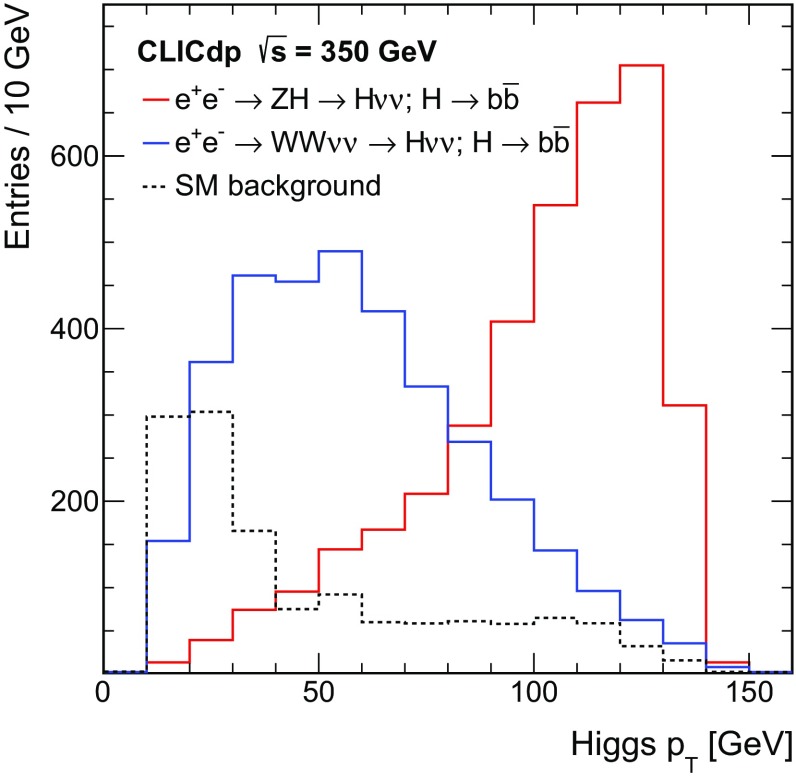
Reconstructed Higgs candidate transverse momentum distributions for selected ${\mathrm{H}} \nu \bar{\nu }$ events at $\sqrt{s} =350\,\text {GeV}$, showing the contributions from Higgsstrahlung, ${\mathrm{W}} {\mathrm{W}}$-fusion and non-Higgs background. The distributions are normalised to an integrated luminosity of $500\,\text {fb}^{-1}$

Signal and background templates are also obtained for the ${\mathrm{H}} \nu \bar{\nu }$ final state. As ${\mathrm{H}} \nu \bar{\nu }$ has roughly equal contributions from the Higgsstrahlung and the ${\mathrm{W}} {\mathrm{W}}$-fusion process, separation into the two production processes is required, in addition to separation into the different signal and background final states. This is achieved by adding the transverse momentum of the Higgs candidate to the templates as a third dimension. This exploits the fact that the transverse momentum of the Higgs candidate is substantially different for Higgsstrahlung and ${\mathrm{W}} {\mathrm{W}}$-fusion events, as illustrated in Fig. 12 for events with a high ${\mathrm{b}} {\bar{\mathrm{b}}}$ likelihood, which provides a high signal purity.

Contributions from events with ${\mathrm{H}} \rightarrow {\mathrm{b}} {\bar{\mathrm{b}}}$, ${\mathrm{H}} \rightarrow {\mathrm{c}} {\bar{{\mathrm{c}}}}$ and ${\mathrm{H}} \rightarrow {\mathrm{g}} {\mathrm{g}}$ decays, separated by production mode, are extracted in a template fit maximizing the combined likelihood of the ${\mathrm{H}} \mathrm{q}{\bar{\mathrm{q}}}$ and ${\mathrm{H}} \nu \bar{\nu }$ templates. It is assumed that the contributions from other Higgs decay modes are determined from independent measurements and therefore these contributions are fixed in the fit.

The results of the above analysis are summarised in Table 10, giving the statistical uncertainties of the various $\sigma \times BR$ measurements. Since the parameters in this analysis are determined in a combined extraction from overlapping distributions, the results are correlated. In particular the Higgsstrahlung and ${\mathrm{W}} {\mathrm{W}}$-fusion results for the same final states show sizeable anti-correlations, as large as $-38\%$ for the cases of ${\mathrm{H}} \rightarrow {\mathrm{c}} {\bar{{\mathrm{c}}}}$ and ${\mathrm{H}} \rightarrow {\mathrm{g}} {\mathrm{g}}$. These correlations are taken into account in the global fits described in Sect. 12.

**Table 10:** Summary of statistical uncertainties for events with a ${\mathrm{H}} \rightarrow {\mathrm{b}} {\bar{\mathrm{b}}}$, ${\mathrm{H}} \rightarrow {\mathrm{c}} {\bar{{\mathrm{c}}}}$ or ${\mathrm{H}} \rightarrow {\mathrm{g}} {\mathrm{g}}$ decay, where the Higgs boson is produced by Higgsstrahlung or WW-fusion, at $\sqrt{s} =350\,\text {GeV}$ derived from the template fit as described in the text. All numbers correspond to an integrated luminosity of $500\,\text {fb}^{-1}$

Decay	Statistical uncertainty	
	Higgsstrahlung (%)	${\mathrm{W}} {\mathrm{W}}$-fusion (%)
${\mathrm{H}} \rightarrow {\mathrm{b}} {\bar{\mathrm{b}}}$	0.86	1.9
${\mathrm{H}} \rightarrow {\mathrm{c}} {\bar{{\mathrm{c}}}}$	14	26
${\mathrm{H}} \rightarrow {\mathrm{g}} {\mathrm{g}}$	6.1	10

#### ${\mathrm{H}} \rightarrow \uptau ^{+} \uptau ^{-}$

Because of the neutrino(s) produced in $\uptau$ decays, the signature for ${\mathrm{H}} \rightarrow \uptau ^{+} \uptau ^{-}$ is less distinct than that for other decay modes. The invariant mass of the visible decay products of the $\uptau ^{+} \uptau ^{-}$ system will be less than $m_{{\mathrm{H}}}$, and it is difficult to identify ${\mathrm{H}} \rightarrow \uptau ^{+} \uptau ^{-}$ decays from the ${\mathrm{W}} {\mathrm{W}}$-fusion process or from Higgsstrahlung events where ${\mathrm{Z}} \rightarrow {\nu } \bar{\nu }$. For this reason, the product of $\sigma ({\mathrm{Z}} {\mathrm{H}})\times BR ({\mathrm{H}} \rightarrow \uptau ^{+} \uptau ^{-} )$ is only determined for the case of hadronic ${\mathrm{Z}}$ decays at $\sqrt{s} =350\,\text {GeV}$. In this analysis only hadronic $\uptau$ decays are considered, so the experimental signature is two hadronic jets from ${\mathrm{Z}} \rightarrow \mathrm{q}{\bar{\mathrm{q}}}$ and two isolated low-multiplicity narrow jets from the two tau decays [52]. Candidate $\uptau$ leptons are identified using the TauFinder algorithm [53], which is a seeded-cone based jet-clustering algorithm. The algorithm was optimised to distinguish the tau lepton decay products from hadronic gluon or quark jets. Tau cones are seeded from single tracks ($p_\mathrm {T} >5\,\text {GeV}$). The seeds are used to define narrow cones of 0.05 rad. The cones are required to contain either one or three charged particles (from one- and three-prong tau decays) and further rejection of background from hadronic jets is implemented using cuts on isolation-related variables. Tau cones which contain identified electrons or muons are rejected and only the hadronic one- and three-prong $\uptau$ decays are retained. The $\uptau$ identification efficiency for hadronic tau decays is found to be $73\%$ and the fake rate to mistake a quark for a $\uptau$ is $5\%$. The fake rate is relatively high, but is acceptable as the background from final states with quarks can be suppressed using global event properties.

**Fig. 13:**
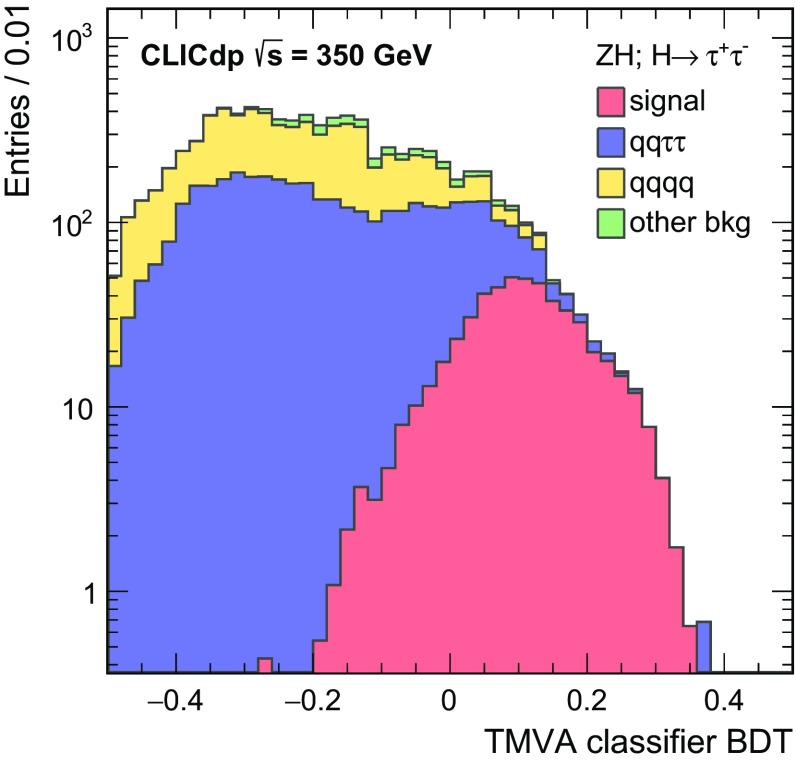
BDT classifier distributions for ${\mathrm{H}} \rightarrow \uptau ^{+} \uptau ^{-}$ events at $\sqrt{s} =350\,\text {GeV}$, showing the signal and main backgrounds as stacked histograms. The distributions are normalised to an integrated luminosity of $500\,\text {fb}^{-1}$

Events with two identified hadronic tau candidates (with opposite net charge) are considered as ${\mathrm{H}} \rightarrow \uptau ^{+} \uptau ^{-}$ decays. Further separation of the signal and background events is achieved using a BDT classifier based on the properties of the tau candidates and global event properties. Seventeen discriminating variables are used as BDT inputs, including the thrust and oblateness of the quark and tau systems, and masses, transverse momenta, and angles in the events. A full list is given in [52]. The resulting BDT distributions for the signal and the backgrounds are shown in Fig. 13. Events passing a cut on the BDT output maximising the significance of the measurement are selected. The cross sections and numbers of selected events for the signal and the dominant background processes are listed in Table 11. The contribution from background processes with photons in the initial state is negligible after the event selection. A template fit to the BDT output distributions leads to:

$$\begin{aligned} \frac{\varDelta [\sigma ({\mathrm{Z}} {\mathrm{H}})\times BR ({\mathrm{H}} \rightarrow \uptau ^{+} \uptau ^{-} )]}{\sigma ({\mathrm{Z}} {\mathrm{H}})\times BR ({\mathrm{H}} \rightarrow \uptau ^{+} \uptau ^{-} )} = 6.2\%. \end{aligned}$$

**Table 11:** Cross sections and numbers of preselected and selected events with BDT > 0.08 (see Fig. 13) for ${\mathrm{e}^{+}}{\mathrm{e}^{-}} \rightarrow {\mathrm{Z}} {\mathrm{H}} ({\mathrm{Z}} \rightarrow \mathrm{q}{\bar{\mathrm{q}}},{\mathrm{H}} \rightarrow \uptau ^{+} \uptau ^{-} )$ signal events and the dominant backgrounds at $\sqrt{s} =350\,\text {GeV}$ assuming an integrated luminosity of $500\,\text {fb}^{-1}$

Process	$\sigma /\text {fb}$	$\varepsilon _\text {presel}$ (%)	$\varepsilon _\text {BDT}$ (%)	$N_\text {BDT}$
${\mathrm{e}^{+}}{\mathrm{e}^{-}} \rightarrow {\mathrm{Z}} {\mathrm{H}};$	5.8	18	59	312
$${\mathrm{Z}} \rightarrow \mathrm{q}{\bar{\mathrm{q}}}, {\mathrm{H}} \rightarrow \uptau ^{+} \uptau ^{-} $$				
${\mathrm{e}^{+}}{\mathrm{e}^{-}} \rightarrow {\mathrm{Z}} {\mathrm{H}};$	4.6	15	2.6	9
$${\mathrm{Z}} \rightarrow \uptau ^{+} \uptau ^{-} , {\mathrm{H}} \rightarrow X$$				
${\mathrm{e}^{+}}{\mathrm{e}^{-}} \rightarrow \mathrm{q} \mathrm{q} \uptau \uptau (\text {non-Higgs})$	70	10	3.3	117
${\mathrm{e}^{+}}{\mathrm{e}^{-}} \rightarrow \mathrm{q} \mathrm{q} \uptau \uptau {\nu } {\nu }$	1.6	9.7	5.1	4
${\mathrm{e}^{+}}{\mathrm{e}^{-}} \rightarrow \mathrm{q} {\bar{\mathrm{q}}} \mathrm{q} {\bar{\mathrm{q}}}$	5850	0.13	0.54	21

**Table 12:** Preselection and selection efficiencies for the ${\mathrm{Z}} {\mathrm{H}}$ signal and most important background processes of the ${\mathrm{H}} \rightarrow {\mathrm{W}} {\mathrm{W}} ^*$ analysis in all three considered Z decay channels. The numbers assume an integrated luminosity of $500\,\text {fb}^{-1}$ at $\sqrt{s} =350\,\text {GeV}$

Process	$\sigma /\text {fb}$	$\varepsilon _\text {presel}$ (%)	$\varepsilon _\text {BDT}$ (%)	$N_\text {BDT}$
${\mathrm{e}^{+}}{\mathrm{e}^{-}} \rightarrow {\mathrm{Z}} {\mathrm{H}};{\mathrm{Z}} \rightarrow {\mathrm{e}^{+}}{\mathrm{e}^{-}};$	0.45	80	53	95
$$\ {\mathrm{H}} \rightarrow {\mathrm{W}} {\mathrm{W}} ^*\rightarrow \mathrm{q} {\bar{\mathrm{q}}} \mathrm{q} {\bar{\mathrm{q}}} $$				
${\mathrm{e}^{+}}{\mathrm{e}^{-}} \rightarrow {\mathrm{Z}} {\mathrm{H}};{\mathrm{Z}} \rightarrow {\mathrm{e}^{+}}{\mathrm{e}^{-}};$	4.1	69	3.4	48
$$\ {\mathrm{H}} \rightarrow other $$				
${\mathrm{e}^{+}}{\mathrm{e}^{-}} \rightarrow \mathrm{q}{\bar{\mathrm{q}}} \mathrm{l} \mathrm{l}$	1700	3.6	0.24	75
${\mathrm{e}^{+}}{\mathrm{e}^{-}} \rightarrow {\mathrm{W}} {\mathrm{W}} {\mathrm{Z}}$	10	3.1	5.9	9
${\mathrm{e}^{+}}{\mathrm{e}^{-}} \rightarrow {\mathrm{Z}} {\mathrm{H}};{\mathrm{Z}} \rightarrow {{{\upmu }}^{+} {{\upmu }}^{-}};$	0.45	87	65	125
$$\ {\mathrm{H}} \rightarrow {\mathrm{W}} {\mathrm{W}} ^*\rightarrow \mathrm{q} {\bar{\mathrm{q}}} \mathrm{q} {\bar{\mathrm{q}}} $$				
${\mathrm{e}^{+}}{\mathrm{e}^{-}} \rightarrow {\mathrm{Z}} {\mathrm{H}};{\mathrm{Z}} \rightarrow {{{\upmu }}^{+} {{\upmu }}^{-}};$	4.1	69	5.2	74
$$\ {\mathrm{H}} \rightarrow other $$				
${\mathrm{e}^{+}}{\mathrm{e}^{-}} \rightarrow \mathrm{q}{\bar{\mathrm{q}}} \mathrm{l} \mathrm{l}$	1700	1.7	0.35	51
${\mathrm{e}^{+}}{\mathrm{e}^{-}} \rightarrow {\mathrm{W}} {\mathrm{W}} {\mathrm{Z}}$	10	2.6	7.1	9
${\mathrm{e}^{+}}{\mathrm{e}^{-}} \rightarrow {\mathrm{Z}} {\mathrm{H}};{\mathrm{Z}} \rightarrow \mathrm{q}{\bar{\mathrm{q}}};$	9.2	71	41	1328
$$\ {\mathrm{H}} \rightarrow {\mathrm{W}} {\mathrm{W}} ^*\rightarrow \mathrm{q} {\bar{\mathrm{q}}} \mathrm{q} {\bar{\mathrm{q}}} $$				
${\mathrm{e}^{+}}{\mathrm{e}^{-}} \rightarrow {\mathrm{Z}} {\mathrm{H}};{\mathrm{Z}} \rightarrow \mathrm{q}{\bar{\mathrm{q}}};$	84	17	10	730
$$\ {\mathrm{H}} \rightarrow other $$				
${\mathrm{e}^{+}}{\mathrm{e}^{-}} \rightarrow \mathrm{q} {\bar{\mathrm{q}}} \mathrm{q} {\bar{\mathrm{q}}}$	5850	18	0.54	2849
${\mathrm{e}^{+}}{\mathrm{e}^{-}} \rightarrow \mathrm{t} {\bar{\mathrm{t}}}$	450	19	2.5	1071
${\mathrm{e}^{+}}{\mathrm{e}^{-}} \rightarrow {\mathrm{W}} {\mathrm{W}} {\mathrm{Z}}$	10	20	18	179

#### ${\mathrm{H}} \rightarrow {\mathrm{W}} {\mathrm{W}} ^*$

In case the Higgs boson decays to a pair of W bosons, only the fully hadronic channel, ${\mathrm{H}} \rightarrow {\mathrm{W}} {\mathrm{W}} ^*\rightarrow \mathrm{q} {\bar{\mathrm{q}}} \mathrm{q} {\bar{\mathrm{q}}}$, allows the reconstruction of the Higgs invariant mass. Two final states in ${\mathrm{e}^{+}}{\mathrm{e}^{-}} \rightarrow {\mathrm{Z}} {\mathrm{H}}$ events have been studied depending on the Z boson decay mode: ${\mathrm{Z}} \rightarrow {\mathrm{l}}^{+} {\mathrm{l}}^{-}$, where $\mathrm{l}$ is an electron or muon, and ${\mathrm{Z}} \rightarrow \mathrm{q}{\bar{\mathrm{q}}}$.

First, isolated electrons and muons from Z decays are identified. Photons in a cone with an opening angle of $3^\circ$ around the lepton candidates are added to their four-momentum as described in Sect. 4.2.

If a leptonic Z candidate is found, four jets are reconstructed from all particles not originating from the Z decay. The jets are paired, with the pair that gives the mass closest to the W boson mass being taken as one W boson candidate, and the other pair taken as the ${\mathrm{W}} ^*$. The events are considered further if the invariant mass of the Z boson candidate is in the range between 70 and $110\,\text {GeV}$ and at least 20 particles are reconstructed.

In events without a leptonic Z candidate, six jets are reconstructed. The jets are grouped into W, Z and Higgs boson candidates by minimising:

$$\begin{aligned} \chi ^2=\frac{(m_{ij}-m_{{\mathrm{W}}})^{2}}{\sigma _{{\mathrm{W}}}^2}+\frac{(m_{kl}-m_{{\mathrm{Z}}})^{2}}{\sigma _{{\mathrm{Z}}}^2}+\frac{(m_{ijmn}-m_{{\mathrm{H}}})^{2}}{\sigma _{{\mathrm{H}}}^2}, \end{aligned}$$

where $m_{ij}$ is the invariant mass of the jet pair used to reconstruct the ${\mathrm{W}}$ candidate, $m_{kl}$ is the invariant mass of the jet pair used to reconstruct the ${\mathrm{Z}}$ candidate, $m_{ijmn}$ is the invariant mass of the four jets used to reconstruct the Higgs candidate and $\sigma _{{\mathrm{W}}, {\mathrm{Z}}, {\mathrm{H}}}$ are the estimated invariant mass resolutions for W, Z and Higgs boson candidates. The preselection cuts for this final state are:

- invariant mass of the Z candidate greater than $40\,\text {GeV}$;
- at least 50 reconstructed particles;
- event thrust of less than 0.95;
- no jet with a b-tag probability of more than 0.95;
- topology of the hadronic system consistent with six jets: $\log _{10}(y_{12})>-2.0$, $\log _{10}(y_{23})>-2.6$, $\log _{10}(y_{34})>-3.0$, $\log _{10}(y_{45})>-3.5$ and $\log _{10}(y_{56})>-4.0$.

**Table 13:** Statistical precisions for the listed processes at $\sqrt{s} =350\,\text {GeV}$ for an integrated luminosity of $500\,\text {fb}^{-1}$

Process	Stat. uncertainty (%)
${\mathrm{e}^{+}}{\mathrm{e}^{-}} \rightarrow {\mathrm{Z}} {\mathrm{H}};{\mathrm{Z}} \rightarrow {\mathrm{e}^{+}}{\mathrm{e}^{-}}; {\mathrm{H}} \rightarrow {\mathrm{W}} {\mathrm{W}} ^{*}\rightarrow \mathrm{q} {\bar{\mathrm{q}}} \mathrm{q} {\bar{\mathrm{q}}}$	16
${\mathrm{e}^{+}}{\mathrm{e}^{-}} \rightarrow {\mathrm{Z}} {\mathrm{H}};{\mathrm{Z}} \rightarrow {{{\upmu }}^{+} {{\upmu }}^{-}}; {\mathrm{H}} \rightarrow {\mathrm{W}} {\mathrm{W}} ^{*}\rightarrow \mathrm{q} {\bar{\mathrm{q}}} \mathrm{q} {\bar{\mathrm{q}}}$	13
${\mathrm{e}^{+}}{\mathrm{e}^{-}} \rightarrow {\mathrm{Z}} {\mathrm{H}};{\mathrm{Z}} \rightarrow \mathrm{q}{\bar{\mathrm{q}}}; {\mathrm{H}} \rightarrow {\mathrm{W}} {\mathrm{W}} ^{*}\rightarrow \mathrm{q} {\bar{\mathrm{q}}} \mathrm{q} {\bar{\mathrm{q}}}$	5.9

For both final states, BDT classifiers are used to suppress the backgrounds further. The event selection for the signal processes and the most relevant background samples is summarised in Table 12. The expected precisions for the measurement of the investigated processes are summarised in Table 13. The best precision is achieved using the ${\mathrm{Z}} \rightarrow \mathrm{q}{\bar{\mathrm{q}}}$ decay due to its large branching ratio compared to leptonic decays. The selection of ${\mathrm{Z}} \rightarrow {\mathrm{e}^{+}}{\mathrm{e}^{-}}$ events is more difficult compared to ${\mathrm{Z}} \rightarrow {{{\upmu }}^{+} {{\upmu }}^{-}}$ events because the ${\mathrm{e}^{+}}{\mathrm{e}^{-}} \rightarrow \mathrm{q}{\bar{\mathrm{q}}} \mathrm{l} \mathrm{l}$ background sample contains more events with electron pairs than events with muon pairs. Hence the precision achieved using ${\mathrm{Z}} \rightarrow {{{\upmu }}^{+} {{\upmu }}^{-}}$ decays is somewhat better compared to that obtained using ${\mathrm{Z}} \rightarrow {\mathrm{e}^{+}}{\mathrm{e}^{-}}$ decays. The combined precision for an integrated luminosity of 500 $\text {fb}^{-1}$ is:

$$\begin{aligned} \frac{\varDelta [\sigma ({\mathrm{Z}} {\mathrm{H}})\times BR ({\mathrm{H}} \rightarrow {\mathrm{W}} {\mathrm{W}} ^*)]}{\sigma ({\mathrm{Z}} {\mathrm{H}})\times BR ({\mathrm{H}} \rightarrow {\mathrm{W}} {\mathrm{W}} ^*)} = 5.1\% , \end{aligned}$$

which is dominated by the final state with hadronic Z boson decays.

**Table 14:** Preselection and selection efficiencies for the signal and most important background processes in the ${\mathrm{H}} \rightarrow {\mathrm{b}} {\bar{\mathrm{b}}}$, ${\mathrm{H}} \rightarrow {\mathrm{c}} {\bar{{\mathrm{c}}}}$ and ${\mathrm{H}} \rightarrow {\mathrm{g}} {\mathrm{g}}$ analysis. The numbers of events correspond to 1.5 $\text {ab}^{-1}$ at $\sqrt{s} =1.4\,\text {TeV}$

Process	$\sigma /\text {fb}$	$\varepsilon _\text {presel}$ (%)	$\varepsilon _\text {BDT}$ (%)	$N_\text {BDT}$
${\mathrm{e}^{+}}{\mathrm{e}^{-}} \rightarrow {\mathrm{H}} {{\nu }}_{\!\mathrm{e}} {\bar{{\nu }}}_{\!\mathrm{e}} ; {\mathrm{H}} \rightarrow {\mathrm{b}} {\bar{\mathrm{b}}}$	137	85	38	65,400
${\mathrm{e}^{+}}{\mathrm{e}^{-}} \rightarrow {\mathrm{H}} {{\nu }}_{\!\mathrm{e}} {\bar{{\nu }}}_{\!\mathrm{e}} ; {\mathrm{H}} \rightarrow {\mathrm{c}} {\bar{{\mathrm{c}}}}$	6.9	87	42	3790
${\mathrm{e}^{+}}{\mathrm{e}^{-}} \rightarrow {\mathrm{H}} {{\nu }}_{\!\mathrm{e}} {\bar{{\nu }}}_{\!\mathrm{e}} ; {\mathrm{H}} \rightarrow {\mathrm{g}} {\mathrm{g}}$	20.7	82	40	10,100
${\mathrm{e}^{+}}{\mathrm{e}^{-}} \rightarrow \mathrm{q} {\bar{\mathrm{q}}} {\nu } \bar{\nu }$	788	76	2.1	18,500
${\mathrm{e}^{+}}{\mathrm{e}^{-}} \rightarrow \mathrm{q} {\bar{\mathrm{q}}} \mathrm{l} {\nu }$	4310	40	0.91	23,600
$\mathrm{e} ^{\pm } {\upgamma } \rightarrow \mathrm{q} {\bar{\mathrm{q}}} \mathrm{e}$	16,600	14	0.54	18,500
$\mathrm{e} ^{\pm } {\upgamma } \rightarrow \mathrm{q} {\bar{\mathrm{q}}} {\nu }$	29,300	60	0.64	170,000
${\upgamma } {\upgamma } \rightarrow \mathrm{q} {\bar{\mathrm{q}}}$	76,600	4.2	0.47	22,200

**Table 15:** Preselection and selection efficiencies for the signal and most important background processes in the ${\mathrm{H}} \rightarrow {\mathrm{b}} {\bar{\mathrm{b}}}$, ${\mathrm{H}} \rightarrow {\mathrm{c}} {\bar{{\mathrm{c}}}}$ and ${\mathrm{H}} \rightarrow {\mathrm{g}} {\mathrm{g}}$ analysis. The numbers of events correspond to 2 $\text {ab}^{-1}$ at $\sqrt{s} =3\,\text {TeV}$

Process	$\sigma /\text {fb}$	$\varepsilon _\text {presel}$ (%)	$\varepsilon _\text {BDT}$ (%)	$N_\text {BDT}$
${\mathrm{e}^{+}}{\mathrm{e}^{-}} \rightarrow {\mathrm{H}} {{\nu }}_{\!\mathrm{e}} {\bar{{\nu }}}_{\!\mathrm{e}} ; {\mathrm{H}} \rightarrow {\mathrm{b}} {\bar{\mathrm{b}}}$	233	74	35	120,000
${\mathrm{e}^{+}}{\mathrm{e}^{-}} \rightarrow {\mathrm{H}} {{\nu }}_{\!\mathrm{e}} {\bar{{\nu }}}_{\!\mathrm{e}} ; {\mathrm{H}} \rightarrow {\mathrm{c}} {\bar{{\mathrm{c}}}}$	11.7	75	36	6380
${\mathrm{e}^{+}}{\mathrm{e}^{-}} \rightarrow {\mathrm{H}} {{\nu }}_{\!\mathrm{e}} {\bar{{\nu }}}_{\!\mathrm{e}} ; {\mathrm{H}} \rightarrow {\mathrm{g}} {\mathrm{g}}$	35.2	69	35	16,800
${\mathrm{e}^{+}}{\mathrm{e}^{-}} \rightarrow \mathrm{q} {\bar{\mathrm{q}}} {\nu } \bar{\nu }$	1300	67	2.7	47,400
${\mathrm{e}^{+}}{\mathrm{e}^{-}} \rightarrow \mathrm{q} {\bar{\mathrm{q}}} \mathrm{e} {\nu }$	5260	45	1.1	52,200
$\mathrm{e} ^{\pm } {\upgamma } \rightarrow \mathrm{q} {\bar{\mathrm{q}}} \mathrm{e}$	20,500	13	2.3	118,000
$\mathrm{e} ^{\pm } {\upgamma } \rightarrow \mathrm{q} {\bar{\mathrm{q}}} {\nu }$	46,400	46	0.92	394,000
${\upgamma } {\upgamma } \rightarrow \mathrm{q} {\bar{\mathrm{q}}}$	92,200	7.0	1.6	207,000

## WW-fusion at $\sqrt{s} >1\,\text {TeV}$

This section presents measurements of Higgs decays from the ${\mathrm{W}} {\mathrm{W}}$-fusion process at CLIC with centre-of-mass energies of 1.4 $\text {TeV}$ and 3 $\text {TeV}$. The Higgs self-coupling measurement, which is also accessed in ${\mathrm{W}} {\mathrm{W}}$-fusion production, is discussed in Sect. 9. The cross section of the Higgs production via the vector boson fusion process ${\mathrm{e}^{+}}{\mathrm{e}^{-}} \rightarrow {\mathrm{H}} {{\nu }}_{\!\mathrm{e}} {\bar{{\nu }}}_{\!\mathrm{e}}$ scales with $\log (s)$ and becomes the dominating Higgs production process in ${\mathrm{e}^{+}}{\mathrm{e}^{-}}$ collisions with $\sqrt{s} >500\,\text {GeV}$. The respective cross sections for ${\mathrm{e}^{+}}{\mathrm{e}^{-}} \rightarrow {\mathrm{H}} {{\nu }}_{\!\mathrm{e}} {\bar{{\nu }}}_{\!\mathrm{e}}$ at $\sqrt{s} = 1.4\,\text {TeV}$ and 3 $\text {TeV}$ are approximately 244 $\text {fb}$ and 415 $\text {fb}$, respectively, including the effects of the CLIC beamstrahlung spectrum and ISR. The relatively large cross sections at the higher energies allow the Higgs decay modes to be probed with high statistical precision and provide access to rarer Higgs decays, such as ${\mathrm{H}} \rightarrow {{{\upmu }}^{+} {{\upmu }}^{-}}$.

Since ${\mathrm{W}} {\mathrm{W}}$-fusion ${\mathrm{e}^{+}}{\mathrm{e}^{-}} \rightarrow {\mathrm{H}} {{\nu }}_{\!\mathrm{e}} {\bar{{\nu }}}_{\!\mathrm{e}}$ proceeds through the *t*-channel, the Higgs boson is typically boosted along the beam direction and the presence of neutrinos in the final state can result in significant missing $p_\mathrm {T}$. Because of the missing transverse and longitudinal momentum, the experimental signatures for ${\mathrm{H}} {{\nu }}_{\!\mathrm{e}} {\bar{{\nu }}}_{\!\mathrm{e}}$ production are relatively well separated from most SM backgrounds. At $\sqrt{s} = 350\,$GeV, the main SM background processes are two- and four-fermion production, ${\mathrm{e}^{+}}{\mathrm{e}^{-}} \rightarrow 2f$ and ${\mathrm{e}^{+}}{\mathrm{e}^{-}} \rightarrow 4f$. At higher energies, backgrounds from ${\upgamma } {\upgamma }$ and ${\upgamma } \mathrm{e} ^{\pm }$ hard interactions become increasingly relevant for measurements of Higgs boson production in ${\mathrm{W}} {\mathrm{W}}$-fusion. Additionally, pile-up of relatively soft ${{\upgamma } {\upgamma } \rightarrow \text {hadrons}}$ events with the primary interaction occurs. However, this background of relatively low-$p_\mathrm {T}$ particles is largely mitigated through the timing cuts and jet finding strategy outlined in Sect. 4.

### ${\mathrm{H}} \rightarrow {\mathrm{b}} {\bar{\mathrm{b}}} , {\mathrm{c}} {\bar{{\mathrm{c}}}} , {\mathrm{g}} {\mathrm{g}}$

The physics potential for the measurement of hadronic Higgs decays at the centre-of-mass energies of 1.4 and 3 $\text {TeV}$ was studied using the CLIC_SiD detector model. The signatures for ${\mathrm{H}} \rightarrow {\mathrm{b}} {\bar{\mathrm{b}}}$, ${\mathrm{H}} \rightarrow {\mathrm{c}} {\bar{{\mathrm{c}}}}$ and ${\mathrm{H}} \rightarrow {\mathrm{g}} {\mathrm{g}}$ decays in ${\mathrm{e}^{+}}{\mathrm{e}^{-}} \rightarrow {\mathrm{H}} {{\nu }}_{\!\mathrm{e}} {\bar{{\nu }}}_{\!\mathrm{e}}$ events are two jets and missing energy. Flavour tagging information from LcfiPlus is used to separate the investigated Higgs boson decay modes in the selected event sample. The invariant mass of the reconstructed di-jet system provides rejection against background processes, e.g. hadronic ${\mathrm{Z}}$ boson decays.

At both centre-of-mass energies, an invariant mass of the di-jet system in the range from 60 to $160\,\text {GeV}$ and a distance between both jets in the $\eta -\phi$ plane of less than 4 are required. The energy sum of the two jets must exceed 75 $\text {GeV}$ and a missing momentum of at least 20 $\text {GeV}$ is required. The efficiencies of these preselection cuts on the signal and dominant background samples are listed in Tables 14 and 15 for the centre-of-mass energies of 1.4 and 3 $\text {TeV}$, respectively.

The backgrounds are suppressed further using a single BDT at each energy. The samples of signal events used to train these classifiers consist of equal amounts of ${\mathrm{H}} \rightarrow {\mathrm{b}} {\bar{\mathrm{b}}}$, ${\mathrm{H}} \rightarrow {\mathrm{c}} {\bar{{\mathrm{c}}}}$, and ${\mathrm{H}} \rightarrow {\mathrm{g}} {\mathrm{g}}$ events, while the different processes in the background sample were normalised according to their respective cross sections. No flavour tagging information is used in the event selection. This leads to classifiers with similar selection efficiencies for events with the different signal Higgs decays.

The fractions of signal events with ${\mathrm{H}} \rightarrow {\mathrm{b}} {\bar{\mathrm{b}}}$, ${\mathrm{H}} \rightarrow {\mathrm{c}} {\bar{{\mathrm{c}}}}$ and ${\mathrm{H}} \rightarrow {\mathrm{g}} {\mathrm{g}}$ decays in the selected event samples are extracted from the two-dimensional distributions of the ${\mathrm{b}} {\bar{\mathrm{b}}}$ versus ${\mathrm{c}} {\bar{{\mathrm{c}}}}$ likelihood variables for the two reconstructed jets as defined in Sect. 5.2. The normalisations of the backgrounds from other Higgs decays and non-Higgs events are fixed and expected to be provided by other measurements. The results of these fits are shown in Tables 16 and 17 at 1.4 and 3 $\text {TeV}$, respectively.

The expected precisions obtained at 1.4 and 3 $\text {TeV}$ are similar although the number of signal events is about twice as large at 3 $\text {TeV}$ compared to 1.4 $\text {TeV}$. The main reasons for this are that the jet reconstruction and flavour tagging are more challenging at 3 $\text {TeV}$, since the jets from the Higgs decay tend more towards the beam axis, and the impact of the beam-induced backgrounds is larger compared to 1.4 $\text {TeV}$. In addition, the cross sections for the most important background processes rise with $\sqrt{s}$ (see Tables 14 and 15).

**Table 16:** Statistical precisions for the listed processes from the fit described in the text at $\sqrt{s} =1.4\,\text {TeV}$ for an integrated luminosity of 1.5 $\text {ab}^{-1}$

Process	Statistical uncertainty (%)
${\mathrm{e}^{+}}{\mathrm{e}^{-}} \rightarrow {\mathrm{H}} {{\nu }}_{\!\mathrm{e}} {\bar{{\nu }}}_{\!\mathrm{e}} ; {\mathrm{H}} \rightarrow {\mathrm{b}} {\bar{\mathrm{b}}}$	0.4
${\mathrm{e}^{+}}{\mathrm{e}^{-}} \rightarrow {\mathrm{H}} {{\nu }}_{\!\mathrm{e}} {\bar{{\nu }}}_{\!\mathrm{e}} ; {\mathrm{H}} \rightarrow {\mathrm{c}} {\bar{{\mathrm{c}}}}$	6.1
${\mathrm{e}^{+}}{\mathrm{e}^{-}} \rightarrow {\mathrm{H}} {{\nu }}_{\!\mathrm{e}} {\bar{{\nu }}}_{\!\mathrm{e}} ; {\mathrm{H}} \rightarrow {\mathrm{g}} {\mathrm{g}}$	5.0

**Table 17:** Statistical precisions for the listed processes from the fit described in the text at $\sqrt{s} =3\,\text {TeV}$ for an integrated luminosity of 2 $\text {ab}^{-1}$

Process	Statistical uncertainty (%)
${\mathrm{e}^{+}}{\mathrm{e}^{-}} \rightarrow {\mathrm{H}} {{\nu }}_{\!\mathrm{e}} {\bar{{\nu }}}_{\!\mathrm{e}} ; {\mathrm{H}} \rightarrow {\mathrm{b}} {\bar{\mathrm{b}}}$	0.3
${\mathrm{e}^{+}}{\mathrm{e}^{-}} \rightarrow {\mathrm{H}} {{\nu }}_{\!\mathrm{e}} {\bar{{\nu }}}_{\!\mathrm{e}} ; {\mathrm{H}} \rightarrow {\mathrm{c}} {\bar{{\mathrm{c}}}}$	6.9
${\mathrm{e}^{+}}{\mathrm{e}^{-}} \rightarrow {\mathrm{H}} {{\nu }}_{\!\mathrm{e}} {\bar{{\nu }}}_{\!\mathrm{e}} ; {\mathrm{H}} \rightarrow {\mathrm{g}} {\mathrm{g}}$	4.3

### ${\mathrm{H}} \rightarrow \uptau ^{+} \uptau ^{-}$

The sensitivity for the measurement of $\sigma ({\mathrm{e}^{+}}{\mathrm{e}^{-}} \rightarrow {\mathrm{H}} {{\nu }}_{\!\mathrm{e}} {\bar{{\nu }}}_{\!\mathrm{e}} )\times BR ({\mathrm{H}} \rightarrow \uptau ^{+} \uptau ^{-} )$ at CLIC has been studied using the CLIC_ILD detector model at centre-of-mass energies of 1.4 and 3 $\text {TeV}$  [54]. For a SM Higgs with a mass of 126 $\text {GeV}$, $BR ({\mathrm{H}} \rightarrow \uptau ^{+} \uptau ^{-} )=6.2\%$, resulting in an effective signal cross section of 15.0 $\text {fb}$ at $\sqrt{s} = 1.4\,\text {TeV}$ and 25.5 $\text {fb}$ at $\sqrt{s} = 3\,\text {TeV}$.

**Fig. 14:**
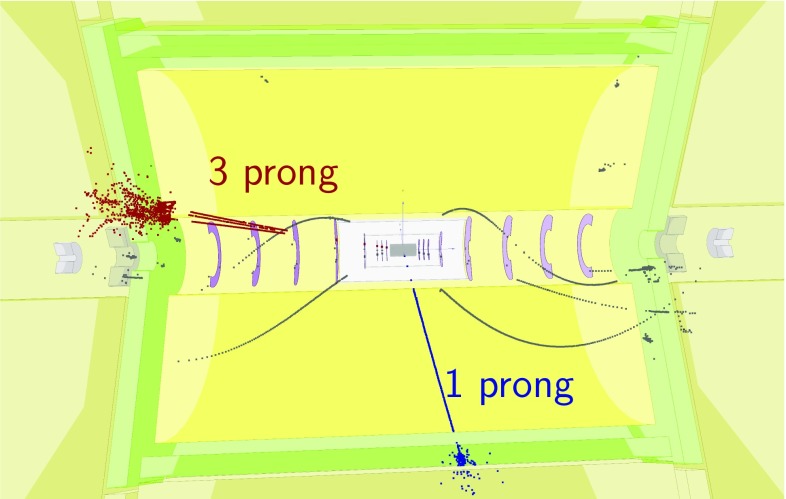
Event display of a ${\mathrm{H}} \rightarrow \uptau ^{+} \uptau ^{-}$ event at $\sqrt{s} =1.4\,\text {TeV}$ in the CLIC_ILD detector. A 1-prong tau decay is visible in the central part of the detector (*blue*). The other tau lepton decays to three charged particles and is reconstructed in the forward direction (*red*). A few soft particles from beam-induced backgrounds are also visible (*grey*)

The experimental signature is two relatively high-momentum narrow jets from the two tau decays and significant missing transverse and longitudinal momenta. A typical event display is shown in Fig. 14. The analysis is restricted to hadronic $\uptau$ decays, which are identified using the TauFinder algorithm, as described in Sect. 5.2.2. The TauFinder algorithm parameters were tuned using the ${\mathrm{H}} \rightarrow \uptau ^{+} \uptau ^{-}$ signal events and ${\mathrm{e}^{+}}{\mathrm{e}^{-}} \rightarrow \mathrm{q}{\bar{\mathrm{q}}} \nu \bar{\nu }$ background events. The working point has a $\uptau$ selection efficiency of 70% (60%) with a quark jet fake rate of 7% (9%) at $\sqrt{s} = 1.4\,\text {TeV}$ ($\sqrt{s} = 3\,\text {TeV}$). All relevant SM backgrounds are taken into account, including ${\upgamma } {\upgamma }$ and ${\upgamma } \mathrm{e} ^{\pm }$ collisions. The most significant backgrounds are ${\mathrm{e}^{+}}{\mathrm{e}^{-}} \rightarrow \uptau ^{+} \uptau ^{-} \nu \bar{\nu }$, $\mathrm{e} ^{\pm } {\upgamma } \rightarrow \uptau ^{+} \uptau ^{-} \mathrm{e} ^{\pm }$ and ${\upgamma } {\upgamma } \rightarrow \uptau ^{+} \uptau ^{-} \nu \bar{\nu }$. The latter two processes become increasingly important at higher $\sqrt{s}$, due to the increasing number of beamstrahlung photons. Backgrounds from Higgs decays other than ${\mathrm{H}} \rightarrow \uptau ^{+} \uptau ^{-}$ are expected to be negligible [55].

The event preselection requires two identified $\uptau$ leptons, both of which must be within the polar angle range $15^\circ< \theta (\uptau ) < 165^\circ$ and have $p_\mathrm {T} (\uptau ) > 25\,\text {GeV}$. To reject back-to-back or nearby tau leptons, the angle between the two tau candidates must satisfy $29^\circ< \varDelta \theta (\uptau \uptau ) < 177^\circ$. The visible invariant mass $m(\uptau \uptau )$ and the visible transverse mass $m_\text {T}(\uptau \uptau )$ of the two tau candidates must satisfy $45\,\text {GeV}< m(\uptau \uptau ) < 130\,\text {GeV}$ and $m_\text {T}(\uptau \uptau ) < 20\,\text {GeV}$. Finally the event thrust must be less than 0.99.

**Table 18:** Preselection and selection efficiencies for the signal and most important background processes in the ${\mathrm{H}} \rightarrow \uptau ^{+} \uptau ^{-}$ analysis. The numbers of events correspond to 1.5 $\text {ab}^{-1}$ at $\sqrt{s} =1.4\,\text {TeV}$. The cross sections for the backgrounds include cuts on the kinematic properties of the tau lepton pair applied at generator level. The preselection efficiencies include the reconstruction of two hadronic tau lepton decays per event

Process	$\sigma /\text {fb}$	$\varepsilon _\text {presel}$ (%)	$\varepsilon _\text {BDT}$ (%)	$N_\text {BDT}$
${\mathrm{e}^{+}}{\mathrm{e}^{-}} \rightarrow {\mathrm{H}} {{\nu }}_{\!\mathrm{e}} {\bar{{\nu }}}_{\!\mathrm{e}} ; {\mathrm{H}} \rightarrow \uptau ^{+} \uptau ^{-}$	15.0	9.3	39	814
${\mathrm{e}^{+}}{\mathrm{e}^{-}} \rightarrow \uptau ^{+} \uptau ^{-} \nu \bar{\nu }$	38.5	5.0	18	528
$\mathrm{e} ^{\pm } {\upgamma } \rightarrow \uptau ^{+} \uptau ^{-} \mathrm{e} ^{\pm }$	2140	1.9	0.075	45
${\upgamma } {\upgamma } \rightarrow \uptau ^{+} \uptau ^{-} (\nu \bar{\nu }~ or ~{\mathrm{l}}^{-} {\mathrm{l}}^{+})$	86.7	2.7	2.3	79

**Table 19:** Preselection and selection efficiencies for the signal and most important background processes in the ${\mathrm{H}} \rightarrow \uptau ^{+} \uptau ^{-}$ analysis. The numbers of events correspond to 2 $\text {ab}^{-1}$ at $\sqrt{s} =3\,\text {TeV}$. The cross sections for the backgrounds include cuts on the kinematic properties of the tau lepton pair applied at generator level. The preselection efficiencies include the reconstruction of two hadronic tau lepton decays per event

Process	$\sigma /\text {fb}$	$\varepsilon _\text {presel}$ (%)	$\varepsilon _\text {BDT}$ (%)	$N_\text {BDT}$
${\mathrm{e}^{+}}{\mathrm{e}^{-}} \rightarrow {\mathrm{H}} {{\nu }}_{\!\mathrm{e}} {\bar{{\nu }}}_{\!\mathrm{e}} ; {\mathrm{H}} \rightarrow \uptau ^{+} \uptau ^{-}$	25.5	6.7	23	787
${\mathrm{e}^{+}}{\mathrm{e}^{-}} \rightarrow \uptau ^{+} \uptau ^{-} \nu \bar{\nu }$	39.2	5.7	11	498
$\mathrm{e} ^{\pm } {\upgamma } \rightarrow \uptau ^{+} \uptau ^{-} \mathrm{e} ^{\pm }$	2393	2.0	0.26	246
${\upgamma } {\upgamma } \rightarrow \uptau ^{+} \uptau ^{-} (\nu \bar{\nu }~ or ~{\mathrm{l}}^{-} {\mathrm{l}}^{+})$	158	2.0	0.14	9

Events passing the preselection are classified as either signal or SM background using a BDT classifier. The kinematic variables used in the classifier are $m(\uptau \uptau )$, $m_\text {T}(\uptau \uptau )$, event shape variables (such as thrust and oblateness), the missing $p_\mathrm {T}$, the polar angle of the missing momentum vector $|\cos {\varvec{\theta }}_{\text {miss}}|$ and the total reconstructed energy excluding the Higgs candidate. The event selection for the signal and the most relevant background processes is summarised in Table 18 for $\sqrt{s} = 1.4\,\text {TeV}$ and in Table 19 for $\sqrt{s} = 3\,\text {TeV}$. Rather than applying a simple cut, the full BDT shape information is used in a template fit. The resulting statistical uncertainties for $1.5\,\text {ab}^{-1}$ at $\sqrt{s} =1.4\,\text {TeV}$ and $2.0\,\text {ab}^{-1}$ at $\sqrt{s} =3\,\text {TeV}$ are:

$$\begin{aligned} \frac{\varDelta [\sigma ({\mathrm{H}} {{\nu }}_{\!\mathrm{e}} {\bar{{\nu }}}_{\!\mathrm{e}} )\times BR ({\mathrm{H}} \rightarrow \uptau ^{+} \uptau ^{-} )]}{\sigma ({\mathrm{H}} {{\nu }}_{\!\mathrm{e}} {\bar{{\nu }}}_{\!\mathrm{e}} )\times BR ({\mathrm{H}} \rightarrow \uptau ^{+} \uptau ^{-} )}&= 4.2\% \ \text {at} \ 1.4\,\text {TeV}, \\ \frac{\varDelta [\sigma ({\mathrm{H}} {{\nu }}_{\!\mathrm{e}} {\bar{{\nu }}}_{\!\mathrm{e}} )\times BR ({\mathrm{H}} \rightarrow \uptau ^{+} \uptau ^{-} )]}{\sigma ({\mathrm{H}} {{\nu }}_{\!\mathrm{e}} {\bar{{\nu }}}_{\!\mathrm{e}} )\times BR ({\mathrm{H}} \rightarrow \uptau ^{+} \uptau ^{-} )}&= 4.4\% \ \text {at} \ 3\,\text {TeV}. \end{aligned}$$

**Table 20:** Summary of the ${\mathrm{H}} \rightarrow {\mathrm{W}} {\mathrm{W}} ^*\rightarrow \mathrm{q} {\bar{\mathrm{q}}} \mathrm{q} {\bar{\mathrm{q}}}$ event selection at $\sqrt{s} =1.4\,\text {TeV}$, giving the raw cross sections, preselection efficiency, selection efficiency for a likelihood cut of $\mathcal{{L}}>0.35$, and the expected numbers of events passing the event selection for an integrated luminosity of $1.5\,\text {ab}^{-1}$

Process	$\sigma /\text {fb}$	$\varepsilon _\text {presel}$ (%)	$\varepsilon _{\mathcal{{L}}>0.35}$ (%)	$N_{\mathcal{{L}}>0.35}$
All ${\mathrm{H}} {{\nu }}_{\!\mathrm{e}} {\bar{{\nu }}}_{\!\mathrm{e}}$	244	14.6	21	11,101
${\mathrm{H}} \rightarrow {\mathrm{W}} {\mathrm{W}} ^*\rightarrow \mathrm{q} {\bar{\mathrm{q}}} \mathrm{q} {\bar{\mathrm{q}}}$		32	56	7518
${\mathrm{H}} \rightarrow {\mathrm{W}} {\mathrm{W}} ^*\rightarrow \mathrm{q} {\bar{\mathrm{q}}} \mathrm{l} {\nu }$		4.4	14	253
${\mathrm{H}} \rightarrow {\mathrm{b}} {\bar{\mathrm{b}}}$		1.9	21	774
${\mathrm{H}} \rightarrow {\mathrm{c}} {\bar{{\mathrm{c}}}}$		8.1	26	209
${\mathrm{H}} \rightarrow {\mathrm{g}} {\mathrm{g}}$		19	37	1736
${\mathrm{H}} \rightarrow {\mathrm{Z}} {\mathrm{Z}} ^*$		12	42	556
${\mathrm{H}} \rightarrow \text {other}$		0.7	29	55
${\mathrm{e}^{+}}{\mathrm{e}^{-}} \rightarrow \mathrm{q} {\bar{\mathrm{q}}} {\nu } \bar{\nu }$	788	4.6	4.1	2225
${\mathrm{e}^{+}}{\mathrm{e}^{-}} \rightarrow \mathrm{q} {\bar{\mathrm{q}}} \mathrm{q} {\bar{\mathrm{q}}} \mathrm{l} {\nu }$	115	0.1	25	43
${\mathrm{e}^{+}}{\mathrm{e}^{-}} \rightarrow \mathrm{q} {\bar{\mathrm{q}}} \mathrm{q} {\bar{\mathrm{q}}} {\nu } \bar{\nu }$	24.7	0.8	44	130
${\upgamma } \mathrm{e} ^{\pm } \rightarrow \mathrm{q} {\bar{\mathrm{q}}} \mathrm{q} {\bar{\mathrm{q}}} {\nu }$	254	1.8	20	1389

Similar to the observations described in Sect. 6.1, the expected precisions at 1.4 $\text {TeV}$ and 3 $\text {TeV}$ are similar. The identification of tau leptons is more challenging at 3 TeV where the impact of the beam-induced backgrounds is larger and the tau leptons from Higgs decays in signal events tend more towards the beam axis.

### ${\mathrm{H}} \rightarrow {\mathrm{W}} {\mathrm{W}} ^*$

The signature for ${\mathrm{H}} \rightarrow {\mathrm{W}} {\mathrm{W}} ^*$ decays in ${\mathrm{e}^{+}}{\mathrm{e}^{-}} \rightarrow {\mathrm{H}} {{\nu }}_{\!\mathrm{e}} {\bar{{\nu }}}_{\!\mathrm{e}}$ depends on the ${\mathrm{W}} {\mathrm{W}} ^*$ decay modes. As $m_{{\mathrm{H}}} < 2m_{{\mathrm{W}}}$, at least one of the ${\mathrm{W}}$-bosons is off mass-shell. Studies for two different final states are described in the following. The presence of a charged lepton in the ${\mathrm{W}} {\mathrm{W}} ^*\rightarrow \mathrm{q} {\bar{\mathrm{q}}} \mathrm{l} {\nu }$ final state suppresses backgrounds from other Higgs decays. However, the invariant mass of the Higgs boson in ${\mathrm{H}} \rightarrow {\mathrm{W}} {\mathrm{W}} ^*$ decays can be reconstructed for fully-hadronic decays alone, ${\mathrm{W}} {\mathrm{W}} ^*\rightarrow \mathrm{q} {\bar{\mathrm{q}}} \mathrm{q} {\bar{\mathrm{q}}}$.

#### ${\mathrm{W}} {\mathrm{W}} ^*\rightarrow \mathrm{q} {\bar{\mathrm{q}}} \mathrm{q} {\bar{\mathrm{q}}}$

The experimental signature for ${\mathrm{H}} {{\nu }}_{\!\mathrm{e}} {\bar{{\nu }}}_{\!\mathrm{e}}$ production with ${\mathrm{H}} \rightarrow {\mathrm{W}} {\mathrm{W}} ^*\rightarrow \mathrm{q} {\bar{\mathrm{q}}} \mathrm{q} {\bar{\mathrm{q}}}$ is a four-jet final state with missing $p_\mathrm {T}$ and a total invariant mass consistent with the Higgs mass, where one pair of jets has a mass consistent with $m_{{\mathrm{W}}}$.

The ${\mathrm{H}} \rightarrow {\mathrm{W}} {\mathrm{W}} ^*$ event selection has been studied at $\sqrt{s} =1.4\,\text {TeV}$ using the CLIC_ILD detector model. It proceeds in two separate stages: a set of preselection cuts designed to reduce the backgrounds from large cross section processes such as ${\mathrm{e}^{+}}{\mathrm{e}^{-}} \rightarrow \mathrm{q} {\bar{\mathrm{q}}}$ and ${\mathrm{e}^{+}}{\mathrm{e}^{-}} \rightarrow \mathrm{q} {\bar{\mathrm{q}}} \mathrm{q} {\bar{\mathrm{q}}}$; followed by a likelihood-based multivariate event selection. The preselection variables are formed by forcing each event into four jets using the Durham jet finder. Of the three possible jet associations with candidate ${\mathrm{W}}$ bosons, (12)(34), (13)(24) or (14)(23), the one giving a di-jet invariant mass closest to $m_{{\mathrm{W}}}$ is selected. The preselection requires that there is no high-energy electron or muon with $E_{\ell }>30\,\text {GeV}$. Further preselection cuts are made on the properties of the jets, the invariant masses of the off-shell and on-shell ${\mathrm{W}}$ boson candidates, the Higgs boson candidate, the total visible energy and the missing transverse momentum. In addition, in order to reject ${\mathrm{H}} \rightarrow {\mathrm{b}} {\bar{\mathrm{b}}}$ decays, the event is forced into a two-jet topology and flavour tagging is applied to the two jets. Events where at least one jet has a ${\mathrm{b}}$-tag probability above 0.95 are rejected as part of the preselection. The cross sections and preselection efficiencies for the signal and main background processes are listed in Table 20. After the preselection, the main backgrounds are ${\mathrm{e}^{+}}{\mathrm{e}^{-}} \rightarrow \mathrm{q} {\bar{\mathrm{q}}} {\nu } \bar{\nu }$, ${\upgamma } \mathrm{e} ^{\pm } \rightarrow \mathrm{q} {\bar{\mathrm{q}}} \mathrm{q} {\bar{\mathrm{q}}} {\nu }$ and other Higgs decay modes, predominantly ${\mathrm{H}} \rightarrow {\mathrm{b}} {\bar{\mathrm{b}}}$ and ${\mathrm{H}} \rightarrow {\mathrm{g}} {\mathrm{g}}$, where QCD radiation in the parton shower can lead to a four-jet topology.

**Fig. 15:**
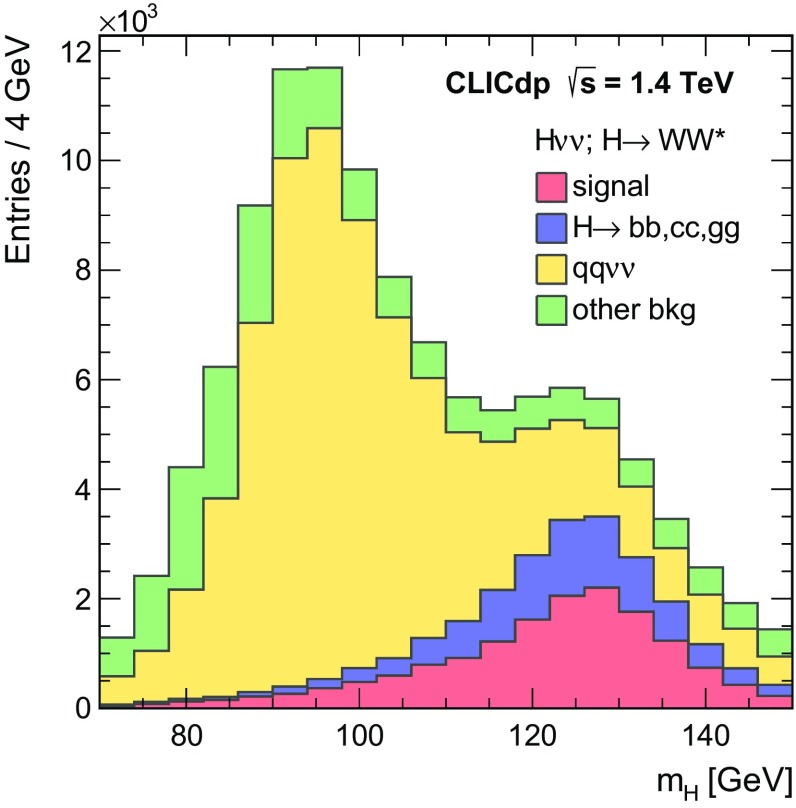
Reconstructed Higgs invariant mass distributions for preselected ${\mathrm{H}} \rightarrow {\mathrm{W}} {\mathrm{W}} ^*\rightarrow \mathrm{q} {\bar{\mathrm{q}}} \mathrm{q} {\bar{\mathrm{q}}}$ events at $\sqrt{s} = 1.4\,\text {TeV}$, showing the signal and main backgrounds as stacked histograms. The distributions are normalised to an integrated luminosity of $1.5\,\text {ab}^{-1}$

A relative likelihood selection is used to classify all events passing the preselection cuts. Five event categories including the signal are considered. The relative likelihood of an event being signal is estimated as:

$$\begin{aligned} \mathcal{{L}} = \frac{L({\mathrm{H}} \rightarrow {\mathrm{W}} {\mathrm{W}} ^*\rightarrow \mathrm{q} {\bar{\mathrm{q}}} \mathrm{q} {\bar{\mathrm{q}}})}{L({\mathrm{H}} \rightarrow {\mathrm{W}} {\mathrm{W}} ^*\rightarrow \mathrm{q} {\bar{\mathrm{q}}} \mathrm{q} {\bar{\mathrm{q}}})+ L_1 + L_2 + L_3 + L_4}, \end{aligned}$$

where $L_i$ represents the likelihood for four background categories: ${\mathrm{H}} \rightarrow {\mathrm{b}} {\bar{\mathrm{b}}}$, ${\mathrm{H}} \rightarrow {\mathrm{g}} {\mathrm{g}}$, ${\mathrm{e}^{+}}{\mathrm{e}^{-}} \rightarrow \mathrm{q} {\bar{\mathrm{q}}} {\nu } \bar{\nu }$ and ${\upgamma } \mathrm{e} ^{\pm } \rightarrow \mathrm{q} {\bar{\mathrm{q}}} \mathrm{q} {\bar{\mathrm{q}}} {\nu }$. The absolute likelihood *L* for each event type is formed from normalised probability distributions $P_i(x_i)$ of the *N* likelihood discriminating variables $x_i$ for that event type. For example, the distribution of the reconstructed Higgs mass for all events passing the preselection is shown in Fig. 15; it can be seen that good separation between signal and background is achievable. The discriminating variables are: the 2D distribution of reconstructed invariant masses $m_{{\mathrm{H}}}$ and $m_{{\mathrm{W}}}$, the 2D distribution of minimal $k_t$ distances $y_{23}$, $y_{34}$, and the 2D distribution of ${\mathrm{b}}$-tag probabilities when the event is forced into two jets. The use of 2D distributions accounts for the most significant correlations between the likelihood variables. The selection efficiencies and expected numbers of events for the signal dominated region, $\mathcal{{L}}>0.35$, are listed in Table 20.

The expected precision on $BR ({\mathrm{H}} \rightarrow {\mathrm{W}} {\mathrm{W}} ^*)$ is extracted from a fit to the likelihood distribution. Given the non-negligible backgrounds from other Higgs decays, it is necessary to simultaneously fit the different components. A $\chi ^2$ fit to the expected $\mathcal{{L}}$ distribution is performed by scaling independently five components: the ${\mathrm{H}} \rightarrow {\mathrm{W}} {\mathrm{W}} ^*$ signal, the ${\mathrm{H}} \rightarrow {\mathrm{b}} {\bar{\mathrm{b}}}$, ${\mathrm{H}} \rightarrow {\mathrm{c}} {\bar{{\mathrm{c}}}}$ and ${\mathrm{H}} \rightarrow {\mathrm{g}} {\mathrm{g}}$ backgrounds, and all other backgrounds (dominated by $\mathrm{q} {\bar{\mathrm{q}}} {\nu } \bar{\nu }$ and $\mathrm{q} {\bar{\mathrm{q}}} \mathrm{q} {\bar{\mathrm{q}}} {\nu }$). The constraints on the ${\mathrm{H}} \rightarrow {\mathrm{b}} {\bar{\mathrm{b}}}$, ${\mathrm{H}} \rightarrow {\mathrm{c}} {\bar{{\mathrm{c}}}}$ and ${\mathrm{H}} \rightarrow {\mathrm{g}} {\mathrm{g}}$ branching ratios, as described in Sect. 6.1, are implemented by modifying the $\chi ^2$ function to include penalty terms:

$$\begin{aligned} \chi ^2 \rightarrow&\chi ^2 + \frac{(s_{{\mathrm{b}} {\bar{\mathrm{b}}}}-1)^2}{\sigma ^2_{{\mathrm{b}} {\bar{\mathrm{b}}}}} + \frac{(s_{{\mathrm{c}} {\bar{{\mathrm{c}}}}}-1)^2}{\sigma ^2_{{\mathrm{c}} {\bar{{\mathrm{c}}}}}} + \frac{(s_{{\mathrm{g}} {\mathrm{g}}}-1)^2}{\sigma ^2_{{\mathrm{g}} {\mathrm{g}}}} \\&+\,\frac{(s_{{\mathrm{Z}} {\mathrm{Z}} ^*}-1)^2}{\sigma ^2_{{\mathrm{Z}} {\mathrm{Z}} ^*}} + \frac{(b-1)^2}{\sigma ^2_b} . \end{aligned}$$

Here, for example, $s_{{\mathrm{g}} {\mathrm{g}}}$ is the amount by which the ${\mathrm{H}} \rightarrow {\mathrm{g}} {\mathrm{g}}$ complement is scaled in the fit and $\sigma _{{\mathrm{g}} {\mathrm{g}}}$ is the expected statistical error on $BR ({\mathrm{H}} \rightarrow {\mathrm{g}} {\mathrm{g}})$ from the analysis of Sect. 6.1. The expected uncertainties on the contributions from ${\mathrm{H}} \rightarrow {\mathrm{b}} {\bar{\mathrm{b}}}$ and ${\mathrm{H}} \rightarrow {\mathrm{c}} {\bar{{\mathrm{c}}}}$ are taken from the same analysis. The background from ${\mathrm{H}} \rightarrow {\mathrm{Z}} {\mathrm{Z}} ^*$ is assumed here to be known to 1% from other measurements of $g_{{\mathrm{H}} {\mathrm{Z}} {\mathrm{Z}}} ^2$ and $g_{{\mathrm{H}} {\mathrm{Z}} {\mathrm{Z}}} ^2/g_{{\mathrm{H}} {\mathrm{W}} {\mathrm{W}}} ^2$. The systematic uncertainty in the non-${\mathrm{H}}$ background, denoted by *b*, is taken to be 1%. This has a small effect on the resulting uncertainty on the ${\mathrm{H}} \rightarrow {\mathrm{W}} {\mathrm{W}} ^*$ branching ratio, which is:

$$\begin{aligned} \frac{\varDelta [\sigma ({\mathrm{H}} {{\nu }}_{\!\mathrm{e}} {\bar{{\nu }}}_{\!\mathrm{e}} )\times BR ({\mathrm{H}} \rightarrow {\mathrm{W}} {\mathrm{W}} ^*)]}{\sigma ({\mathrm{H}} {{\nu }}_{\!\mathrm{e}} {\bar{{\nu }}}_{\!\mathrm{e}} )\times BR ({\mathrm{H}} \rightarrow {\mathrm{W}} {\mathrm{W}} ^*)} = 1.5\% . \end{aligned}$$

#### ${\mathrm{W}} {\mathrm{W}} ^*\rightarrow \mathrm{q} {\bar{\mathrm{q}}} \mathrm{l} {\nu }$

As a second channel, the ${\mathrm{H}} \rightarrow {\mathrm{W}} {\mathrm{W}} ^*\rightarrow \mathrm{q}{\bar{\mathrm{q}}} \mathrm{l} {\nu }$ decay is investigated [56]. The study is performed at $\sqrt{s} =1.4\,\text {TeV}$ using the CLIC_ILD detector model.

As a first step, isolated electrons or muons from W boson decay are identified. An efficiency of 93% is achieved for the identification of electrons and muons in signal events including the geometrical acceptance of the detector. Two jets are reconstructed from the remaining particles, excluding the isolated electron or muon. Flavour tagging information is obtained from the LcfiPlus package.

The following preselection cuts are imposed:

- energy of the W candidate less than 590 $\text {GeV}$;
- mass of the W candidate less than 230 $\text {GeV}$;
- energy of the H candidate less than 310 $\text {GeV}$;
- total missing energy of the event in the range between 670 $\text {GeV}$ and 1.4 $\text {TeV}$.

Nearly all signal events pass this preselection, while more than 30% of the critical ${\mathrm{e}^{+}}{\mathrm{e}^{-}} \rightarrow \mathrm{q}{\bar{\mathrm{q}}} \mathrm{l} {\nu }$ background events are rejected. The background processes are suppressed further using a BDT classifier with 19 input variables including the number of isolated leptons. The event selection is summarised in Table 21. The resulting statistical precision for $1.5\,\text {ab}^{-1}$ is:

$$\begin{aligned} \frac{\varDelta [\sigma ({\mathrm{H}} {{\nu }}_{\!\mathrm{e}} {\bar{{\nu }}}_{\!\mathrm{e}} )\times BR ({\mathrm{H}} \rightarrow {\mathrm{W}} {\mathrm{W}} ^*)]}{\sigma ({\mathrm{H}} {{\nu }}_{\!\mathrm{e}} {\bar{{\nu }}}_{\!\mathrm{e}} )\times BR ({\mathrm{H}} \rightarrow {\mathrm{W}} {\mathrm{W}} ^*)} = 1.3\% . \end{aligned}$$

The combined precision for ${\mathrm{H}} \rightarrow {\mathrm{W}} {\mathrm{W}} ^*\rightarrow \mathrm{q} {\bar{\mathrm{q}}} \mathrm{q} {\bar{\mathrm{q}}}$ and ${\mathrm{H}} \rightarrow {\mathrm{W}} {\mathrm{W}} ^*\rightarrow \mathrm{q}{\bar{\mathrm{q}}} \mathrm{l} {\nu }$ decays at $\sqrt{s} =1.4\,\text {TeV}$ for an integrated luminosity of $1.5\,\text {ab}^{-1}$ is 1.0%.

**Table 21:** Preselection and selection efficiencies for the signal and most important background processes in the ${\mathrm{H}} \rightarrow {\mathrm{W}} {\mathrm{W}} ^*\rightarrow \mathrm{q} {\bar{\mathrm{q}}} \mathrm{l} {\nu }$ analysis. Numbers of events correspond to 1.5 $\text {ab}^{-1}$ at $\sqrt{s} =1.4\,\text {TeV}$

Process	$\sigma /\text {fb}$	$\varepsilon _\text {presel}$ (%)	$\varepsilon _\text {BDT}$ (%)	$N_\text {BDT}$
${\mathrm{e}^{+}}{\mathrm{e}^{-}} \rightarrow {\mathrm{H}} {{\nu }}_{\!\mathrm{e}} {\bar{{\nu }}}_{\!\mathrm{e}} ;$	18.9	100	42	11,900
$${\mathrm{H}} \rightarrow {\mathrm{W}} {\mathrm{W}} ^*\rightarrow \mathrm{q} {\bar{\mathrm{q}}} \mathrm{l} {\nu } $$				
${\mathrm{e}^{+}}{\mathrm{e}^{-}} \rightarrow {\mathrm{H}} {{\nu }}_{\!\mathrm{e}} {\bar{{\nu }}}_{\!\mathrm{e}} ;$	25.6	100	1.9	721
$${\mathrm{H}} \rightarrow {\mathrm{W}} {\mathrm{W}} ^*\rightarrow \mathrm{q} {\bar{\mathrm{q}}} \mathrm{q} {\bar{\mathrm{q}}} $$				
${\mathrm{e}^{+}}{\mathrm{e}^{-}} \rightarrow {\mathrm{H}} {{\nu }}_{\!\mathrm{e}} {\bar{{\nu }}}_{\!\mathrm{e}} ;$	200	99.6	1.2	3660
$${\mathrm{H}} \rightarrow other $$				
${\mathrm{e}^{+}}{\mathrm{e}^{-}} \rightarrow \mathrm{q} {\bar{\mathrm{q}}} {\nu } \bar{\nu }$	788	97	0.07	841
${\mathrm{e}^{+}}{\mathrm{e}^{-}} \rightarrow \mathrm{q} {\bar{\mathrm{q}}} \mathrm{l} \mathrm{l}$	2730	90	0.005	178
${\mathrm{e}^{+}}{\mathrm{e}^{-}} \rightarrow \mathrm{q} {\bar{\mathrm{q}}} \mathrm{l} {\nu }$	4310	67	0.11	4730
${\upgamma } \mathrm{e} ^{\pm } \rightarrow \mathrm{q} {\bar{\mathrm{q}}} \mathrm{e} ^{\pm }$	88,400	86	0.0013	1430

### ${\mathrm{H}} \rightarrow {\mathrm{Z}} {\mathrm{Z}} ^*$

The decay ${\mathrm{H}} \rightarrow {\mathrm{Z}} {{\mathrm{Z}}}^{*}$ in ${\mathrm{e}^{+}}{\mathrm{e}^{-}} \rightarrow {\mathrm{H}} {{\nu }}_{\!\mathrm{e}} {\bar{{\nu }}}_{\!\mathrm{e}}$ events is studied using ${\mathrm{Z}} ^{(*)}\rightarrow \mathrm{q}{\bar{\mathrm{q}}}$ and ${\mathrm{Z}} ^{(*)}\rightarrow {\mathrm{l}}^{+} {\mathrm{l}}^{-}$ decays at $\sqrt{s} = 1.4\,\text {TeV}$ using the CLIC_ILD detector model. The experimental signature is two jets, a pair of oppositely charged leptons and missing $p_\mathrm {T}$. The total invariant mass of all visible final-state particles is equal to the Higgs mass, while either the quarks or the charged lepton pair have a mass consistent with $m_{{\mathrm{Z}}}$. Due to the large background from ${\mathrm{H}} \rightarrow {\mathrm{W}} {\mathrm{W}} ^*$, the ${\mathrm{Z}} {{\mathrm{Z}}}^{*} \rightarrow \mathrm{q} {\bar{\mathrm{q}}} \mathrm{q} {\bar{\mathrm{q}}}$ final state is not considered here. The ${\mathrm{Z}} {{\mathrm{Z}}}^{*} \rightarrow {\mathrm{l}}^{+} {\mathrm{l}}^{-} {\mathrm{l}}^{+} {\mathrm{l}}^{-}$ signature is not expected to be competitive at CLIC due to the small number of expected events and is not further considered.

**Table 22:** Preselection and selection efficiencies for the signal and the relevant background processes in the ${\mathrm{H}} \rightarrow {\mathrm{Z}} {{\mathrm{Z}}}^{*}$ analysis. The numbers of events correspond to 1.5 $\text {ab}^{-1}$ at $\sqrt{s} =1.4\,\text {TeV}$. All background processes other than Higgs production are completely rejected by the event selection

Process	$\sigma /\text {fb}$	$\varepsilon _\text {presel}$ (%)	$\varepsilon _\text {BDT}$ (%)	$N_\text {BDT}$
${\mathrm{e}^{+}}{\mathrm{e}^{-}} \rightarrow {\mathrm{H}} {{\nu }}_{\!\mathrm{e}} {\bar{{\nu }}}_{\!\mathrm{e}} ;$	0.995	62	46	425
$${\mathrm{H}} \rightarrow {\mathrm{Z}} {{\mathrm{Z}}}^{*} \rightarrow \mathrm{q}{\bar{\mathrm{q}}} {\mathrm{l}}^{+} {\mathrm{l}}^{-} $$				
${\mathrm{e}^{+}}{\mathrm{e}^{-}} \rightarrow {\mathrm{H}} {{\nu }}_{\!\mathrm{e}} {\bar{{\nu }}}_{\!\mathrm{e}} ;$	25.6	32	0.2	24
$$ {\mathrm{H}} \rightarrow {\mathrm{W}} {\mathrm{W}} ^{*}\rightarrow \mathrm{q}{\bar{\mathrm{q}}} \mathrm{q}{\bar{\mathrm{q}}} $$				
${\mathrm{e}^{+}}{\mathrm{e}^{-}} \rightarrow {\mathrm{H}} {{\nu }}_{\!\mathrm{e}} {\bar{{\nu }}}_{\!\mathrm{e}} ;{\mathrm{H}} \rightarrow {\mathrm{b}} {\bar{\mathrm{b}}}$	137	20	0.06	23
${\mathrm{e}^{+}}{\mathrm{e}^{-}} \rightarrow {\mathrm{H}} {{\nu }}_{\!\mathrm{e}} {\bar{{\nu }}}_{\!\mathrm{e}} ;{\mathrm{H}} \rightarrow {\mathrm{g}} {\mathrm{g}}$	21	25	0.05	4
${\mathrm{e}^{+}}{\mathrm{e}^{-}} \rightarrow {\mathrm{H}} {{\nu }}_{\!\mathrm{e}} {\bar{{\nu }}}_{\!\mathrm{e}} ;{\mathrm{H}} \rightarrow {\mathrm{c}} {\bar{{\mathrm{c}}}}$	6.9	23	0.0	0
${\mathrm{e}^{+}}{\mathrm{e}^{-}} \rightarrow {\mathrm{H}} {{\nu }}_{\!\mathrm{e}} {\bar{{\nu }}}_{\!\mathrm{e}} ;{\mathrm{H}} \rightarrow other$	51	50	0.3	98

**Fig. 16:**
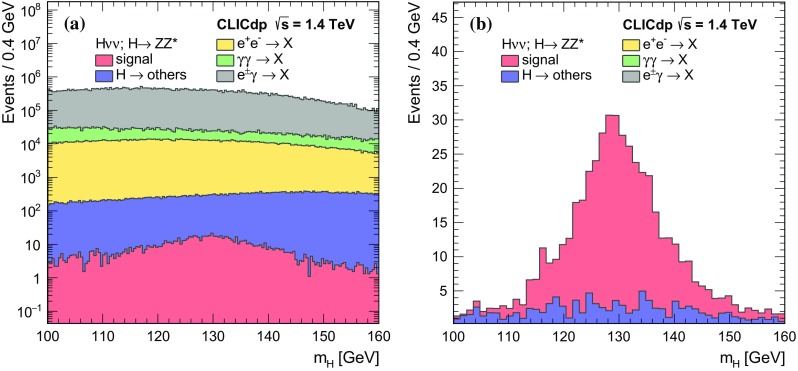
Reconstructed Higgs invariant mass distributions of ${\mathrm{H}} \rightarrow {\mathrm{Z}} {{\mathrm{Z}}}^{*} \rightarrow \mathrm{q}{\bar{\mathrm{q}}} {\mathrm{l}}^{+} {\mathrm{l}}^{-}$ events at $\sqrt{s} = 1.4\,\text {TeV}$, showing the signal and main backgrounds as stacked histograms **a** after preselection, and **b** after the full event selection including a cut on the BDT classifier. The distributions are normalised to an integrated luminosity of $1.5\,\text {ab}^{-1}$

The analysis is performed in several steps. First, isolated electrons and muons with an impact parameter of less than 0.02 mm are searched for. Hadronic $\uptau$ lepton decays are identified using the TauFinder algorithm described in Sect. 5.2.2, with the requirement $p_\mathrm {T} > 10\,\text {GeV}$ for the seed track and $p_\mathrm {T} > 4\,\text {GeV}$ for all other tracks within a search cone of 0.15 radian. In signal events, 87% of the electron or muon pairs and 37% of the tau lepton pairs are found, including the effect of the geometrical acceptance of the detector in the forward direction.

In events with exactly two identified leptons of the same flavour and opposite charge, two jets are reconstructed from the remaining particles. No other preselection cuts are applied. Flavour tagging information is obtained from the LcfiPlus package.

A BDT classifier is used to suppress the background processes using 17 input variables, including:

- the invariant masses of the H, Z and ${{\mathrm{Z}}}^{*}$candidates;
- the topology of the hadronic system: $-\log _{10}(y_{34})$, $-\log _{10}(y_{23})$ and $-\log _{10}(y_{12})$;
- the b-tag and c-tag probabilities for both jets;
- the visible energy and the missing transverse momentum of the event;
- the number of particles in the event.

The event selection is summarised in Table 22. Only backgrounds from other Higgs decays pass the event selection, while all other background processes are fully rejected. The invariant mass distribution of the Higgs candidates in events with two isolated leptons after the full selection chain, including the BDT classifier, is shown in Fig. 16. The resulting statistical uncertainty is:

$$\begin{aligned} \frac{\varDelta [\sigma ({\mathrm{H}} {{\nu }}_{\!\mathrm{e}} {\bar{{\nu }}}_{\!\mathrm{e}} )\times BR ({\mathrm{H}} \rightarrow {\mathrm{Z}} {\mathrm{Z}} ^*)]}{\sigma ({\mathrm{H}} {{\nu }}_{\!\mathrm{e}} {\bar{{\nu }}}_{\!\mathrm{e}} )\times BR ({\mathrm{H}} \rightarrow {\mathrm{Z}} {\mathrm{Z}} ^*)} = 5.6\% . \end{aligned}$$

### ${\mathrm{H}} \rightarrow {\upgamma } {\upgamma }$

The measurement of the ${\mathrm{H}} \rightarrow {\upgamma } {\upgamma }$ decay played a central role in the discovery of the Higgs boson at the LHC [1, 2]. In the SM, this decay is induced via loops of heavy charged particles, with dominant contributions from W bosons and t quarks. For BSM scenarios, other heavy charged particles can appear in the loops, modifying the expected effective ${\mathrm{H}} \rightarrow {\upgamma } {\upgamma }$ branching ratio. The sensitivity for the measurement of $BR ({\mathrm{H}} \rightarrow {\upgamma } {\upgamma } )$ at CLIC has been studied using the CLIC_SiD detector model for $\sqrt{s} = 1.4\,\text {TeV}$ and an integrated luminosity of 1.5 $\text {ab}^{-1}$. The SM branching ratio for $m_{{\mathrm{H}}} =126\,\text {GeV}$ is 0.23% which results in approximately 840 signal events. The experimental signature for ${\mathrm{e}^{+}}{\mathrm{e}^{-}} \rightarrow {\mathrm{H}} {{\nu }}_{\!\mathrm{e}} {\bar{{\nu }}}_{\!\mathrm{e}} ;$ ${\mathrm{H}} \rightarrow {\upgamma } {\upgamma }$ is two high $p_\mathrm {T}$ photons with invariant mass $m({\upgamma } {\upgamma })$ consistent with $m_{{\mathrm{H}}}$, and missing momentum from the ${{\nu }}_{\!\mathrm{e}} {\bar{{\nu }}}_{\!\mathrm{e}}$ system. All relevant SM background processes with one or two photons in the final state have been considered. In addition to the photons from the hard interaction, the MC samples include additional ISR and FSR photons.

**Table 23:** Signal and relevant background processes used in the ${\mathrm{H}} \rightarrow {\upgamma } {\upgamma }$ analysis. Additional photons from ISR and FSR are present in each sample. The cross sections for the backgrounds include cuts applied at generator level that are slightly looser than the preselection described in the text. The numbers of events correspond to 1.5 $\text {ab}^{-1}$ at $\sqrt{s} =1.4\,\text {TeV}$

Process	$\sigma /\text {fb}$	$\varepsilon _\text {presel}$ (%)	$\varepsilon _\text {BDT}$ (%)	$N_\text {BDT}$
${\mathrm{e}^{+}}{\mathrm{e}^{-}} \rightarrow {\mathrm{H}} {{\nu }}_{\!\mathrm{e}} {\bar{{\nu }}}_{\!\mathrm{e}} ;\,{\mathrm{H}} \rightarrow {\upgamma } {\upgamma }$	0.56	85	47	337
${\mathrm{e}^{+}}{\mathrm{e}^{-}} \rightarrow \nu \bar{\nu }{\upgamma }$	29.5	34	7.3	1110
${\mathrm{e}^{+}}{\mathrm{e}^{-}} \rightarrow \nu \bar{\nu }{\upgamma } {\upgamma }$	17.3	31	8.6	688
${\mathrm{e}^{+}}{\mathrm{e}^{-}} \rightarrow {\upgamma } {\upgamma }$	27.2	20	0.68	55
${\mathrm{e}^{+}}{\mathrm{e}^{-}} \rightarrow {\mathrm{e}^{+}}{\mathrm{e}^{-}} {\upgamma }$	289	9.2	0.66	265
${\mathrm{e}^{+}}{\mathrm{e}^{-}} \rightarrow {\mathrm{e}^{+}}{\mathrm{e}^{-}} {\upgamma } {\upgamma }$	12.6	5.2	0.2	2
${\mathrm{e}^{+}}{\mathrm{e}^{-}} \rightarrow \mathrm{q}{\bar{\mathrm{q}}} {\upgamma }$	67.0	0.8	0.0	0
${\mathrm{e}^{+}}{\mathrm{e}^{-}} \rightarrow \mathrm{q}{\bar{\mathrm{q}}} {\upgamma } {\upgamma }$	16.6	1.4	0.57	2

**Fig. 17:**
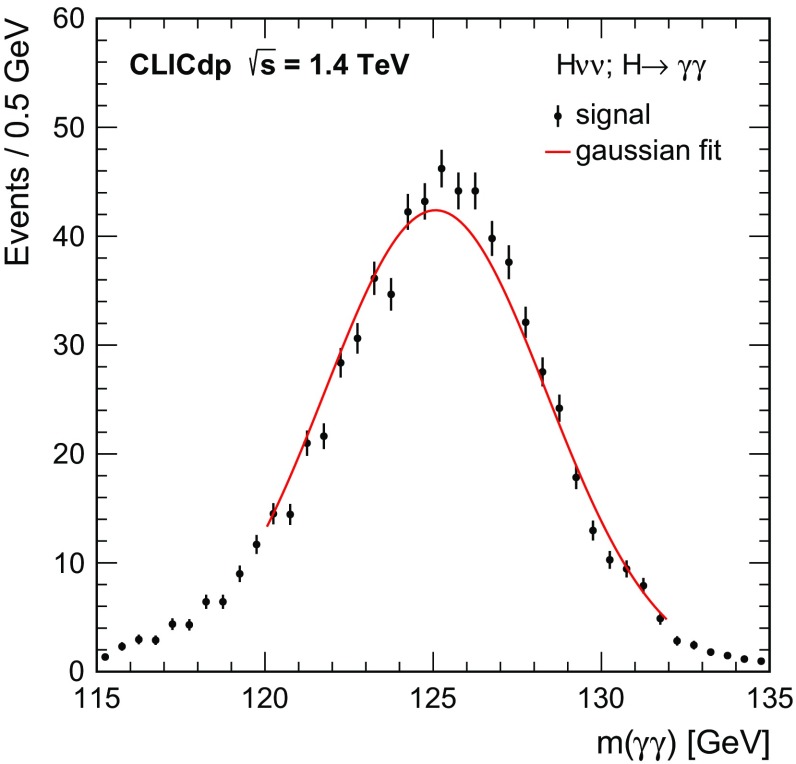
Reconstructed di-photon invariant mass distribution of preselected signal ${\mathrm{H}} \rightarrow {\upgamma } {\upgamma }$ events at $\sqrt{s} =1.4\,\text {TeV}$. The distribution is normalised to an integrated luminosity of $1.5\,\text {ab}^{-1}$. The statistical uncertainties correspond to the size of the simulated event sample. The *line* shows the fit described in the text

The following preselection cuts are applied to restrict the analysis to relevant events. At least two reconstructed photons each with energy $E_{{\upgamma }} > 15\,\text {GeV}$ and $p_\mathrm {T} > 10\,\text {GeV}$ are required. The two highest energy photons passing these requirements are used to form the ${\mathrm{H}}$ candidate and the preselection requires an invariant mass consistent with $m_{{\mathrm{H}}}$, $115\,\text {GeV}< m({\upgamma } {\upgamma } ) < 140\,\text {GeV}$. The highest energy photon in the event is required to have $p_\mathrm {T} >40\,\text {GeV}$. In addition, to remove contributions from FSR, both photons are required to be isolated with no reconstructed particle with $p_\mathrm {T} > 5\,\text {GeV}$ within a cone of radius 500 $\text {mrad}$ centred on the photon. Furthermore, the remaining reconstructed energy after excluding the Higgs candidate has to be less than 250 $\text {GeV}$. The cross sections and efficiencies of the preselection cuts for the signal and the main backgrounds are listed in Table 23. At this stage in the event selection the background dominates.

To illustrate the photon reconstruction capabilities of the CLIC_SiD detector concept, the invariant mass of Higgs candidates in signal events after the preselection is shown in Fig. 17. A fit to the distribution using a Gaussian function indicates a mass resolution in the signal sample of $\sigma = 3.3\,\text {GeV}$.

The signal and background events are classified using a BDT. The 13 variables used to distinguish the signal from the backgrounds include:

- the invariant mass of the Higgs candidate;
- kinematic properties of the Higgs candidate;
- kinematic properties of the two photons;
- the angle between the two photons and the helicity angle of the Higgs candidate;
- the remaining reconstructed energy excluding the Higgs candidate.

For the optimal BDT cut, the total signal selection efficiency is 40%, corresponding to 337 selected signal events in 1.5 $\text {ab}^{-1}$. The event selection for the signal and the main backgrounds is summarised in Table 23, leading to a statistical uncertainty of:

$$\begin{aligned} \frac{\varDelta [\sigma ({\mathrm{H}} {{\nu }}_{\!\mathrm{e}} {\bar{{\nu }}}_{\!\mathrm{e}} )\times BR ({\mathrm{H}} \rightarrow {\upgamma } {\upgamma })]}{\sigma ({\mathrm{H}} {{\nu }}_{\!\mathrm{e}} {\bar{{\nu }}}_{\!\mathrm{e}} )\times BR ({\mathrm{H}} \rightarrow {\upgamma } {\upgamma })} = 15\%. \end{aligned}$$

### ${\mathrm{H}} \rightarrow {\mathrm{Z}} {\upgamma }$

**Fig. 18:**
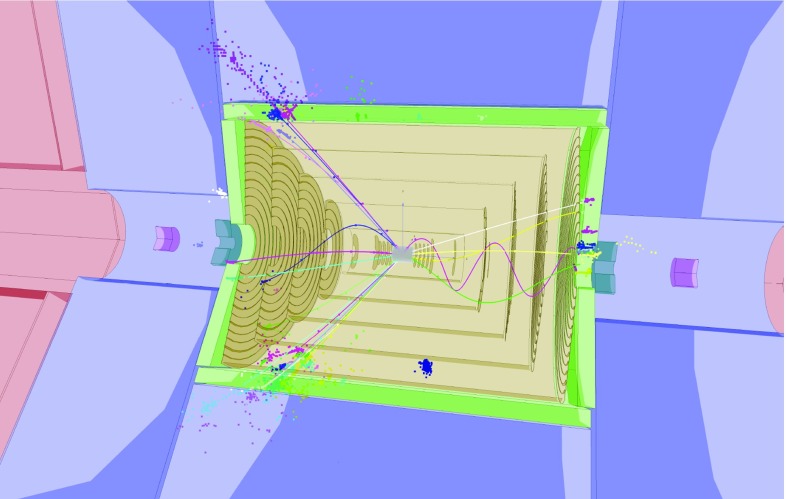
Event display of a ${\mathrm{H}} \rightarrow {\mathrm{Z}} {\upgamma } \rightarrow \mathrm{q} {\bar{\mathrm{q}}} {\upgamma }$ event at $\sqrt{s} =1.4\,\text {TeV}$ in the CLIC_SiD detector. Both jets are visible. The photon creates a cluster in the central part of the electromagnetic calorimeter (*blue*)

As is the case for ${\mathrm{H}} \rightarrow {\upgamma } {\upgamma }$, at lowest order, the SM decay ${\mathrm{H}} \rightarrow {\mathrm{Z}} {\upgamma }$ is induced by loops of heavy charged particles. Contributions from BSM particles would lead to deviations from the SM expectation for $BR ({\mathrm{H}} \rightarrow {\mathrm{Z}} {\upgamma })$. For $m_{{\mathrm{H}}} =126\,\text {GeV}$, the decay ${\mathrm{H}} \rightarrow {\mathrm{Z}} {\upgamma }$ is expected to have a branching ratio of $BR ({\mathrm{H}} \rightarrow {\mathrm{Z}} {\upgamma })= 0.16\%$. The potential to measure $\sigma ({\mathrm{e}^{+}}{\mathrm{e}^{-}} \rightarrow {\mathrm{H}} {{\nu }}_{\!\mathrm{e}} {\bar{{\nu }}}_{\!\mathrm{e}} ) \times BR ({\mathrm{H}} \rightarrow {\mathrm{Z}} {\upgamma })$ at CLIC has been studied at $\sqrt{s} =1.4\,\text {TeV}$ with the CLIC_SiD detector model, where 585 ${\mathrm{H}} \rightarrow {\mathrm{Z}} {\upgamma }$ events are expected in 1.5 $\text {ab}^{-1}$ of data [57]. For the purpose of the event selection, only ${\mathrm{Z}} \rightarrow \mathrm{q} {\bar{\mathrm{q}}}$ and ${\mathrm{Z}} \rightarrow {\mathrm{l}}^{+} {\mathrm{l}}^{-}$ (with $\mathrm{l} = \mathrm{e}, {\upmu }$) are useful, giving small event samples of 409 $\mathrm{q}{\bar{\mathrm{q}}} {\upgamma }$, 21 ${\mathrm{e}^{+}}{\mathrm{e}^{-}} {\upgamma }$ and 21 ${{{{\upmu }}^{+} {{\upmu }}^{-}}}{\upgamma }$ events from ${\mathrm{H}} \rightarrow {\mathrm{Z}} {\upgamma }$ in 1.5 $\text {ab}^{-1}$ at $\sqrt{s} =1.4\,\text {TeV}$. A typical event display is shown in Fig. 18.

The visible final states of the signal channels $\mathrm{q}{\bar{\mathrm{q}}} {\upgamma }$ or ${\mathrm{l}}^{+} {\mathrm{l}}^{-} {\upgamma }$ are also produced in several background processes, some of which have much larger cross sections than the signal. In addition to background with photons from the hard process, ${\mathrm{e}^{+}}{\mathrm{e}^{-}} \rightarrow \mathrm{q}{\bar{\mathrm{q}}}$ or ${\mathrm{e}^{+}}{\mathrm{e}^{-}} \rightarrow {\mathrm{l}}^{+} {\mathrm{l}}^{-}$ events with a FSR or ISR photon can mimic the signal.

The ${\mathrm{H}} \rightarrow {\mathrm{Z}} {\upgamma }$ event selection requires at least one identified high-$p_\mathrm {T}$ photon and either two electrons, muons or quarks consistent with a ${\mathrm{Z}}$ decay. The photon with the highest energy in the event is identified. Events are considered as either ${\mathrm{e}^{+}}{\mathrm{e}^{-}} {\upgamma }$, ${{{{\upmu }}^{+} {{\upmu }}^{-}}}{\upgamma }$ or $\mathrm{q}{\bar{\mathrm{q}}} {\upgamma }$ candidates. In the case where an ${\mathrm{e}^{+}}{\mathrm{e}^{-}}$ or ${{{{\upmu }}^{+} {{\upmu }}^{-}}}$ pair is found, photons nearly collinear with the lepton trajectories (within $0.3^\circ$) are combined with the leptons under the assumption that these photons originate from bremsstrahlung. If neither an ${\mathrm{e}^{+}}{\mathrm{e}^{-}}$ nor a ${{{{\upmu }}^{+} {{\upmu }}^{-}}}$ pair is found, all reconstructed particles except for the photon of highest energy are clustered into two jets using a jet radius of $R=1.2$. In all cases, the selected ${\mathrm{Z}}$ decay candidate and the highest energy photon are combined to form the ${\mathrm{H}}$ candidate.

In order to reduce the number of background process events, two selection steps are performed. First, preselection cuts are applied: the Higgs candidate daughter photon and jets, electrons, or muons are only accepted if they have an energy of $E > 20\,\text {GeV}$ and $p_\mathrm {T} >15\,\text {GeV}$. In the $\mathrm{q}{\bar{\mathrm{q}}} {\upgamma }$ channel, only jets with at least 5 particles are considered in order to suppress hadronic $\uptau$ decays. In addition, the reconstructed ${\mathrm{Z}}$ and ${\mathrm{H}}$ masses in the event are required to be consistent with a ${\mathrm{H}} \rightarrow {\mathrm{Z}} {\upgamma }$ decay. The second step in the event selection is three BDT selections (one for each signal final state). The input variables are the properties of the reconstructed ${\mathrm{H}}$, ${\mathrm{Z}}$, and ${\upgamma }$ such as mass, energy, momentum, and polar angle, event shapes such as sphericity and aplanarity, as well as missing energy distributions and particle multiplicity distributions.

For the optimal BDT cuts, expected statistical significances of 2.2, 0.54 and 0.78 (in units of standard deviations) are found for the $\mathrm{q}{\bar{\mathrm{q}}} {\upgamma }$, ${\mathrm{e}^{+}}{\mathrm{e}^{-}} {\upgamma }$ and ${{{{\upmu }}^{+} {{\upmu }}^{-}}}{\upgamma }$ channels respectively. The signal selection efficiencies and contributions from the most important backgrounds are summarised in Table 24. When the results from all three channels are combined, the expected statistical precision at $\sqrt{s} =1.4\,\text {TeV}$ for an integrated luminosity of 1.5 $\text {ab}^{-1}$ is:

$$\begin{aligned} \frac{\varDelta [\sigma ({\mathrm{H}} {{\nu }}_{\!\mathrm{e}} {\bar{{\nu }}}_{\!\mathrm{e}} )\times BR ({\mathrm{H}} \rightarrow {\mathrm{Z}} {\upgamma })]}{\sigma ({\mathrm{H}} {{\nu }}_{\!\mathrm{e}} {\bar{{\nu }}}_{\!\mathrm{e}} )\times BR ({\mathrm{H}} \rightarrow {\mathrm{Z}} {\upgamma })} = 42\%. \end{aligned}$$

With electron polarisation the statistical precision can be increased, for example with $-80\%$ electron polarisation, $\varDelta [ \sigma ({\mathrm{e}^{+}}{\mathrm{e}^{-}} \rightarrow {\mathrm{H}} {{\nu }}_{\!\mathrm{e}} {\bar{{\nu }}}_{\!\mathrm{e}} ) \times BR ({\mathrm{H}} \rightarrow {\mathrm{Z}} {\upgamma })] \approx 31\%$. Further gains are expected at higher centre-of-mass energies, as the Higgs production cross section at $\sqrt{s} =3\,\text {TeV}$ is 70% higher than at 1.4 $\text {TeV}$.

**Table 24:** Preselection and selection efficiencies for ${\mathrm{H}} \rightarrow {\mathrm{Z}} {\upgamma }$ events in all three considered Z decay channels. The cross sections for the backgrounds include kinematic cuts applied at generator level. All numbers assume an integrated luminosity of $1.5\,\text {ab}^{-1}$ at $1.4\text {TeV}$

Process	$\sigma /\text {fb}$	$\varepsilon _{\text {presel}}$ (%)	$\varepsilon _{\text {BDT}}$ (%)	$N_{\text {BDT}}$
${\mathrm{e}^{+}}{\mathrm{e}^{-}} \rightarrow {\mathrm{H}} {{\nu }}_{\!\mathrm{e}} {\bar{{\nu }}}_{\!\mathrm{e}} ;$	0.27	45	41	75
$${\mathrm{H}} \rightarrow {\mathrm{Z}} {\upgamma };\,{\mathrm{Z}} \rightarrow \mathrm{q}{\bar{\mathrm{q}}} $$				
${\mathrm{e}^{+}}{\mathrm{e}^{-}} \rightarrow \nu \bar{\nu }\mathrm{q}{\bar{\mathrm{q}}} {\upgamma }$	37.3	12	7.3	504
${\mathrm{e}^{+}}{\mathrm{e}^{-}} \rightarrow \nu \bar{\nu }\mathrm{q}{\bar{\mathrm{q}}}$	122	8.4	3.0	463
$\mathrm{e} ^{\pm } {\upgamma } \,\rightarrow \mathrm{e} ^{\pm } \mathrm{q}{\bar{\mathrm{q}}}$	978	2.4	0.2	70
${\mathrm{e}^{+}}{\mathrm{e}^{-}} \rightarrow {\mathrm{H}} {{\nu }}_{\!\mathrm{e}} {\bar{{\nu }}}_{\!\mathrm{e}} ;$	0.014	38	50	4
$${\mathrm{H}} \rightarrow {\mathrm{Z}} {\upgamma };\,{\mathrm{Z}} \rightarrow {\mathrm{e}^{+}}{\mathrm{e}^{-}} $$				
${\mathrm{e}^{+}}{\mathrm{e}^{-}} \rightarrow \nu \bar{\nu }{\mathrm{l}}^{+} {\mathrm{l}}^{-} {\upgamma }$	9.6	1.6	6.5	15
${\mathrm{e}^{+}}{\mathrm{e}^{-}} \rightarrow \nu \bar{\nu }{\mathrm{l}}^{+} {\mathrm{l}}^{-}$	23.3	1.0	34	12
$\mathrm{e} ^{\pm } {\upgamma } \, \rightarrow \mathrm{e} ^{\pm } {\mathrm{l}}^{+} {\mathrm{l}}^{-}$	1940	0.22	0.1	7
${\mathrm{e}^{+}}{\mathrm{e}^{-}} \rightarrow {\mathrm{H}} {{\nu }}_{\!\mathrm{e}} {\bar{{\nu }}}_{\!\mathrm{e}} ;$	0.014	54	44	5
$${\mathrm{H}} \rightarrow {\mathrm{Z}} {\upgamma };\,{\mathrm{Z}} \rightarrow {{{\upmu }}^{+} {{\upmu }}^{-}} $$				
${\mathrm{e}^{+}}{\mathrm{e}^{-}} \rightarrow \nu \bar{\nu }{\mathrm{l}}^{+} {\mathrm{l}}^{-} {\upgamma }$	9.6	1.2	8.1	14
${\mathrm{e}^{+}}{\mathrm{e}^{-}} \rightarrow \nu \bar{\nu }{\mathrm{l}}^{+} {\mathrm{l}}^{-}$	23.3	0.45	8.3	13
$\mathrm{e} ^{\pm } {\upgamma } \, \rightarrow \mathrm{e} ^{\pm } {\mathrm{l}}^{+} {\mathrm{l}}^{-}$	1940	0.27	1.1	9

### ${\mathrm{H}} \rightarrow {{{\upmu }}^{+} {{\upmu }}^{-}}$

The measurement of the rare ${\mathrm{H}} \rightarrow {{{\upmu }}^{+} {{\upmu }}^{-}}$ decay is challenging due to the very low SM branching ratio of $2\times 10^{-4}$. In ${\mathrm{e}^{+}}{\mathrm{e}^{-}} \rightarrow {\mathrm{H}} {{\nu }}_{\!\mathrm{e}} {\bar{{\nu }}}_{\!\mathrm{e}}$ production, the signature for ${\mathrm{H}} \rightarrow {{{\upmu }}^{+} {{\upmu }}^{-}}$ decay is a ${{{{\upmu }}^{+} {{\upmu }}^{-}}}$ pair with invariant mass consistent with $m_{{\mathrm{H}}}$ and missing momentum. The efficient rejection of background relies on the excellent detector momentum resolution, which directly influences the width of the reconstructed di-muon invariant mass peak. Signal and background events have been simulated at $\sqrt{s} =1.4\,\text {TeV}$ and $3\,\text {TeV}$ using the $\textsc {CLIC}\_\text {ILD}$ and $\textsc {CLIC}\_\text {SiD}$ detector models respectively [58, 59]. In contrast with other studies presented in this paper, an electron beam polarisation of $-80\%$ is assumed owing to the very small branching ratio for the ${\mathrm{H}} \rightarrow {{{\upmu }}^{+} {{\upmu }}^{-}}$ decay. The two analyses were performed independently. They follow the same strategy but differ in some of the observables that are used in the event selection.

**Table 25:** The signal and main backgrounds in the ${\mathrm{H}} \rightarrow {{{\upmu }}^{+} {{\upmu }}^{-}}$ analysis at $\sqrt{s} =1.4\,\text {TeV}$ with the corresponding cross sections. The numbers of selected events assume an integrated luminosity of $1.5\,\text {ab}^{-1}$ and $-80\%$ polarisation of the electron beam. Other processes, including ${\mathrm{e}^{+}}{\mathrm{e}^{-}} \rightarrow {{{\upmu }}^{+} {{\upmu }}^{-}}$ and $\mathrm{e} ^{\pm } {\upgamma } \rightarrow \mathrm{e} ^{\pm } {{{\upmu }}^{+} {{\upmu }}^{-}}$, contribute a total of less than 10 events to the final selection

Process	$\sigma /\text {fb}$	$\varepsilon _\text {presel}$ (%)	$\varepsilon _\text {BDT}$ (%)	$N_\text {BDT}$
${\mathrm{e}^{+}}{\mathrm{e}^{-}} \rightarrow {\mathrm{H}} {{\nu }}_{\!\mathrm{e}} {\bar{{\nu }}}_{\!\mathrm{e}} ;\,{\mathrm{H}} \rightarrow {{{\upmu }}^{+} {{\upmu }}^{-}}$	0.094	83	37	43
${\mathrm{e}^{+}}{\mathrm{e}^{-}} \rightarrow {{\nu }}_{\!\mathrm{e}} {\bar{{\nu }}}_{\!\mathrm{e}} {{{\upmu }}^{+} {{\upmu }}^{-}}$	232	1.1	27	1030
$\mathrm{e} ^{\pm } {\upgamma } \rightarrow \mathrm{e} ^{\pm } {{{\nu }}_{\upmu }} {{\bar{\nu }}_{\upmu }}{{{\upmu }}^{+} {{\upmu }}^{-}}$	35	8.5	1.3	57
${\upgamma } {\upgamma } \rightarrow {{{\nu }}_{\upmu }}{{\bar{\nu }}_{\upmu }}{{{\upmu }}^{+} {{\upmu }}^{-}}$	162	10.6	2.2	560

**Table 26:** The signal and most important background processes in the ${\mathrm{H}} \rightarrow {{{\upmu }}^{+} {{\upmu }}^{-}}$ analysis at $\sqrt{s} =3\,\text {TeV}$ with the corresponding cross sections. The numbers of selected events assume an integrated luminosity of $2\,\text {ab}^{-1}$ and $-80\%$ polarisation of the electron beam. All other processes contribute of the order of 10 events to the final event selection. The cross sections are calculated for events with invariant mass of the di-muon system between $100\,\text {GeV}$ and $140\,\text {GeV}$

Process	$\sigma /\text {fb}$	$\varepsilon _\text {presel}$ (%)	$\varepsilon _\text {BDT}$ (%)	$N_\text {BDT}$
${\mathrm{e}^{+}}{\mathrm{e}^{-}} \rightarrow {\mathrm{H}} {{\nu }}_{\!\mathrm{e}} {\bar{{\nu }}}_{\!\mathrm{e}}$; ${\mathrm{H}} \rightarrow {{{\upmu }}^{+} {{\upmu }}^{-}}$	0.16	64	41	84
${\mathrm{e}^{+}}{\mathrm{e}^{-}} \rightarrow {{\nu }}_{\!\mathrm{e}} {\bar{{\nu }}}_{\!\mathrm{e}} {{{\upmu }}^{+} {{\upmu }}^{-}}$	6.6	33	41	1797
$\mathrm{e} ^{\pm } {\upgamma } \rightarrow \mathrm{e} ^{\pm } {{{\upmu }}^{+} {{\upmu }}^{-}}$	1210	6.9	0.16	262
${\upgamma } {\upgamma } \rightarrow {{{\nu }}_{\upmu }}{{\bar{\nu }}_{\upmu }}{{{\upmu }}^{+} {{\upmu }}^{-}}$	413	4.3	0.50	176

The most important background processes include ${{{{\upmu }}^{+} {{\upmu }}^{-}}}\nu \bar{\nu }$ in the final state, as shown in Table 25 for $1.4\,\text {TeV}$ and in Table 26 for $3\,\text {TeV}$. A significant fraction of these events are also produced from interactions involving beamstrahlung photons. Another important background is ${\mathrm{e}^{+}}{\mathrm{e}^{-}} \rightarrow {\mathrm{e}^{+}}{\mathrm{e}^{-}} {{{\upmu }}^{+} {{\upmu }}^{-}}$, where both electrons are usually emitted at very low polar angles and thus might not be detected. Tagging of these low angle electrons in the very forward calorimeters – LumiCal and BeamCal – is essential to keep this background under control.

The event selection requires two reconstructed, oppositely charged muons with a di-muon invariant mass within the relevant mass region of $105-145\,\text {GeV}$. Events with one or more detected high-energy electrons ($E>200\,\text {GeV}$ at $1.4\,\text {TeV}$, $E>250\,\text {GeV}$ at $3\,\text {TeV}$) in the very forward calorimeters are vetoed. This introduces the possibility of vetoing signal events if they coincide with Bhabha scattering events. The ${\mathrm{e}^{+}}{\mathrm{e}^{-}} \rightarrow {\mathrm{e}^{+}}{\mathrm{e}^{-}}$ cross section is sufficiently high that the probability of such a coincidence within 20 bunch crossings ($10\,\text {ns}$) is about $7\%$ in both analyses. The cuts on the minimum energy and the minimum polar angle for vetoing forward electrons need to be chosen carefully. ${\mathrm{e}^{+}}{\mathrm{e}^{-}} \rightarrow {\mathrm{e}^{+}}{\mathrm{e}^{-}} {{{\upmu }}^{+} {{\upmu }}^{-}}$ and $\mathrm{e} ^{\pm } {\upgamma } \rightarrow \mathrm{e} ^{\pm } {{{\upmu }}^{+} {{\upmu }}^{-}}$ events need to be rejected efficiently while a low probability for coincidence with Bhabha scattering events needs to be maintained.

The $3\,\text {TeV}$ analysis includes some additional preselection cuts to remove phase space regions that do not include any signal events. These cuts reject events that contain a reconstructed non-muon object with an energy greater than $100\,\text {GeV}$; in addition, events containing electrons in the central region of the detector with an energy above $20\,\text {GeV}$ are also rejected. The sum of the transverse momenta of the two muons, $p_\mathrm {T} ({{\upmu }}^{-}) + p_\mathrm {T} ({{\upmu }}^{+})$, is required to be above $50\,\text {GeV}$ and the transverse momentum of the di-muon system should be above $25\,\text {GeV}$.

The final event selection uses a BDT classifier using various kinematic variables, excluding the invariant mass of the di-muon system. The $1.4\,\text {TeV}$ analysis uses the visible energy of the event after removal of the di-muon system $E_{\text {vis}}$, the transverse momentum of the di-muon system $p_\mathrm {T} ({\upmu } {\upmu })$, the sum of the transverse momenta of the two muons $p_\mathrm{T}({{\upmu }}^{-}) + p_\mathrm{T}({{\upmu }}^{+})$, the polar angle of the di-muon system $\theta _{{\upmu } {\upmu }}$, the boost of the di-muon system $\beta _{{\upmu } {\upmu }}$, and the cosine of the helicity angle $\cos {\theta ^{*}}$. The $3\,\text {TeV}$ analysis uses the energy of the hardest non-muon object instead of the total visible energy and also includes the energy, transverse momentum, polar angle and azimuthal angle of both individual muons. This event selection reduces background from four-fermion processes by several orders of magnitude, while maintaining an overall signal selection efficiency of $\epsilon = 30.5\%$ and $\epsilon = 26.3\%$ at 1.4 and $3\,\text {TeV}$ respectively.

**Fig. 19:**
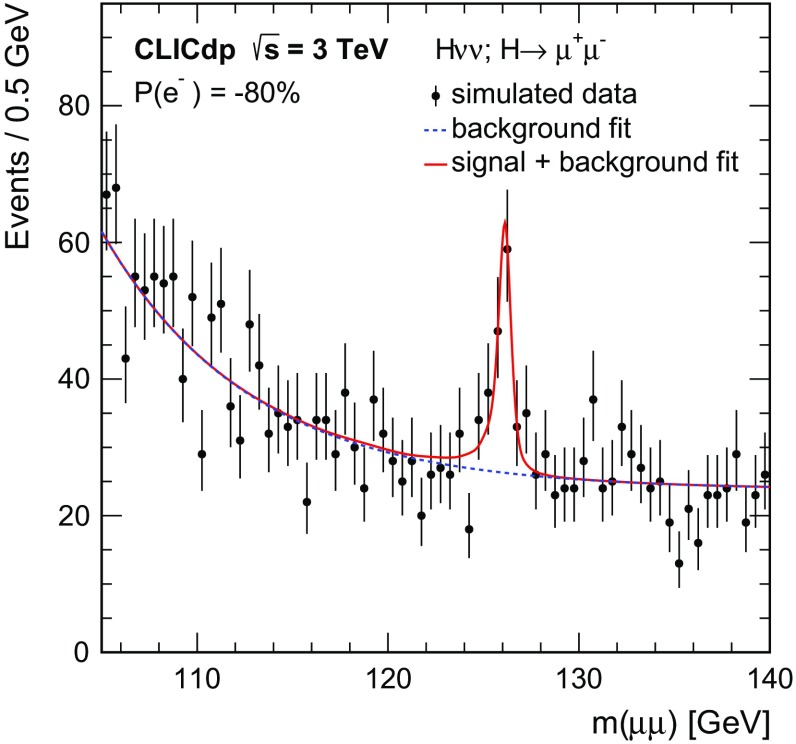
Reconstructed di-muon invariant mass distribution of selected ${\mathrm{H}} \rightarrow {{{\upmu }}^{+} {{\upmu }}^{-}}$ events at $\sqrt{s} =3\,\text {TeV}$. The simulated data are shown as *dots* while the *solid line* represents the fit function described in the text. The *dotted lin*e shows the background contribution of the fit function. The distribution is normalised to an integrated luminosity of $2\,\text {ab}^{-1}$, assuming $-80\%$ electron polarisation

The number of signal events is extracted from the reconstructed invariant mass distribution after the event selection, as shown in Fig. 19. Using a large MC sample, the signal and background shapes are extracted. The signal is described by a Gaussian distribution with asymmetric exponential tails. The combined background is parameterised as the sum of an exponential and a constant function. To assess the expected statistical precision, a large number of trial samples are generated from the expected reconstructed mass distributions of signal and background and are then fitted to the signal and background components. For $P(\mathrm{e}^-) = -80\%$, the expected relative uncertainty on the $\sigma ({\mathrm{e}^{+}}{\mathrm{e}^{-}} \rightarrow {\mathrm{H}} {{\nu }}_{\!\mathrm{e}} {\bar{{\nu }}}_{\!\mathrm{e}} ) \times BR ({\mathrm{H}} \rightarrow {{{\upmu }}^{+} {{\upmu }}^{-}})$ is $27\%$, corresponding to a significance of 3.7, at $1.4\,\text {TeV}$, and $19\%$, corresponding to a significance of 5.2, at $3\,\text {TeV}$. The corresponding uncertainties for unpolarised beams are:

$$\begin{aligned} \frac{\varDelta [\sigma ({\mathrm{H}} {{\nu }}_{\!\mathrm{e}} {\bar{{\nu }}}_{\!\mathrm{e}} )\times BR ({\mathrm{H}} \rightarrow {{{\upmu }}^{+} {{\upmu }}^{-}})]}{\sigma ({\mathrm{H}} {{\nu }}_{\!\mathrm{e}} {\bar{{\nu }}}_{\!\mathrm{e}} )\times BR ({\mathrm{H}} \rightarrow {{{\upmu }}^{+} {{\upmu }}^{-}})}&= 38\% \ \text {at} \ 1.4\,\text {TeV}, \\ \frac{\varDelta [\sigma ({\mathrm{H}} {{\nu }}_{\!\mathrm{e}} {\bar{{\nu }}}_{\!\mathrm{e}} )\times BR ({\mathrm{H}} \rightarrow {{{\upmu }}^{+} {{\upmu }}^{-}})]}{\sigma ({\mathrm{H}} {{\nu }}_{\!\mathrm{e}} {\bar{{\nu }}}_{\!\mathrm{e}} )\times BR ({\mathrm{H}} \rightarrow {{{\upmu }}^{+} {{\upmu }}^{-}})}&= 25\% \ \text {at} \ 3\,\text {TeV}. \end{aligned}$$

## ZZ-fusion

Higgs boson production through the *t*-channel fusion of two ${\mathrm{Z}}$ bosons, ${\mathrm{e}^{+}}{\mathrm{e}^{-}} \rightarrow {\mathrm{H}} {\mathrm{e}^{+}}{\mathrm{e}^{-}}$, is analogous to the ${\mathrm{W}} {\mathrm{W}}$-fusion process but gives access to $g_{{\mathrm{H}} {\mathrm{Z}} {\mathrm{Z}}}$ and $g_{{\mathrm{H}} {\mathrm{b}} {\mathrm{b}}}$ using a complementary technique. At $\sqrt{s} =1.4\,\text {TeV}$, ${\mathrm{Z}} {\mathrm{Z}}$-fusion is the sub-leading Higgs production process, with a cross section of around $25\,\text {fb}$, which is 10% of that for the ${\mathrm{W}} {\mathrm{W}}$-fusion process. The potential for the measurement of the ${\mathrm{Z}} {\mathrm{Z}}$-fusion process has been investigated at $\sqrt{s}=1.4\,\text {TeV}$ using the CLIC_ILD detector.

The characteristic signature of the ${\mathrm{Z}} {\mathrm{Z}}$-fusion process is two scattered beam electrons reconstructed in the forward regions of the detector, plus the Higgs boson decay products. Here, the scattered beam electrons are required to be fully reconstructed, and the final state ${\mathrm{H}} \rightarrow {\mathrm{b}} {\bar{\mathrm{b}}}$ is considered.

**Fig. 20:**
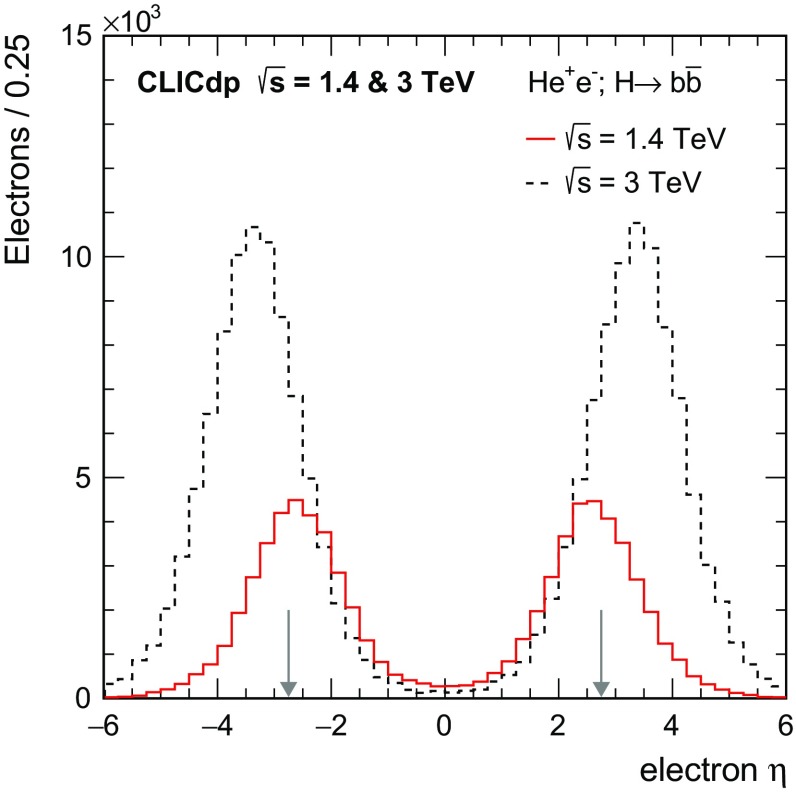
Generated electron pseudorapidity ($\eta =-\ln \tan \frac{\theta }{2}$) distributions for ${\mathrm{e}^{+}}{\mathrm{e}^{-}} \rightarrow {\mathrm{H}} {\mathrm{e}^{+}}{\mathrm{e}^{-}}$ events at $\sqrt{s} =1.4$ and $3\,\text {TeV}$. The distributions are normalised to 1.5 and $2\,\text {ab}^{-1}$ respectively. The *vertical arrows* show the detector acceptance

Events are clustered into a four-jet topology using a $k_{t}$ exclusive clustering algorithm with $R=1.0$. For a well-reconstructed signal event, two of the resulting ‘jets’ are expected to be the reconstructed electrons, and the remaining two jets originate from the Higgs decay to ${\mathrm{b}} {\bar{\mathrm{b}}}$. The event selection requires two oppositely-charged electron candidates, separated by $|\varDelta \eta | > 1$, each with $E>100\,\text {GeV}$. This preselection preserves 27% of the ${\mathrm{e}^{+}}{\mathrm{e}^{-}} \rightarrow {\mathrm{H}} {\mathrm{e}^{+}}{\mathrm{e}^{-}} \rightarrow {\mathrm{b}} {\bar{\mathrm{b}}} {\mathrm{e}^{+}}{\mathrm{e}^{-}}$ signal (3.6 fb), with the lost events almost entirely due to the scattered electrons falling outside the detector acceptance, as shown in Fig. 20. After the preselection, the SM background consists mainly of events that have two real electrons and a $\mathrm{q}{\bar{\mathrm{q}}}$ pair, either from the continuum or from the decay of ${\mathrm{Z}}$ bosons. Although the preselection suppresses 98% of the ${\mathrm{e}^{+}}{\mathrm{e}^{-}} \rightarrow \mathrm{q}{\bar{\mathrm{q}}} {\mathrm{e}^{+}}{\mathrm{e}^{-}}$ background, the accepted cross section is $48\,\text {fb}$, which is thirteen times larger than that for the remaining signal. A further requirement that one of the two jets associated with the Higgs decay has a ${\mathrm{b}}$-tag value $> 0.4$ preserves 80% of the remaining signal and rejects 80% of the remaining background.

A relative likelihood classifier $\mathscr {L}_1$, which treats ${\mathrm{Z}} {\mathrm{Z}}$-fusion events with ${\mathrm{H}} \rightarrow {\mathrm{b}} {\bar{\mathrm{b}}}$ as signal and ${\mathrm{H}} \rightarrow {\mathrm{W}} {\mathrm{W}} ^*$ and ${\mathrm{H}} \rightarrow {\mathrm{Z}} {\mathrm{Z}} ^*$ as background, is used to reduce contributions from other Higgs decays. Seven variables are used to construct the likelihood: the jet clustering variable $y_{45}$; the invariant mass of the two jets associated with the Higgs decay; the visible mass of the event with the scattered beam electrons removed; the higher of the ${\mathrm{b}}$-tag values of the two jets associated with the Higgs decay; the ${\mathrm{c}}$-tag value corresponding to the same jet; and the b-c-separation returned by the tagger, for both Higgs decay jets. Requiring a high signal likelihood, $\mathscr {L}_1 > 0.8$, reduces the ${\mathrm{H}} \rightarrow {\mathrm{b}} {\bar{\mathrm{b}}}$ signal to 3000 events but leaves only 90 events from other Higgs decays, while also reducing the non-Higgs backgrounds to 4700 events.

**Fig. 21:**
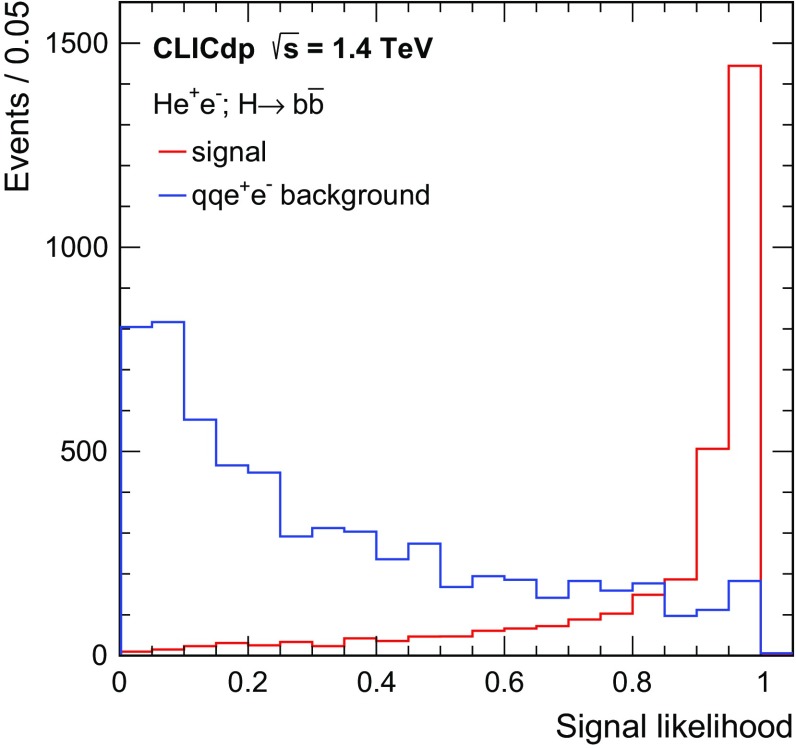
Likelihood distributions for ${\mathrm{H}} \rightarrow {\mathrm{b}} {\bar{\mathrm{b}}}$ events in the ZZ-fusion analysis at $\sqrt{s} =1.4\,\text {TeV}$, shown for the signal and main background. The distributions are normalised to an integrated luminosity of $1.5\,\text {ab}^{-1}$

Finally, to separate the signal from all backgrounds, a further relative likelihood classifier $\mathscr {L}_2$ is constructed using four variables that provide separation power between signal and background: the opening between the reconstructed electrons $\varDelta R$; the recoil mass of the event determined from the momenta of the reconstructed electrons, $m_\text {rec}$; the jet clustering variable $y_{34}$; and the invariant mass of the two jets associated with the Higgs decay.

The resulting likelihood is shown in Fig. 21 and gives good separation between signal and background. The likelihood distribution is fitted by signal and background components (where the normalisation is allowed to vary), giving:

$$\begin{aligned} \frac{\varDelta [\sigma ({\mathrm{H}} {\mathrm{e}^{+}}{\mathrm{e}^{-}})\times BR {({\mathrm{H}} \rightarrow {\mathrm{b}} {\bar{\mathrm{b}}})}]}{\sigma ({\mathrm{H}} {\mathrm{e}^{+}}{\mathrm{e}^{-}})\times BR {({\mathrm{H}} \rightarrow {\mathrm{b}} {\bar{\mathrm{b}}})}} = 1.8\% \end{aligned}$$

for $$1.5\,\text {ab}^{-1} $$ at $$\sqrt{s} =1.4\,\text {TeV} $$.

## Top Yukawa coupling

At an ${\mathrm{e}^{+}}{\mathrm{e}^{-}}$ collider the top Yukawa coupling, $y_{\mathrm{t}}$, can be determined from the production rate in the process where a Higgs boson is produced in association with a top quark pair, ${\mathrm{e}^{+}}{\mathrm{e}^{-}} \rightarrow \mathrm{t} {\bar{\mathrm{t}}} {\mathrm{H}}$. The top quarks decay almost exclusively by $\mathrm{t} \rightarrow {\mathrm{b}} {\mathrm{W}}$. The signal event topology thus depends on the nature of the ${\mathrm{W}}$ and Higgs boson decays. Here ${\mathrm{H}} \rightarrow {\mathrm{b}} {\bar{\mathrm{b}}}$ decays have been studied for two $\mathrm{t} {\bar{\mathrm{t}}} {\mathrm{H}}$ decay channels at $\sqrt{s} =1.4\,\text {TeV}$ using the CLIC_SiD detector model [60, 61]:

- the fully-hadronic channel (where both ${\mathrm{W}}$ bosons decay hadronically), giving a $\mathrm{t} {\bar{\mathrm{t}}} {\mathrm{H}}$ final state of eight jets, including four ${\mathrm{b}}$ jets;
- the semi-leptonic channel (where one ${\mathrm{W}}$ boson decays leptonically), giving a $\mathrm{t} {\bar{\mathrm{t}}} {\mathrm{H}}$ final state of six jets (four ${\mathrm{b}}$ jets), one lepton and one neutrino.

**Fig. 22:**
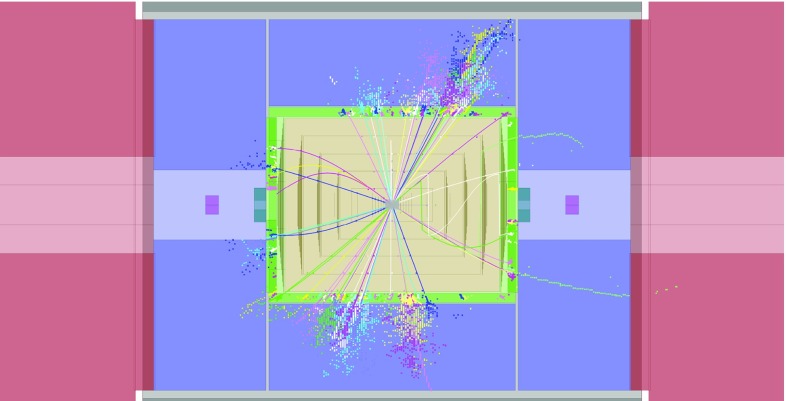
Event display of a $\mathrm{t} {\bar{\mathrm{t}}} {\mathrm{H}} \rightarrow {\mathrm{b}} {\bar{\mathrm{b}}} {\mathrm{b}} {\bar{\mathrm{b}}} \mathrm{q} {\bar{\mathrm{q}}} {\uptau }^{-} {\bar{{\nu }}}_{\uptau }$ event at $\sqrt{s} = 1.4\,\text {TeV}$ in the CLIC_SiD detector. The tau lepton decays hadronically

The two channels are distinguished by first searching for isolated leptons (muons and electrons with an energy of at least $15\,\text {GeV}$ and tau candidates from TauFinder containing a track with $p_\mathrm {T} > 10\,\text {GeV}$). If zero leptons are found, the event is classified as fully-hadronic. If one lepton is found, the event is classified as semi-leptonic. Events in which more than one lepton is found are not analysed further. The $k_{\mathrm {t}}$ algorithm is used to cluster the particles of each event into a specific number of jets, and remove particles arising from beam-beam interactions that are closer to the beam axis than to a hard jet as described in Sect. 4.2. Events classified as fully-hadronic are clustered into eight jets. In semi-leptonic events, the lepton is removed and the remaining particles are clustered into six jets. A semi-leptonic event is shown in Fig. 22. The particles not clustered into jets by the $k_{\mathrm {t}}$ algorithm are removed from the event and the remaining particles are then re-clustered using the ${\mathrm{e}^{+}}{\mathrm{e}^{-}}$ Durham algorithm in LcfiPlus, which performs flavour tagging for each jet, and prevents particles from displaced vertices being split between two or more jets. The jets are combined to form candidate primary particles in such a way so as to minimise a $\chi ^{2}$ function expressing the consistency of the reconstructed di- and tri-jet invariant masses with the $\mathrm{t} {\bar{\mathrm{t}}} ({\mathrm{H}} \rightarrow {\mathrm{b}} {\bar{\mathrm{b}}})$ hypothesis. For example, in the case of the semi-leptonic channel, the jet assignment with the minimum of:

$$\begin{aligned} \chi ^{2} = \frac{(m_{ij}-m_{{\mathrm{W}}})^{2}}{\sigma _{{\mathrm{W}}}^{2}} + \frac{(m_{ijk} - m_{\mathrm{t}})^{2}}{\sigma _{\mathrm{t}}^{2}} + \frac{(m_{lm} - m_{{\mathrm{H}}})^{2}}{\sigma _{{\mathrm{H}}}^{2}} , \end{aligned}$$

gives the W, top and Higgs candidates, where $m_{ij}$ is the invariant mass of the jet pair used to reconstruct the ${\mathrm{W}}$ candidate, $m_{ijk}$ is the invariant mass of the three jets used to reconstruct the top quark candidate and $m_{lm}$ is the invariant mass of the jet pair used to reconstruct the Higgs candidate. The expected invariant mass resolutions $\sigma _{{\mathrm{W}}, \mathrm{t}, {\mathrm{H}}}$ were estimated from combinations of two or three reconstructed jets matched to ${\mathrm{W}}$, top and Higgs particles on generator level.

**Fig. 23:**
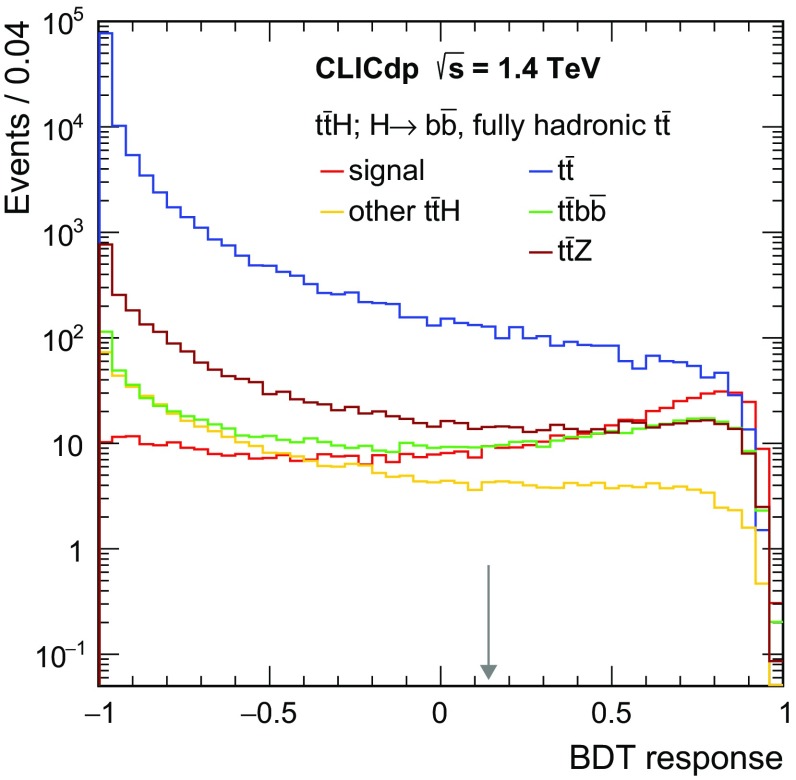
BDT classifier distributions for fully-hadronic $\mathrm{t} {\bar{\mathrm{t}}} {\mathrm{H}}$ events at $\sqrt{s} =1.4\,\text {TeV}$, shown for the $\mathrm{t} {\bar{\mathrm{t}}} {\mathrm{H}}$ signal and main backgrounds. The distributions are normalised to an integrated luminosity of $1.5\,\text {ab}^{-1}$. The *vertical arrow* shows the value of the cut, chosen to give the highest significance

Having forced each event into one of the two signal-like topologies, multivariate BDT classifiers (one for fully-hadronic events and one for semi-leptonic events) are used to separate signal and background. The discriminating variables include: kinematic quantities such as the reconstructed Higgs mass, the visible energy in the jets and the missing $p_\mathrm {T}$; angular variables such as the angles between the Higgs decay products in the rest frame of the Higgs candidate with respect to its flight direction and the angle between the momenta of the top and Higgs candidates; event variables such as thrust, sphericity and the number of particles in the event; and flavour tag variables for the four most likely b-jets. As an example, the BDT response distributions for the fully-hadronic channel are shown in Fig. 23. The selection is chosen to maximise the signal significance. The expected numbers of selected events for $1.5\,\text {ab}^{-1}$ of $\sqrt{s} =1.4\,\text {TeV}$ data are listed in Table 27. The contributions from other investigated background processes were found to be negligible. The $\mathrm{t} {\bar{\mathrm{t}}} {\mathrm{H}}$ cross section can be measured with an accuracy of $12\%$ in the semi-leptonic channel and $11\%$ in the hadronic channel. The combined precision of the two channels is $8\%$.

**Table 27:** Expected numbers of signal and background events in the fully-hadronic (HAD) and semi-leptonic (SL) channels for $1.5\,\text {ab}^{-1}$ at $\sqrt{s} =1.4\,\text {TeV}$. The columns show the total numbers of events before selection and the numbers of events passing the fully-hadronic and semi-leptonic BDT selections. No preselection is applied in the analysis

Process	Events	Selected as	
	in $1.5\,\text {ab}^{-1}$	HAD	SL
${\mathrm{e}^{+}}{\mathrm{e}^{-}} \rightarrow \mathrm{t} {\bar{\mathrm{t}}} {\mathrm{H}}$, 6 jet, ${\mathrm{H}} \rightarrow {\mathrm{b}} {\bar{\mathrm{b}}}$	647	357	9
${\mathrm{e}^{+}}{\mathrm{e}^{-}} \rightarrow \mathrm{t} {\bar{\mathrm{t}}} {\mathrm{H}}$, 4 jet, ${\mathrm{H}} \rightarrow {\mathrm{b}} {\bar{\mathrm{b}}}$	623	62	233
${\mathrm{e}^{+}}{\mathrm{e}^{-}} \rightarrow \mathrm{t} {\bar{\mathrm{t}}} {\mathrm{H}}$, 2 jet, ${\mathrm{H}} \rightarrow {\mathrm{b}} {\bar{\mathrm{b}}}$	150	1	20
${\mathrm{e}^{+}}{\mathrm{e}^{-}} \rightarrow \mathrm{t} {\bar{\mathrm{t}}} {\mathrm{H}}$, 6 jet, ${\mathrm{H}} \not \rightarrow {\mathrm{b}} {\bar{\mathrm{b}}}$	473	38	8
${\mathrm{e}^{+}}{\mathrm{e}^{-}} \rightarrow \mathrm{t} {\bar{\mathrm{t}}} {\mathrm{H}}$, 4 jet, ${\mathrm{H}} \not \rightarrow {\mathrm{b}} {\bar{\mathrm{b}}}$	455	5	19
${\mathrm{e}^{+}}{\mathrm{e}^{-}} \rightarrow \mathrm{t} {\bar{\mathrm{t}}} {\mathrm{H}}$, 2 jet, ${\mathrm{H}} \not \rightarrow {\mathrm{b}} {\bar{\mathrm{b}}}$	110	0	1
${\mathrm{e}^{+}}{\mathrm{e}^{-}} \rightarrow \mathrm{t} {\bar{\mathrm{t}}} {\mathrm{b}} {\bar{\mathrm{b}}}$, 6 jet	824	287	8
${\mathrm{e}^{+}}{\mathrm{e}^{-}} \rightarrow \mathrm{t} {\bar{\mathrm{t}}} {\mathrm{b}} {\bar{\mathrm{b}}}$, 4 jet	794	44	175
${\mathrm{e}^{+}}{\mathrm{e}^{-}} \rightarrow \mathrm{t} {\bar{\mathrm{t}}} {\mathrm{b}} {\bar{\mathrm{b}}}$, 2 jet	191	1	14
${\mathrm{e}^{+}}{\mathrm{e}^{-}} \rightarrow \mathrm{t} {\bar{\mathrm{t}}} {\mathrm{Z}}$, 6 jet	2843	316	12
${\mathrm{e}^{+}}{\mathrm{e}^{-}} \rightarrow \mathrm{t} {\bar{\mathrm{t}}} {\mathrm{Z}}$, 4 jet	2738	49	170
${\mathrm{e}^{+}}{\mathrm{e}^{-}} \rightarrow \mathrm{t} {\bar{\mathrm{t}}} {\mathrm{Z}}$, 2 jet	659	1	13
${\mathrm{e}^{+}}{\mathrm{e}^{-}} \rightarrow \mathrm{t} {\bar{\mathrm{t}}}$	203,700	1399	523
${\mathrm{e}^{+}}{\mathrm{e}^{-}} \rightarrow \mathrm{q} \mathrm{q} \mathrm{q} \mathrm{q} \mathrm{l} {\nu } (\text {non-}\mathrm{t} {\bar{\mathrm{t}}})$	68,300	11	70
${\mathrm{e}^{+}}{\mathrm{e}^{-}} \rightarrow \mathrm{q} \mathrm{q} \mathrm{q} \mathrm{q}$	$2.0 \times 10^{6}$	195	0

To translate the measurement of the $\mathrm{t} {\bar{\mathrm{t}}} {\mathrm{H}}$ cross section into a measurement of the top Yukawa coupling, a correction is applied to take into account the contribution from the Higgsstrahlung diagram, where the Higgs boson is radiated off the intermediate ${\mathrm{Z}}$ boson in ${\mathrm{e}^{+}}{\mathrm{e}^{-}} \rightarrow \mathrm{t} {\bar{\mathrm{t}}}$ [62, 63]. To evaluate the small degradation in sensitivity, the $\textsc {Whizard}$ program is used to calculate the cross section for the inclusive process ${\mathrm{e}}^{+} {\mathrm{e}}{}{-} \rightarrow \mathrm{t} {\bar{\mathrm{t}}} {\mathrm{H}}$ as a function of the value of the top Yukawa coupling. The factor required to translate the measured cross section uncertainty into a coupling uncertainty is determined from the slope of the cross section at the SM value of the top Yukawa coupling, and is found to be:

$$\begin{aligned} \frac{\varDelta y_{\mathrm{t}}}{y_{\mathrm{t}}} = 0.53 \frac{\varDelta \sigma }{\sigma }\, , \end{aligned}$$

which is slightly larger than the factor of 0.50 expected without the Higgsstrahlung diagram. Thus, the expected precision on the top Yukawa coupling is:

$$\begin{aligned} \frac{\varDelta y_{\mathrm{t}}}{y_{\mathrm{t}}} = 4.2\% , \end{aligned}$$

for $1.5\,\text {ab}^{-1}$ of data at $\sqrt{s} =1.4\,\text {TeV}$ without beam polarisation. This value is expected to improve to about $4.0\%$ for the same amount of data collected using the $P^{({\mathrm{e}}{}{-})} = -80\%$ polarisation configuration [64]. Since the cross section for the $\mathrm{t} {\bar{\mathrm{t}}} {\mathrm{H}}$ cross section falls with increasing $\sqrt{s}$ (see Fig. 3), the precision with $2\,\text {ab}^{-1}$ at $3\,\text {TeV}$ is not expected to be better than the result presented here.

## Double Higgs production

**Fig. 24:**
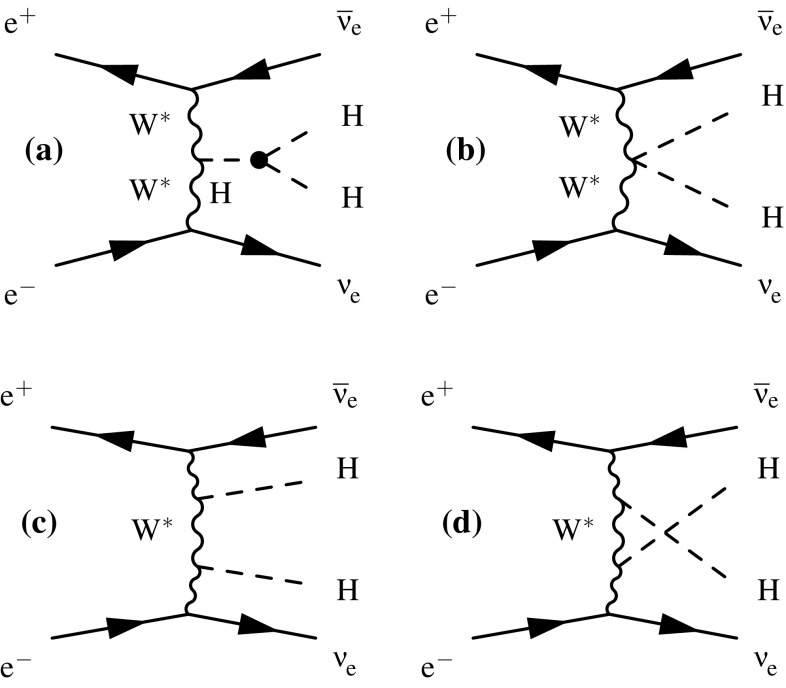
Feynman diagrams of leading-order processes that produce two Higgs bosons and missing energy at CLIC at $\sqrt{s} =1.4\,\text {TeV}$ and $3\,\text {TeV}$. The diagram (**a**) is sensitive to the trilinear Higgs self-coupling $\lambda$. The diagram (**b**) is sensitive to the quartic coupling $g_{{\mathrm{H}} {\mathrm{H}} {\mathrm{W}} {\mathrm{W}}}$. All four diagrams are included in the generated ${\mathrm{e}^{+}}{\mathrm{e}^{-}} \rightarrow {\mathrm{H}} {\mathrm{H}} {{\nu }}_{\!\mathrm{e}} {\bar{{\nu }}}_{\!\mathrm{e}}$ signal samples

In ${\mathrm{e}^{+}}{\mathrm{e}^{-}}$ collisions at high energy, double Higgs production, ${\mathrm{e}^{+}}{\mathrm{e}^{-}} \rightarrow {\mathrm{H}} {\mathrm{H}} {{\nu }}_{\!\mathrm{e}} {\bar{{\nu }}}_{\!\mathrm{e}}$, can occur through the processes shown in Fig. 24. Despite the small cross section (0.15 and 0.59 fb for CLIC operated at $\sqrt{s} =1.4$ and $3\,\text {TeV}$, respectively), measurements of the double Higgs production rate can be used to extract the Higgs boson trilinear self-coupling parameter $\lambda$, that determines the shape of the fundamental Higgs potential. BSM physics scenarios can introduce deviations of $\lambda$ from its SM value of up to tens of percent [65]. The physics potential for the measurement of this coupling has been studied using the CLIC_ILD detector model for $1.5\,\text {ab}^{-1}$ of data at $\sqrt{s} =1.4\,\text {TeV}$ and for $2\,\text {ab}^{-1}$ of data at $\sqrt{s} =3\,\text {TeV}$. The process ${\mathrm{e}^{+}}{\mathrm{e}^{-}} \rightarrow {\mathrm{H}} {\mathrm{H}} {\mathrm{e}^{+}}{\mathrm{e}^{-}}$ has not been included as its cross section is about an order of magnitude smaller compared to ${\mathrm{e}^{+}}{\mathrm{e}^{-}} \rightarrow {\mathrm{H}} {\mathrm{H}} {{\nu }}_{\!\mathrm{e}} {\bar{{\nu }}}_{\!\mathrm{e}}$.

Two signatures for ${\mathrm{e}^{+}}{\mathrm{e}^{-}} \rightarrow {\mathrm{H}} {\mathrm{H}} {{\nu }}_{\!\mathrm{e}} {\bar{{\nu }}}_{\!\mathrm{e}}$ production are considered in the following: ${\mathrm{H}} {\mathrm{H}} \rightarrow {\mathrm{b}} {\bar{\mathrm{b}}} {\mathrm{b}} {\bar{\mathrm{b}}}$ and ${\mathrm{H}} {\mathrm{H}} \rightarrow {\mathrm{b}} {\bar{\mathrm{b}}} {\mathrm{W}} {\mathrm{W}} ^*\rightarrow {\mathrm{b}} {\bar{\mathrm{b}}} \mathrm{q}{\bar{\mathrm{q}}} \mathrm{q}{\bar{\mathrm{q}}}$. All events without isolated leptons are considered for the analysis. These events are clustered into four jets using the $k_{\mathrm {t}}$ algorithm. Flavour tagging information is obtained from the LcfiPlus package. Events where the sum of the b-tag values of the four jets is smaller than 2.3 and the hadronic system fulfills the requirement $-\log _{10}(y_{34}) < 3.7 (3.6)$ at 1.4 $\text {TeV}$ (3 $\text {TeV}$) are considered as ${\mathrm{b}} {\bar{\mathrm{b}}} {\mathrm{W}} {\mathrm{W}} ^*$ candidates, while all other events are considered as ${\mathrm{b}} {\bar{\mathrm{b}}} {\mathrm{b}} {\bar{\mathrm{b}}}$ candidates. The following steps of the analysis are performed separately for the two final states.

**Table 28:** Preselection and selection efficiencies for the double Higgs signal and most important background processes in both considered decay channels at $\sqrt{s} =1.4\,\text {TeV}$. The numbers of events correspond to $1.5\,\text {ab}^{-1}$. Contributions from all other backgrounds are found to be negligibly small

Process	$\sigma /\text {fb}$	$\varepsilon _\text {presel}$ (%)	$\varepsilon _{\text {BDT}}$ (%)	$N_{\text {BDT}}$
${\mathrm{H}} {\mathrm{H}} {{\nu }}_{\!\mathrm{e}} {\bar{{\nu }}}_{\!\mathrm{e}} ;\,{\mathrm{H}} {\mathrm{H}} \rightarrow {\mathrm{b}} {\bar{\mathrm{b}}} {\mathrm{b}} {\bar{\mathrm{b}}}$	0.047	94	24	16
${\mathrm{H}} {\mathrm{H}} {{\nu }}_{\!\mathrm{e}} {\bar{{\nu }}}_{\!\mathrm{e}} ;\,{\mathrm{H}} {\mathrm{H}} \rightarrow other$	0.102	29	0.77	0.3
${\mathrm{e}^{+}}{\mathrm{e}^{-}} \rightarrow \mathrm{q}{\bar{\mathrm{q}}} \mathrm{q}{\bar{\mathrm{q}}} \nu \bar{\nu }$	23	6.2	0.38	8
${\mathrm{e}^{+}}{\mathrm{e}^{-}} \rightarrow \mathrm{q}{\bar{\mathrm{q}}} \mathrm{q}{\bar{\mathrm{q}}} \mathrm{l} {\nu }$	110	16	0.03	7
${\mathrm{e}^{+}}{\mathrm{e}^{-}} \rightarrow \mathrm{q}{\bar{\mathrm{q}}} {\mathrm{H}} \nu \bar{\nu }$	1.5	39	2.0	18
$\mathrm{e} ^{\pm } {\upgamma } \rightarrow {\nu } \mathrm{q}{\bar{\mathrm{q}}} \mathrm{q}{\bar{\mathrm{q}}}$	154	13	0.01	3
$\mathrm{e} ^{\pm } {\upgamma } \rightarrow \mathrm{q} \mathrm{q} {\mathrm{H}} \nu$	30	28	0.01	1
${\mathrm{H}} {\mathrm{H}} {{\nu }}_{\!\mathrm{e}} {\bar{{\nu }}}_{\!\mathrm{e}} ;\,{\mathrm{H}} {\mathrm{H}} \rightarrow {\mathrm{b}} {\bar{\mathrm{b}}} {\mathrm{W}} {\mathrm{W}} ^*$;	0.018	60	8.2	1.3
$${{\mathrm{W}}}^{+} {{\mathrm{W}}}^{-} \rightarrow \mathrm{q} {\bar{\mathrm{q}}} \mathrm{q} {\bar{\mathrm{q}}} $$				
${\mathrm{H}} {\mathrm{H}} {{\nu }}_{\!\mathrm{e}} {\bar{{\nu }}}_{\!\mathrm{e}} ;\,{\mathrm{H}} {\mathrm{H}} \rightarrow {\mathrm{b}} {\bar{\mathrm{b}}} {\mathrm{b}} {\bar{\mathrm{b}}}$	0.047	15	0.5	0.1
${\mathrm{H}} {\mathrm{H}} {{\nu }}_{\!\mathrm{e}} {\bar{{\nu }}}_{\!\mathrm{e}} ;\,{\mathrm{H}} {\mathrm{H}} \rightarrow other$	0.085	20	1.7	0.5
${\mathrm{e}^{+}}{\mathrm{e}^{-}} \rightarrow \mathrm{q}{\bar{\mathrm{q}}} \mathrm{q}{\bar{\mathrm{q}}} \nu \bar{\nu }$	23	17	0.002	0.1
${\mathrm{e}^{+}}{\mathrm{e}^{-}} \rightarrow \mathrm{q}{\bar{\mathrm{q}}} \mathrm{q}{\bar{\mathrm{q}}} \mathrm{l} {\nu }$	110	10	0.01	2
${\mathrm{e}^{+}}{\mathrm{e}^{-}} \rightarrow \mathrm{q}{\bar{\mathrm{q}}} {\mathrm{H}} \nu \bar{\nu }$	1.5	35	0.1	0.8
$\mathrm{e} ^{\pm } {\upgamma } \rightarrow {\nu } \mathrm{q}{\bar{\mathrm{q}}} \mathrm{q}{\bar{\mathrm{q}}}$	154	22	0.0045	2
$\mathrm{e} ^{\pm } {\upgamma } \rightarrow \mathrm{q} \mathrm{q} {\mathrm{H}} \nu$	30	27	0.02	3

At 1.4 $\text {TeV}$, a cut on the sum of the four b-tag values of at least 1.5 is imposed for ${\mathrm{b}} {\bar{\mathrm{b}}} {\mathrm{b}} {\bar{\mathrm{b}}}$ candidate events. Those events with a sum of the four b-tag values less than 2.3 are required to have a sum of the jet energies of at least 150 $\text {GeV}$ and a second highest jet transverse momentum of at least 25 $\text {GeV}$. A cut on the sum of the four b-tag values of at least 2.3 is imposed for all events at 3 TeV. The jets are grouped into two Higgs boson candidates by minimising $|m_{ij}-m_{kl}|$, where $m_{ij}$ and $m_{kl}$ are the invariant masses of the jet pairs used to reconstruct the Higgs candidates. For events passing the preselection cuts, at both energies BDT classifiers with the same 10 input variables are used to suppress the backgrounds further.

For the ${\mathrm{b}} {\bar{\mathrm{b}}} {\mathrm{W}} {\mathrm{W}} ^*$ final state, the events are re-clustered into six jets. These jets are then grouped into ${\mathrm{W}}$ and ${\mathrm{H}}$ candidates by minimising:

$$\begin{aligned} \chi ^2=\frac{(m_{ij}-m_{{\mathrm{H}}})^{2}}{\sigma _{{\mathrm{H}} \rightarrow {\mathrm{b}} {\bar{\mathrm{b}}} }^2}+\frac{(m_{klmn}-m_{{\mathrm{H}}})^{2}}{\sigma _{{\mathrm{H}} \rightarrow {\mathrm{W}} {\mathrm{W}} ^*}^2}+\frac{(m_{kl}-m_{{\mathrm{W}}})^{2}}{\sigma _{{\mathrm{W}}}^2}, \end{aligned}$$

where $m_{ij}$ and $m_{klmn}$ are the jet combinations used to reconstruct the Higgs candidates, $m_{kl}$ is the invariant mass of the jet pair used to reconstruct the W candidate and $\sigma _{{\mathrm{H}} \rightarrow {\mathrm{b}} {\bar{\mathrm{b}}} }$, $\sigma _{{\mathrm{H}} \rightarrow {\mathrm{W}} {\mathrm{W}} ^*}$, $\sigma _{{\mathrm{W}}}$ are the estimated invariant mass resolutions for the reconstruction of ${\mathrm{H}} \rightarrow {\mathrm{b}} {\bar{\mathrm{b}}}$, ${\mathrm{H}} \rightarrow {\mathrm{W}} {\mathrm{W}} ^*$ and ${\mathrm{W}}$ decays. Events with an invariant mass of the two H boson candidates above 150 $\text {GeV}$ are considered further. At 3 $\text {TeV}$ a highest b-tag value of at least 0.7 is required while at 1.4 $\text {TeV}$ the second highest b-tag values has to be larger than 0.2 and the visible transverse momentum has to be larger than 30 GeV. After this preselection, BDT classifiers using 32 input variables are used to suppress the backgrounds further.

The event selections for both studies at 1.4 and 3 $\text {TeV}$ are summarised in Tables 28 and 29, respectively. Combining the expected precisions on the cross sections for both signatures leads to:

$$\begin{aligned} \frac{\varDelta [\sigma ({\mathrm{H}} {\mathrm{H}} {{\nu }}_{\!\mathrm{e}} {\bar{{\nu }}}_{\!\mathrm{e}} )]}{\sigma ({\mathrm{H}} {\mathrm{H}} {{\nu }}_{\!\mathrm{e}} {\bar{{\nu }}}_{\!\mathrm{e}} )}&= 44\% \ \text {at} \ 1.4\,\text {TeV}, \\ \frac{\varDelta [\sigma ({\mathrm{H}} {\mathrm{H}} {{\nu }}_{\!\mathrm{e}} {\bar{{\nu }}}_{\!\mathrm{e}} )]}{\sigma ({\mathrm{H}} {\mathrm{H}} {{\nu }}_{\!\mathrm{e}} {\bar{{\nu }}}_{\!\mathrm{e}} )}&= 20\% \ \text {at} \ 3\,\text {TeV}. \end{aligned}$$

**Table 29:** Preselection and selection efficiencies for the double Higgs signal and most important background processes in both considered decay channels at $\sqrt{s} =3\,\text {TeV}$. The numbers of events correspond to $2\,\text {ab}^{-1}$. Contributions from all other backgrounds are found to be negligibly small

Process	$\sigma /\text {fb}$	$\varepsilon _\text {presel}$ (%)	$\varepsilon _{\text {BDT}}$ (%)	$N_{\text {BDT}}$
${\mathrm{H}} {\mathrm{H}} {{\nu }}_{\!\mathrm{e}} {\bar{{\nu }}}_{\!\mathrm{e}} ;\,{\mathrm{H}} {\mathrm{H}} \rightarrow {\mathrm{b}} {\bar{\mathrm{b}}} {\mathrm{b}} {\bar{\mathrm{b}}}$	0.19	66	24	61
${\mathrm{H}} {\mathrm{H}} {{\nu }}_{\!\mathrm{e}} {\bar{{\nu }}}_{\!\mathrm{e}} ;\,{\mathrm{H}} {\mathrm{H}} \rightarrow other$	0.40	5.4	3.2	1
${\mathrm{e}^{+}}{\mathrm{e}^{-}} \rightarrow \mathrm{q}{\bar{\mathrm{q}}} \mathrm{q}{\bar{\mathrm{q}}}$	547	0.16	0.16	3
${\mathrm{e}^{+}}{\mathrm{e}^{-}} \rightarrow \mathrm{q}{\bar{\mathrm{q}}} \mathrm{q}{\bar{\mathrm{q}}} \nu \bar{\nu }$	72	1.8	0.68	17
${\mathrm{e}^{+}}{\mathrm{e}^{-}} \rightarrow \mathrm{q}{\bar{\mathrm{q}}} \mathrm{q}{\bar{\mathrm{q}}} \mathrm{l} {\nu }$	107	1.8	0.15	6
${\mathrm{e}^{+}}{\mathrm{e}^{-}} \rightarrow \mathrm{q}{\bar{\mathrm{q}}} {\mathrm{H}} \nu \bar{\nu }$	4.7	18	3.0	50
$\mathrm{e} ^{\pm } {\upgamma } \rightarrow {\nu } \mathrm{q}{\bar{\mathrm{q}}} \mathrm{q}{\bar{\mathrm{q}}}$	523	1.2	0.09	11
$\mathrm{e} ^{\pm } {\upgamma } \rightarrow \mathrm{q} \mathrm{q} {\mathrm{H}} \nu$	116	2.7	0.14	9
${\mathrm{H}} {\mathrm{H}} {{\nu }}_{\!\mathrm{e}} {\bar{{\nu }}}_{\!\mathrm{e}} ;\,{\mathrm{H}} {\mathrm{H}} \rightarrow {\mathrm{b}} {\bar{\mathrm{b}}} {\mathrm{W}} {\mathrm{W}} ^*$;	0.07	62	12	10
$${{\mathrm{W}}}^{+} {{\mathrm{W}}}^{-} \rightarrow \mathrm{q} {\bar{\mathrm{q}}} \mathrm{q} {\bar{\mathrm{q}}} $$				
${\mathrm{H}} {\mathrm{H}} {{\nu }}_{\!\mathrm{e}} {\bar{{\nu }}}_{\!\mathrm{e}} ;\,{\mathrm{H}} {\mathrm{H}} \rightarrow {\mathrm{b}} {\bar{\mathrm{b}}} {\mathrm{b}} {\bar{\mathrm{b}}}$	0.19	19	1.5	1
${\mathrm{H}} {\mathrm{H}} {{\nu }}_{\!\mathrm{e}} {\bar{{\nu }}}_{\!\mathrm{e}} ;\,{\mathrm{H}} {\mathrm{H}} \rightarrow other$	0.34	20	3.6	5
${\mathrm{e}^{+}}{\mathrm{e}^{-}} \rightarrow \mathrm{q}{\bar{\mathrm{q}}} \mathrm{q}{\bar{\mathrm{q}}}$	547	1.4	0.01	1
${\mathrm{e}^{+}}{\mathrm{e}^{-}} \rightarrow \mathrm{q}{\bar{\mathrm{q}}} \mathrm{q}{\bar{\mathrm{q}}} \nu \bar{\nu }$	72	9.0	0.05	6
${\mathrm{e}^{+}}{\mathrm{e}^{-}} \rightarrow \mathrm{q}{\bar{\mathrm{q}}} \mathrm{q}{\bar{\mathrm{q}}} \mathrm{l} {\nu }$	107	7.3	0.05	8
${\mathrm{e}^{+}}{\mathrm{e}^{-}} \rightarrow \mathrm{q}{\bar{\mathrm{q}}} {\mathrm{H}} \nu \bar{\nu }$	4.8	32	0.6	19
$\mathrm{e} ^{\pm } {\upgamma } \rightarrow {\nu } \mathrm{q}{\bar{\mathrm{q}}} \mathrm{q}{\bar{\mathrm{q}}}$	523	15	0.04	67
$\mathrm{e} ^{\pm } {\upgamma } \rightarrow \mathrm{q} \mathrm{q} {\mathrm{H}} \nu$	116	27	0.2	140

The double Higgs production cross section is sensitive to the trilinear Higgs self-coupling $\lambda$. Since diagrams not involving $\lambda$ also contribute to the ${\mathrm{e}^{+}}{\mathrm{e}^{-}} \rightarrow {\mathrm{H}} {\mathrm{H}} {{\nu }}_{\!\mathrm{e}} {\bar{{\nu }}}_{\!\mathrm{e}}$ process, their effect must be taken into account. The relation between the relative uncertainty on the cross section and the relative uncertainty of the Higgs trilinear coupling can be approximated as:

$$\begin{aligned} \frac{\varDelta \lambda }{\lambda } \approx \kappa \cdot \frac{\varDelta [\sigma ({\mathrm{H}} {\mathrm{H}} {{\nu }}_{\!\mathrm{e}} {\bar{{\nu }}}_{\!\mathrm{e}} )]}{\sigma ({\mathrm{H}} {\mathrm{H}} {{\nu }}_{\!\mathrm{e}} {\bar{{\nu }}}_{\!\mathrm{e}} )}. \end{aligned}$$

**Fig. 25:**
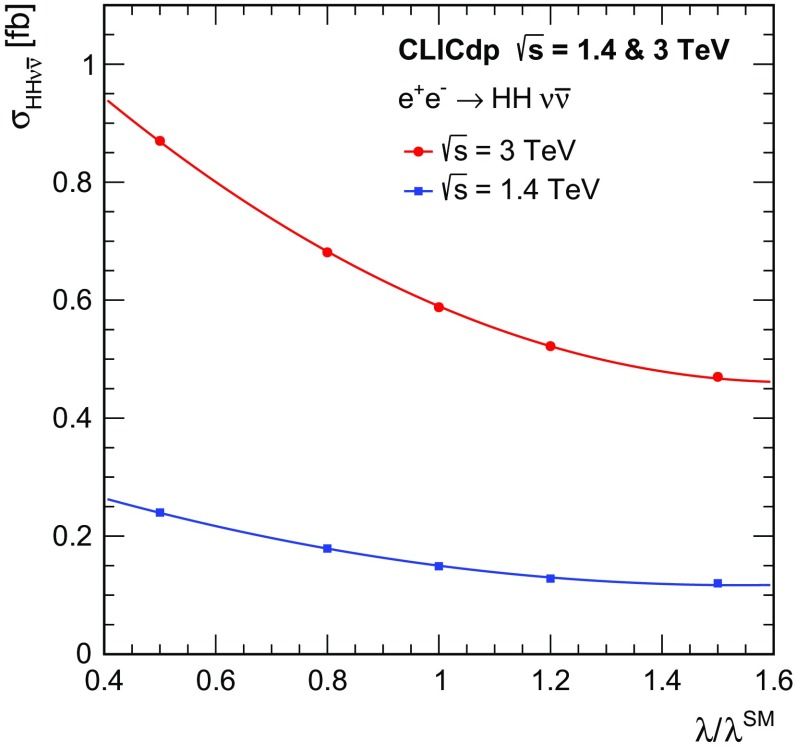
Cross section for the ${\mathrm{e}^{+}}{\mathrm{e}^{-}} \rightarrow {\mathrm{H}} {\mathrm{H}} {{\nu }}_{\!\mathrm{e}} {\bar{{\nu }}}_{\!\mathrm{e}}$ process as a function of the ratio $\lambda /\lambda ^\text {SM}$ at $\sqrt{s} =1.4$ and $3\,\text {TeV}$

The value of $\kappa$ can be determined from the $\textsc {Whizard}$ generator by parameterising the ${\mathrm{e}^{+}}{\mathrm{e}^{-}} \rightarrow {\mathrm{H}} {\mathrm{H}} {{\nu }}_{\!\mathrm{e}} {\bar{{\nu }}}_{\!\mathrm{e}}$ cross section as a function of the input value for $\lambda$, as indicated in Fig. 25. The fact that the slope is negative indicates that the main dependence on $\lambda$ enters through interference with other SM diagrams. The value of $\kappa$ is determined from the derivative of the cross section dependence as a function of $\lambda$, evaluated at its SM value, giving $\kappa = 1.22$ and $\kappa = 1.47$ at 1.4 TeV and 3 TeV, respectively. However, this method does not account for the possibility that the event selection might preferentially favour some diagrams over others, and hence change the analysis sensitivity to $\lambda$.

In the case of zero beam polarisation, the combined cross sections for double Higgs production give:

$$\begin{aligned} \varDelta \lambda /\lambda&= 54\% \ \ \text {at} \ \sqrt{s} =1.4\,\text {TeV},\\ \varDelta \lambda /\lambda&= 29\% \ \ \text {at} \ \sqrt{s} =3\,\text {TeV}. \end{aligned}$$

Because the process involving the trilinear Higgs coupling involves *t*-channel ${\mathrm{W}} {\mathrm{W}}$-fusion, it can be enhanced by operating with polarised beams. For the case of $P{(\mathrm{e}^{-})} = -80\%$, this yields:

$$\begin{aligned} \varDelta \lambda /\lambda&= 40\% \ \ \text {at} \ \sqrt{s} =1.4\,\text {TeV},\\ \varDelta \lambda /\lambda&= 22\% \ \ \text {at} \ \sqrt{s} =3\,\text {TeV}. \end{aligned}$$

The statistical precision on $\lambda$ improves to $26\%$ for unpolarised beams and to $19\%$ for $P({\mathrm{e}}{}{-}) = -80\%$ when combining both energy stages. These results will be improved further using template fits to the BDT output distributions as the different diagrams contributing to double Higgs production lead to different event topologies.

## Higgs mass

At a centre-of-mass energy of $\sqrt{s} =350\,\text {GeV}$, the Higgs boson mass can be measured in the ${\mathrm{e}^{+}}{\mathrm{e}^{-}} \rightarrow {\mathrm{Z}} {\mathrm{H}}$ process. The Higgs boson mass can be extracted from the four-momentum recoiling against in Z boson using ${\mathrm{Z}} \rightarrow {\mathrm{e}^{+}}{\mathrm{e}^{-}}$ or ${\mathrm{Z}} \rightarrow {{{\upmu }}^{+} {{\upmu }}^{-}}$ events as described in Sect. 5. Due to the small branching ratios for leptonic Z boson decay channels and the impact of the CLIC beamstrahlung spectrum, the achievable precision is limited to $110\,\text {MeV}$.

In a different approach, the Higgs mass is reconstructed from the measured four-vectors of its decay products. The best precision is expected using ${\mathrm{H}} \rightarrow {\mathrm{b}} {\bar{\mathrm{b}}}$ decays in ${\mathrm{e}^{+}}{\mathrm{e}^{-}} \rightarrow {\mathrm{H}} {{\nu }}_{\!\mathrm{e}} {\bar{{\nu }}}_{\!\mathrm{e}}$ events at high energy. For this purpose, the analysis described in Sect. 6.1 has been modified. After the preselection, a single BDT is used at each energy to select ${\mathrm{H}} \rightarrow {\mathrm{b}} {\bar{\mathrm{b}}}$ decays. In contrast to the coupling measurement, the flavour tagging information is included in the BDT classifier.

**Fig. 26:**
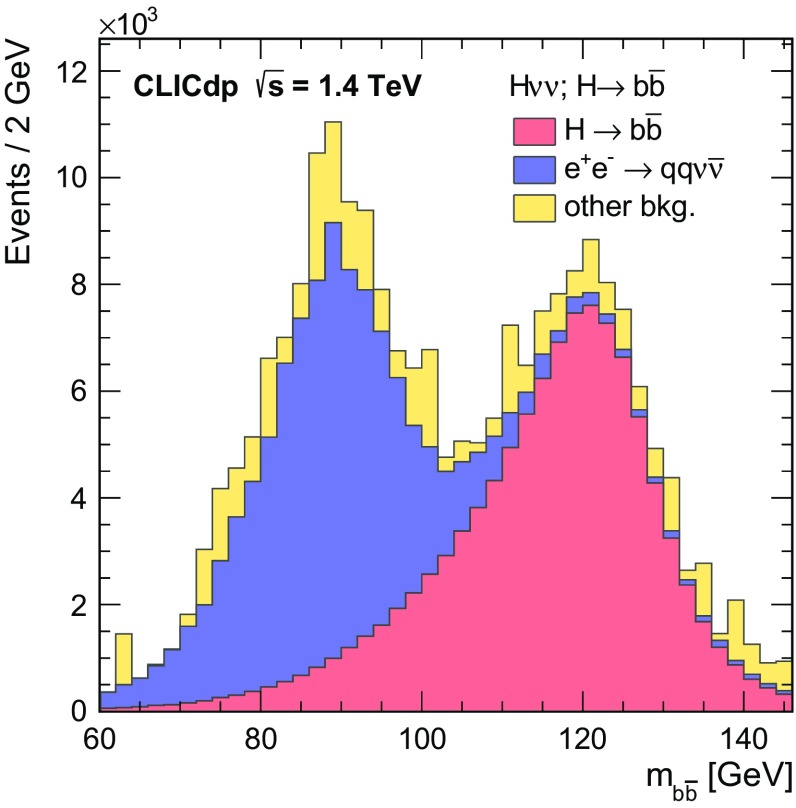
Reconstructed di-jet invariant mass distribution of selected ${\mathrm{H}} \rightarrow {\mathrm{b}} {\bar{\mathrm{b}}}$ events at $\sqrt{s} =1.4\,\text {TeV}$, showing the signal and backgrounds as stacked histograms. The distributions are normalised to an integrated luminosity of $1.5\,\text {ab}^{-1}$

The invariant mass distribution for selected events at $\sqrt{s} =1.4\,\text {TeV}$ is shown in Fig. 26. The Higgs mass is extracted in the range $105\,\text {GeV}< m_{{\mathrm{b}} {\bar{\mathrm{b}}}} < 145\,\text {GeV}$ where good purity of the signal channel is achieved. At the nominal Z boson mass, a second peak from ${\mathrm{e}^{+}}{\mathrm{e}^{-}} \rightarrow {\mathrm{Z}} {{\nu }}_{\!\mathrm{e}} {\bar{{\nu }}}_{\!\mathrm{e}}; {\mathrm{Z}} \rightarrow {\mathrm{b}} {\bar{\mathrm{b}}}$ events is visible. These events can be used to calibrate the jet energy scale for the precision measurement of the Higgs boson mass.

A template fit using ${\mathrm{e}^{+}}{\mathrm{e}^{-}} \rightarrow {\mathrm{H}} {{\nu }}_{\!\mathrm{e}} {\bar{{\nu }}}_{\!\mathrm{e}}; {\mathrm{H}} \rightarrow {\mathrm{b}} {\bar{\mathrm{b}}}$ event samples generated using slightly shifted values for the Higgs mass parameter is performed. The Higgs mass and production cross section are extracted simultaneously. The following statistical precisions on the Higgs mass are achieved:

$$\begin{aligned} \varDelta (m_{{\mathrm{H}}})&= 47\,\text {MeV}\ \text {at} \ 1.4\,\text {TeV}, \\ \varDelta (m_{{\mathrm{H}}})&= 44\,\text {MeV}\ \text {at} \ 3\,\text {TeV}. \end{aligned}$$

A combination of both energy stages would lead to a precision of $32\,\text {MeV}$.

## Systematic uncertainties

The complete Higgs physics potential of a CLIC collider implemented in three energy stages is described in this paper. The expected statistical uncertainties given in the previous sections do not include potential sources of systematic uncertainty. The obtained results therefore illustrate the level of precision desirable for the control of systematic effects. This is crucial input for the choice of detector technologies and the development of calibration procedures in the coming years.

A comprehensive study of systematic uncertainties requires more knowledge on the technical implementation of the detector. This is beyond the scope of this paper. At this stage, the impact of potentially relevant sources of systematic uncertainty is discussed. The measurements of $\sigma ({\mathrm{H}} {{\nu }}_{\!\mathrm{e}} {\bar{{\nu }}}_{\!\mathrm{e}})\times BR ({\mathrm{H}} \rightarrow {\mathrm{b}} {\bar{\mathrm{b}}})$ and the Higgs mass at $\sqrt{s} =3$ $\text {TeV}$, described in Sects. 6.1 and 10, are used as examples. These measurements are the most challenging test cases for many systematic effects due to the very small expected statistical uncertainties of 0.3% and 44 $\text {MeV}$, respectively. In addition, the experimental conditions are most challenging at 3 $\text {TeV}$.

The impact of theoretical uncertainties on the Higgs branching fractions is discussed in Sect. 12 in the context of a combined fit.

- **Luminosity spectrum:** A good knowledge of the luminosity spectrum is mandatory for precision Higgs physics at CLIC. The reconstruction of the CLIC luminosity spectrum from Bhabha scattering events is described in [66]. A model of the CLIC luminosity spectrum with 19 free parameters is assumed. The expected uncertainties of these parameters and their correlations are propagated to the measurement of $\sigma ({\mathrm{H}} {{\nu }}_{\!\mathrm{e}} {\bar{{\nu }}}_{\!\mathrm{e}})\times BR ({\mathrm{H}} \rightarrow {\mathrm{b}} {\bar{\mathrm{b}}})$ and lead to a systematic uncertainty of 0.15%. The luminosity spectrum affects the event rate more than the observed invariant mass of the two jets. Concerning the Higgs mass extraction, the luminosity spectrum is not expected to represent a dominant source of systematic uncertainty since the cross section is a free parameter in the template fit.
- **Total luminosity:** The expected statistical precision of the $\sigma ({\mathrm{H}} {{\nu }}_{\!\mathrm{e}} {\bar{{\nu }}}_{\!\mathrm{e}})\times BR ({\mathrm{H}} \rightarrow {\mathrm{b}} {\bar{\mathrm{b}}})$ measurement indicates the desired precision for the knowledge of the total luminosity. It is expected that an accuracy of a few permille can be achieved using the luminometer envisaged for CLIC [67, 68].
- **Beam polarisation:** The knowledge of the beam polarisation at the interaction point is most important for the measurement of ${\mathrm{W}} {\mathrm{W}}$-fusion events at high energy. The beam polarisation can be controlled to a level of 0.2% using single ${\mathrm{W}}$, ${\mathrm{Z}}$ and ${\upgamma }$ events with missing energy [69]. The resulting systematic uncertainty on $\sigma ({\mathrm{H}} {{\nu }}_{\!\mathrm{e}} {\bar{{\nu }}}_{\!\mathrm{e}})\times BR ({\mathrm{H}} \rightarrow {\mathrm{b}} {\bar{\mathrm{b}}})$ is 0.1%. For the Higgs mass measurement, the effect of the estimated beam polarisation uncertainty is negligible.
- **Jet energy scale:** The measurement of the Higgs boson mass using ${\mathrm{H}} \rightarrow {\mathrm{b}} {\bar{\mathrm{b}}}$ decays requires a precise knowledge of the energy scale correction for b-jets. An uncertainty on the jet energy scale of $3.5\times 10^{-4}$ leads to a systematic uncertainty on the Higgs mass similar to the statistical error at 3 TeV. The same jet energy scale uncertainty would have negligible impact on $\sigma ({\mathrm{H}} {{\nu }}_{\!\mathrm{e}} {\bar{{\nu }}}_{\!\mathrm{e}})\times BR ({\mathrm{H}} \rightarrow {\mathrm{b}} {\bar{\mathrm{b}}})$. A suitable process for the calibration is ${\mathrm{e}^{+}}{\mathrm{e}^{-}} \rightarrow {\mathrm{Z}} {{\nu }}_{\!\mathrm{e}} {\bar{{\nu }}}_{\!\mathrm{e}}; {\mathrm{Z}} \rightarrow {\mathrm{b}} {\bar{\mathrm{b}}}$ which is kinematically similar to Higgs production in ${\mathrm{W}} {\mathrm{W}}$-fusion. $\sigma ({\mathrm{Z}} {{\nu }}_{\!\mathrm{e}} {\bar{{\nu }}}_{\!\mathrm{e}})\times BR ({\mathrm{Z}} \rightarrow {\mathrm{b}} {\bar{\mathrm{b}}}) = 276$ fb leads to an expected number of events for calibration which is slightly larger than the signal event sample. To improve the precision further, additional high-statistics ${\mathrm{Z}}$ boson samples would be needed. Generator-level studies show that $\mathrm{e} ^{\pm } {\upgamma } \rightarrow {\mathrm{Z}} \mathrm{e} ^{\pm }; {\mathrm{Z}} \rightarrow {\mathrm{b}} {\bar{\mathrm{b}}}$ with a cross section about one order of magnitude larger compared to the signal process is a promising channel for this purpose.
- **Flavour tagging:** Several of the precision measurements discussed in this paper rely on b-tagging information. The calibration of the flavour tagging at CLIC is a topic for future study. To illustrate the impact of a non-perfect understanding of the mistag rate for charm and light quark jets, an ad hoc variation of the b-tag distributions for jets in background events is performed. Even after the BDT selection, the background contains only very few b-jets in the $\sigma ({\mathrm{H}} {{\nu }}_{\!\mathrm{e}} {\bar{{\nu }}}_{\!\mathrm{e}})\times BR ({\mathrm{H}} \rightarrow {\mathrm{b}} {\bar{\mathrm{b}}})$ analysis. First, the b-tag distributions for both jets were decreased (increased) by 0.5% using event reweighting for values below (above) the median keeping the overall number of background events constant. The opposite variation is applied in a second step. These variations lead to a $\pm 0.25\%$ change of the result. As the flavour tagging efficiency mostly affects the event rate, it is not expected to be a dominant source of systematic uncertainty for the Higgs mass measurement.

In summary, it seems possible to control the systematic uncertainties discussed above with similar or better precision compared to the statistical uncertainty for the measurement of $\sigma ({\mathrm{H}} {{\nu }}_{\!\mathrm{e}} {\bar{{\nu }}}_{\!\mathrm{e}})\times BR ({\mathrm{H}} \rightarrow {\mathrm{b}} {\bar{\mathrm{b}}})$. An excellent understanding of the b-jet energy scale is necessary for a competitive Higgs mass measurement at CLIC.

Many of the analyses described in this paper, especially where harmonization is relevant, will require a careful tuning of the Monte Carlo models using other high-precision processes. Such an investigation is beyond the scope of this first study of Higgs physics at CLIC presented here.

## Combined fits

The results discussed in the preceding sections are summarised in Tables 30 and 31. From the $\sigma$ and $\sigma \times BR$ measurements given in the tables the Higgs coupling parameters and total width are extracted by a global fit as described below. Here, a $-80\%$ electron polarisation is assumed for the 1.4 and the $3\,\text {TeV}$ stages. The increase in cross section is taken into account by multiplying the event rates with a factor of 1.8 for all WW-fusion measurements (see Table 3), resulting in a reduction of the uncertainties by a factor of $\sqrt{1.8}$. This approach is conservative since it assumes that all backgrounds including those from *s*-channel processes, which do not receive the same enhancement by polarisation, scale with the same factor.

**Table 30:** Summary of the precisions obtainable for the Higgs observables in the first stage of CLIC for an integrated luminosity of $500\,\text {fb}^{-1}$ at $\sqrt{s} =350\,\text {GeV}$, assuming unpolarised beams. For the branching ratios, the measurement precision refers to the expected statistical uncertainty on the product of the relevant cross section and branching ratio; this is equivalent to the expected statistical uncertainty of the product of couplings divided by $\varGamma _{{\mathrm{H}}}$ as indicated in the third column

Channel	Measurement	Observable	Statistical precision
			$$350\,\text {GeV} $$
			$$500\,\text {fb}^{-1} $$
${\mathrm{Z}} {\mathrm{H}}$	Recoil mass distribution	$m_{{\mathrm{H}}}$	$110\,\text {MeV}$
${\mathrm{Z}} {\mathrm{H}}$	$\sigma ({\mathrm{Z}} {\mathrm{H}})\times BR ({\mathrm{H}} \rightarrow \text {invisible})$	$\varGamma _\text {inv}$	$0.6\%$
${\mathrm{Z}} {\mathrm{H}}$	$\sigma ({\mathrm{Z}} {\mathrm{H}})\times BR ({\mathrm{Z}} \rightarrow {\mathrm{l}}^{+} {\mathrm{l}}^{-})$	$g_{{\mathrm{H}} {\mathrm{Z}} {\mathrm{Z}}} ^{2}$	$3.8\%$
${\mathrm{Z}} {\mathrm{H}}$	$\sigma ({\mathrm{Z}} {\mathrm{H}})\times BR ({\mathrm{Z}} \rightarrow \mathrm{q} {\bar{\mathrm{q}}})$	$g_{{\mathrm{H}} {\mathrm{Z}} {\mathrm{Z}}} ^{2}$	$1.8\%$
${\mathrm{Z}} {\mathrm{H}}$	$\sigma ({\mathrm{Z}} {\mathrm{H}})\times BR ({\mathrm{H}} \rightarrow {\mathrm{b}} {\bar{\mathrm{b}}})$	$g_{{\mathrm{H}} {\mathrm{Z}} {\mathrm{Z}}} ^{2}g_{{\mathrm{H}} {\mathrm{b}} {\mathrm{b}}} ^{2}/\varGamma _{{\mathrm{H}}}$	$0.86\%$
${\mathrm{Z}} {\mathrm{H}}$	$\sigma ({\mathrm{Z}} {\mathrm{H}})\times BR ({\mathrm{H}} \rightarrow {\mathrm{c}} {\bar{{\mathrm{c}}}})$	$g_{{\mathrm{H}} {\mathrm{Z}} {\mathrm{Z}}} ^{2}g_{{\mathrm{H}} {\mathrm{c}} {\mathrm{c}}} ^2/\varGamma _{{\mathrm{H}}}$	$14\%$
${\mathrm{Z}} {\mathrm{H}}$	$\sigma ({\mathrm{Z}} {\mathrm{H}})\times BR ({\mathrm{H}} \rightarrow {\mathrm{g}} {\mathrm{g}})$		$6.1\%$
${\mathrm{Z}} {\mathrm{H}}$	$\sigma ({\mathrm{Z}} {\mathrm{H}})\times BR ({\mathrm{H}} \rightarrow \uptau ^{+} \uptau ^{-} )$	$g_{{\mathrm{H}} {\mathrm{Z}} {\mathrm{Z}}} ^{2}g_{{\mathrm{H}} \uptau \uptau } ^{2}/\varGamma _{{\mathrm{H}}}$	$6.2\%$
${\mathrm{Z}} {\mathrm{H}}$	$\sigma ({\mathrm{Z}} {\mathrm{H}})\times BR ({\mathrm{H}} \rightarrow {\mathrm{W}} {\mathrm{W}} ^*)$	$g_{{\mathrm{H}} {\mathrm{Z}} {\mathrm{Z}}} ^{2}g_{{\mathrm{H}} {\mathrm{W}} {\mathrm{W}}} ^{2}/\varGamma _{{\mathrm{H}}}$	$5.1\%$
${\mathrm{H}} {{\nu }}_{\!\mathrm{e}} {\bar{{\nu }}}_{\!\mathrm{e}}$	$\sigma ({\mathrm{H}} {{\nu }}_{\!\mathrm{e}} {\bar{{\nu }}}_{\!\mathrm{e}})\times BR ({\mathrm{H}} \rightarrow {\mathrm{b}} {\bar{\mathrm{b}}})$	$g_{{\mathrm{H}} {\mathrm{W}} {\mathrm{W}}} ^{2}g_{{\mathrm{H}} {\mathrm{b}} {\mathrm{b}}} ^{2}/\varGamma _{{\mathrm{H}}}$	$1.9\%$
${\mathrm{H}} {{\nu }}_{\!\mathrm{e}} {\bar{{\nu }}}_{\!\mathrm{e}}$	$\sigma ({\mathrm{H}} {{\nu }}_{\!\mathrm{e}} {\bar{{\nu }}}_{\!\mathrm{e}})\times BR ({\mathrm{H}} \rightarrow {\mathrm{c}} {\bar{{\mathrm{c}}}})$	$g_{{\mathrm{H}} {\mathrm{W}} {\mathrm{W}}} ^{2}g_{{\mathrm{H}} {\mathrm{c}} {\mathrm{c}}} ^{2}/\varGamma _{{\mathrm{H}}}$	$26\%$
${\mathrm{H}} {{\nu }}_{\!\mathrm{e}} {\bar{{\nu }}}_{\!\mathrm{e}}$	$\sigma ({\mathrm{H}} {{\nu }}_{\!\mathrm{e}} {\bar{{\nu }}}_{\!\mathrm{e}})\times BR ({\mathrm{H}} \rightarrow {\mathrm{g}} {\mathrm{g}})$		$10\%$

**Table 31:** Summary of the precisions obtainable for the Higgs observables in the higher-energy CLIC stages for integrated luminosities of $1.5\,\text {ab}^{-1}$ at $\sqrt{s} =1.4\,\text {TeV}$, and $2.0\,\text {ab}^{-1}$ at $\sqrt{s} =3\,\text {TeV}$. In both cases unpolarised beams have been assumed. For $g_{{\mathrm{H}} \mathrm{t} \mathrm{t}}$, the $3\,\text {TeV}$ case has not yet been studied, but is not expected to result in substantial improvement due to the significantly reduced cross section at high energy. Numbers marked with $*$ are extrapolated from $\sqrt{s} =1.4\,\text {TeV}$ to $\sqrt{s} =3\,\text {TeV}$ as explained in the text. For the branching ratios, the measurement precision refers to the expected statistical uncertainty on the product of the relevant cross section and branching ratio; this is equivalent to the expected statistical uncertainty of the product of couplings divided by $\varGamma _{{\mathrm{H}}}$, as indicated in the third column. For the measurements from the ${\mathrm{H}} {\mathrm{H}} {{\nu }}_{\!\mathrm{e}} {\bar{{\nu }}}_{\!\mathrm{e}}$ process, the measurement precisions give the expected statistical uncertainties on the self-coupling parameter $\lambda$

Channel	Measurement	Observable	Statistical precision	
			$1.4\,\text {TeV}$	$3\,\text {TeV}$
			$1.5\,\text {ab}^{-1}$	$2.0\,\text {ab}^{-1}$
${\mathrm{H}} {{\nu }}_{\!\mathrm{e}} {\bar{{\nu }}}_{\!\mathrm{e}}$	${\mathrm{H}} \rightarrow {\mathrm{b}} {\bar{\mathrm{b}}}$ mass distribution	$m_{{\mathrm{H}}}$	$47\,\text {MeV}$	$44\,\text {MeV}$
${\mathrm{H}} {{\nu }}_{\!\mathrm{e}} {\bar{{\nu }}}_{\!\mathrm{e}}$	$\sigma ({\mathrm{H}} {{\nu }}_{\!\mathrm{e}} {\bar{{\nu }}}_{\!\mathrm{e}})\times BR ({\mathrm{H}} \rightarrow {\mathrm{b}} {\bar{\mathrm{b}}})$	$g_{{\mathrm{H}} {\mathrm{W}} {\mathrm{W}}} ^{2}g_{{\mathrm{H}} {\mathrm{b}} {\mathrm{b}}} ^{2}/\varGamma _{{\mathrm{H}}}$	$0.4\%$	$0.3\%$
${\mathrm{H}} {{\nu }}_{\!\mathrm{e}} {\bar{{\nu }}}_{\!\mathrm{e}}$	$\sigma ({\mathrm{H}} {{\nu }}_{\!\mathrm{e}} {\bar{{\nu }}}_{\!\mathrm{e}})\times BR ({\mathrm{H}} \rightarrow {\mathrm{c}} {\bar{{\mathrm{c}}}})$	$g_{{\mathrm{H}} {\mathrm{W}} {\mathrm{W}}} ^{2}g_{{\mathrm{H}} {\mathrm{c}} {\mathrm{c}}} ^{2}/\varGamma _{{\mathrm{H}}}$	$6.1\%$	$6.9\%$
${\mathrm{H}} {{\nu }}_{\!\mathrm{e}} {\bar{{\nu }}}_{\!\mathrm{e}}$	$\sigma ({\mathrm{H}} {{\nu }}_{\!\mathrm{e}} {\bar{{\nu }}}_{\!\mathrm{e}})\times BR ({\mathrm{H}} \rightarrow {\mathrm{g}} {\mathrm{g}})$		$5.0\%$	$4.3\%$
${\mathrm{H}} {{\nu }}_{\!\mathrm{e}} {\bar{{\nu }}}_{\!\mathrm{e}}$	$\sigma ({\mathrm{H}} {{\nu }}_{\!\mathrm{e}} {\bar{{\nu }}}_{\!\mathrm{e}})\times BR ({\mathrm{H}} \rightarrow \uptau ^{+} \uptau ^{-} )$	$g_{{\mathrm{H}} {\mathrm{W}} {\mathrm{W}}} ^{2}g_{{\mathrm{H}} \uptau \uptau } ^{2}/\varGamma _{{\mathrm{H}}}$	$4.2\%$	$4.4\%$
${\mathrm{H}} {{\nu }}_{\!\mathrm{e}} {\bar{{\nu }}}_{\!\mathrm{e}}$	$\sigma ({\mathrm{H}} {{\nu }}_{\!\mathrm{e}} {\bar{{\nu }}}_{\!\mathrm{e}})\times BR ({\mathrm{H}} \rightarrow {{{\upmu }}^{+} {{\upmu }}^{-}})$	$g_{{\mathrm{H}} {\mathrm{W}} {\mathrm{W}}} ^{2}g_{{\mathrm{H}} {\upmu } {\upmu }} ^{2}/\varGamma _{{\mathrm{H}}}$	$38\%$	$25\%$
${\mathrm{H}} {{\nu }}_{\!\mathrm{e}} {\bar{{\nu }}}_{\!\mathrm{e}}$	$\sigma ({\mathrm{H}} {{\nu }}_{\!\mathrm{e}} {\bar{{\nu }}}_{\!\mathrm{e}})\times BR ({\mathrm{H}} \rightarrow \upgamma \upgamma )$		$15\%$	$10\%^*$
${\mathrm{H}} {{\nu }}_{\!\mathrm{e}} {\bar{{\nu }}}_{\!\mathrm{e}}$	$\sigma ({\mathrm{H}} {{\nu }}_{\!\mathrm{e}} {\bar{{\nu }}}_{\!\mathrm{e}})\times BR ({\mathrm{H}} \rightarrow {\mathrm{Z}} \upgamma )$		$42\%$	$30\%^*$
${\mathrm{H}} {{\nu }}_{\!\mathrm{e}} {\bar{{\nu }}}_{\!\mathrm{e}}$	$\sigma ({\mathrm{H}} {{\nu }}_{\!\mathrm{e}} {\bar{{\nu }}}_{\!\mathrm{e}})\times BR ({\mathrm{H}} \rightarrow {\mathrm{W}} {\mathrm{W}} ^*)$	$g_{{\mathrm{H}} {\mathrm{W}} {\mathrm{W}}} ^{4}/\varGamma _{{\mathrm{H}}}$	$1.0\%$	$0.7\%^*$
${\mathrm{H}} {{\nu }}_{\!\mathrm{e}} {\bar{{\nu }}}_{\!\mathrm{e}}$	$\sigma ({\mathrm{H}} {{\nu }}_{\!\mathrm{e}} {\bar{{\nu }}}_{\!\mathrm{e}})\times BR ({\mathrm{H}} \rightarrow {\mathrm{Z}} {\mathrm{Z}} ^*)$	$g_{{\mathrm{H}} {\mathrm{W}} {\mathrm{W}}} ^{2}g_{{\mathrm{H}} {\mathrm{Z}} {\mathrm{Z}}} ^{2}/\varGamma _{{\mathrm{H}}}$	$5.6\%$	$3.9\%^*$
${\mathrm{H}} {\mathrm{e}^{+}}{\mathrm{e}^{-}}$	$\sigma ({\mathrm{H}} {\mathrm{e}^{+}}{\mathrm{e}^{-}})\times BR ({\mathrm{H}} \rightarrow {\mathrm{b}} {\bar{\mathrm{b}}})$	$g_{{\mathrm{H}} {\mathrm{Z}} {\mathrm{Z}}} ^{2}g_{{\mathrm{H}} {\mathrm{b}} {\mathrm{b}}} ^{2}/\varGamma _{{\mathrm{H}}}$	$1.8\%$	$2.3\%^*$
$\mathrm{t} {\bar{\mathrm{t}}} {\mathrm{H}}$	$\sigma (\mathrm{t} {\bar{\mathrm{t}}} {\mathrm{H}})\times BR ({\mathrm{H}} \rightarrow {\mathrm{b}} {\bar{\mathrm{b}}})$	$g_{{\mathrm{H}} \mathrm{t} \mathrm{t}} ^{2}g_{{\mathrm{H}} {\mathrm{b}} {\mathrm{b}}} ^{2}/\varGamma _{{\mathrm{H}}}$	$8\%$	−
${\mathrm{H}} {\mathrm{H}} {{\nu }}_{\!\mathrm{e}} {\bar{{\nu }}}_{\!\mathrm{e}}$	$\sigma ({\mathrm{H}} {\mathrm{H}} {{\nu }}_{\!\mathrm{e}} {\bar{{\nu }}}_{\!\mathrm{e}})$	$\lambda$	$54\%$	$29\%$
${\mathrm{H}} {\mathrm{H}} {{\nu }}_{\!\mathrm{e}} {\bar{{\nu }}}_{\!\mathrm{e}}$	with $-80\%$ $\mathrm{e}^-$ polarisation	$\lambda$	$40\%$	$22\%$

A few of the observables listed in Table 31 were studied only at $\sqrt{s} =1.4\,\text {TeV}$, but not at $\sqrt{s} =3\,\text {TeV}$. In cases where those observables have a significant impact on the combined fits described in this section, the precisions obtained at $\sqrt{s} =1.4\,\text {TeV}$ were extrapolated to $\sqrt{s} =3\,\text {TeV}$. The extrapolation is based on the number of signal events within the detector acceptance at 1.4 and $3\,\text {TeV}$. It is assumed that the background processes scale in the same way with $\sqrt{s}$ as the signal events. However, in fact the signal Higgs bosons are produced in vector boson fusion which increases with increasing $\sqrt{s}$, while several backgrounds are dominated by *s*-channel diagrams which decrease with increasing $\sqrt{s}$.

Since the physical observables ($\sigma$ or $\sigma \times BR$) typically depend on several coupling parameters and on the total width, these parameters are extracted with a combined fit of all measurements. To provide a first indication of the overall impact of the CLIC physics programme, simple fits considering only the statistical uncertainties of the measurements are performed. Two types of fits are used: A model-independent fit making minimal theoretical assumptions, and a model-dependent fit following the strategies used for the interpretation of LHC Higgs results.

Both fits are based on a $\chi ^2$ minimisation using the Minuit package [70]. The measurements which serve as input to the fit, presented in detail in the preceding sections, are either a total cross section $\sigma$ in the case of the measurement of ${\mathrm{e}^{+}}{\mathrm{e}^{-}} \rightarrow {\mathrm{Z}} {\mathrm{H}}$ via the recoil mass technique, or a cross section $\times$ branching ratio $\sigma \times BR$ for specific Higgs production modes and decays. To obtain the expected sensitivity for CLIC it is assumed that for all measurements the value expected in the SM has been measured, so only the statistical uncertainties of each measurement are used in the $\chi ^2$ calculation. In the absence of correlations, the contribution of a single measurement is given by

$$\begin{aligned} \chi ^2_{i} =\frac{(C_i/C_i^{\text {SM}} - 1)^2}{\varDelta F_i^2}, \end{aligned}$$

where $C_i$ is the fitted value of the relevant combination of relevant Higgs couplings (and total width) describing the particular measurement, $C_i^{\text {SM}}$ is the SM expectation, and $\varDelta F_i$ is the statistical uncertainty of the measurement of the considered process. Since this simplified description does not allow the accurate treatment of correlations between measurements, nor the inclusion of correlated theory systematics in the model-dependent fit, the global $\chi ^2$ of the fit is constructed from the covariance matrix of all measurements. It is given by

$$\begin{aligned} \chi ^2 = {\zeta }^T \mathbf{{V}}^{-1} {\zeta }, \end{aligned}$$

where $\mathbf{{V}}$ is the covariance matrix and $\zeta$ is the vector of deviations of fitted values of the relevant combination of Higgs couplings and total width describing the particular measurement deviation from the SM expectation as introduced above, $\zeta _i = C_i/C_i^{\text {SM}} - 1$.

The $C_i$’s depend on the particular measurements and on the type of fit (model-independent or model-dependent), given in detail below. In the absence of systematic uncertainties, the diagonal elements of $\mathbf{{V}}$ are given by the statistical uncertainty of the measurement,

$$\begin{aligned} \mathbf{{V}}_{ii} = \varDelta F_i^2, \end{aligned}$$

while the off-diagonal elements represent the correlations between measurements. In the fit, correlations are taken into account in cases where they are expected to be large. This applies to the measurements of $\sigma \times BR$ for ${\mathrm{H}} \rightarrow {\mathrm{b}} {\bar{\mathrm{b}}} , {\mathrm{c}} {\bar{{\mathrm{c}}}} , {\mathrm{g}} {\mathrm{g}}$ in Higgsstrahlung and ${\mathrm{W}} {\mathrm{W}}$-fusion events at 350 $\text {GeV}$ and in ${\mathrm{W}} {\mathrm{W}}$-fusion events only at 1.4 and 3 $\text {TeV}$, which are extracted in a combined fitting procedure at each energy. These measurements show correlation coefficients with absolute values as large as 0.32.

In signal channels with substantial contaminations from other Higgs decays, penalty terms were added to the $\chi ^2$ to take into account the normalisation of the other channels. These additional uncertainties, which are also of a statistical nature, are derived from the statistical uncertainties of the respective Higgs final state analysis, taking the level of contamination into account. The channels where this results in non-negligible effects are the ${\mathrm{H}} \rightarrow {\mathrm{W}} {\mathrm{W}} ^*$ analyses at all energies, in particular in the all-hadronic decay modes, with corrections to the statistical uncertainties as large as 8% at 350 GeV.

### Model-independent fit

The model-independent fit uses the zero-width approximation to describe the individual measurements in terms of Higgs couplings and the total width, $\varGamma _{{\mathrm{H}}}$. Here, the total cross section of ${\mathrm{e}^{+}}{\mathrm{e}^{-}} \rightarrow {\mathrm{Z}} {\mathrm{H}}$ depends on:

$$\begin{aligned} C_{{\mathrm{Z}} {\mathrm{H}}} = g_{{\mathrm{H}} {\mathrm{Z}} {\mathrm{Z}}}^2, \end{aligned}$$

while for specific final states such as ${\mathrm{e}^{+}}{\mathrm{e}^{-}} \rightarrow {\mathrm{Z}} {\mathrm{H}}$; ${\mathrm{H}} \rightarrow {\mathrm{b}} {\bar{\mathrm{b}}}$ and ${\mathrm{e}^{+}}{\mathrm{e}^{-}} \rightarrow {\mathrm{H}} {{\nu }}_{\!\mathrm{e}} {\bar{{\nu }}}_{\!\mathrm{e}}$; ${\mathrm{H}} \rightarrow {\mathrm{b}} {\bar{\mathrm{b}}}$:

$$\begin{aligned} C_{{{\mathrm{Z}} {\mathrm{H}}},\,{\mathrm{H}} \rightarrow {\mathrm{b}} {\bar{\mathrm{b}}}} = \frac{g_{{\mathrm{H}} {\mathrm{Z}} {\mathrm{Z}}} ^2 g_{{\mathrm{H}} {\mathrm{b}} {\mathrm{b}}} ^2}{\varGamma _{{\mathrm{H}}}} \end{aligned}$$

and:

$$\begin{aligned} C_{{\mathrm{H}} {{\nu }}_{\!\mathrm{e}} {\bar{{\nu }}}_{\!\mathrm{e}} ,\,{\mathrm{H}} \rightarrow {\mathrm{b}} {\bar{\mathrm{b}}}} = \frac{g_{{\mathrm{H}} {\mathrm{W}} {\mathrm{W}}} ^2 g_{{\mathrm{H}} {\mathrm{b}} {\mathrm{b}}} ^2}{\varGamma _{{\mathrm{H}}}}, \end{aligned}$$

respectively.

The fit is performed with 11 free parameters: $g_{{\mathrm{H}} {\mathrm{Z}} {\mathrm{Z}}}$, $g_{{\mathrm{H}} {\mathrm{W}} {\mathrm{W}}}$, $g_{{\mathrm{H}} {\mathrm{b}} {\mathrm{b}}}$, $g_{{\mathrm{H}} {\mathrm{c}} {\mathrm{c}}}$, $g_{{\mathrm{H}} \uptau \uptau }$, $g_{{\mathrm{H}} {\upmu } {\upmu }}$, $g_{{\mathrm{H}} \mathrm{t} \mathrm{t}}$ and $\varGamma _{{\mathrm{H}}}$, as well as the three effective couplings $g^\dagger _\mathrm {{\mathrm{H}} {\mathrm{g}} {\mathrm{g}}}$, $g^\dagger _{{\mathrm{H}} {\upgamma } {\upgamma }}$ and $g^\dagger _{{\mathrm{H}} {\mathrm{Z}} {\upgamma }}$. The latter three parameters are treated in the same way as the physical Higgs couplings in the fit.

**Table 32:** Results of the model-independent fit. Values marked “−” can not be measured with sufficient precision at the given energy. For $g_{{\mathrm{H}} \mathrm{t} \mathrm{t}}$, the $3\,\text {TeV}$ case has not yet been studied, but is not expected to result in substantial improvement due to the significantly reduced cross section at high energy. The three effective couplings $g^\dagger _{{\mathrm{H}} {\mathrm{g}} {\mathrm{g}}}$, $g^\dagger _{{\mathrm{H}} {\upgamma } {\upgamma }}$ and $g^\dagger _{{\mathrm{H}} {\mathrm{Z}} {\upgamma }}$ are also included in the fit. Operation with $-80\%$ electron beam polarisation is assumed above 1 $\text {TeV}$

Parameter	Relative precision		
	$350\,\text {GeV}$	+ $1.4\,\text {TeV}$	+ $3\,\text {TeV}$
	$500\,\text {fb}^{-1}$(%)	+ $1.5\,\text {ab}^{-1}$ (%)	+ $2\,\text {ab}^{-1}$ (%)
$g_{{\mathrm{H}} {\mathrm{Z}} {\mathrm{Z}}}$	0.8	0.8	0.8
$g_{{\mathrm{H}} {\mathrm{W}} {\mathrm{W}}}$	1.4	0.9	0.9
$g_{{\mathrm{H}} {\mathrm{b}} {\mathrm{b}}}$	3.0	1.0	0.9
$g_{{\mathrm{H}} {\mathrm{c}} {\mathrm{c}}}$	6.2	2.3	1.9
$g_{{\mathrm{H}} \uptau \uptau }$	4.3	1.7	1.4
$g_{{\mathrm{H}} {\upmu } {\upmu }}$	−	14.1	7.8
$g_{{\mathrm{H}} \mathrm{t} \mathrm{t}}$	−	4.2	4.2
$g^\dagger _{{\mathrm{H}} {\mathrm{g}} {\mathrm{g}}}$	3.7	1.8	1.4
$g^\dagger _{{\mathrm{H}} {\upgamma } {\upgamma }}$	−	5.7	3.2
$g^\dagger _{{\mathrm{H}} {\mathrm{Z}} {\upgamma }}$	−	15.6	9.1
$\varGamma _{{\mathrm{H}}}$	6.7	3.7	3.5

**Fig. 27:**
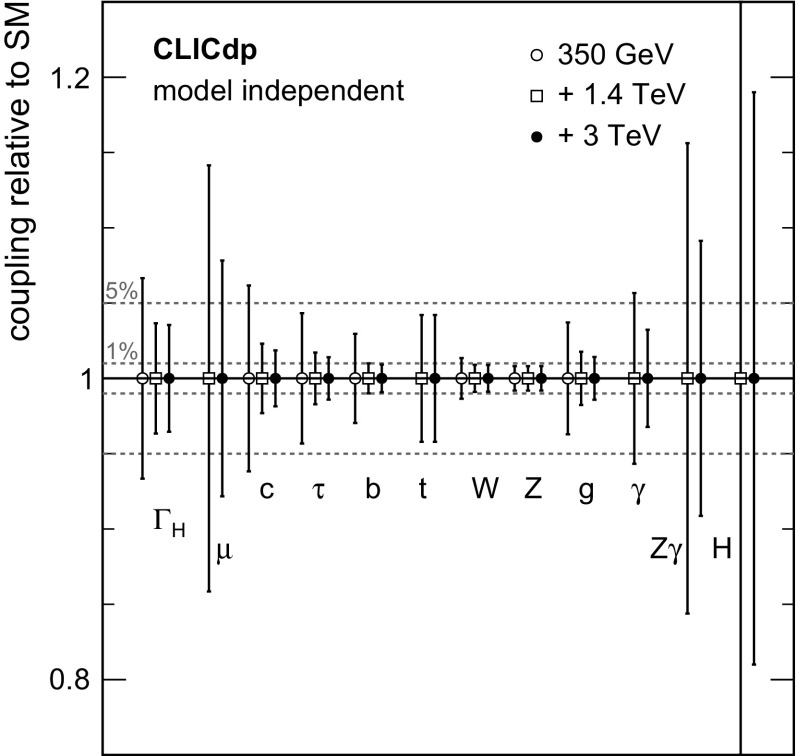
Illustration of the precision of the Higgs couplings of the three-stage CLIC programme determined in a model-independent fit without systematic or theoretical uncertainties. The *dotted lines* show the relative precisions of 1 and 5%

The fit is performed in three stages, taking the statistical uncertainties obtainable from CLIC at the three considered energy stages ($350\,\text {GeV}$, 1.4, $3\,\text {TeV}$) successively into account. Each new stage also includes all measurements of the previous stages. Table 32 summarises the results. They are graphically illustrated in Fig. 27. Since the model-independence of the analysis hinges on the absolute measurement of $\sigma ({\mathrm{Z}} {\mathrm{H}})$ at $350\,\text {GeV}$, which provides the coupling $g_{{\mathrm{H}} {\mathrm{Z}} {\mathrm{Z}}}$, the precision of all other couplings is ultimately limited by this uncertainty.

### Model-dependent fit

For the model-dependent fit, it is assumed that the Higgs decay properties can be described by ten independent parameters $\kappa _{{\mathrm{H}} {\mathrm{Z}} {\mathrm{Z}}}$, $\kappa _{{\mathrm{H}} {\mathrm{W}} {\mathrm{W}}}$, $\kappa _{{\mathrm{H}} {\mathrm{b}} {\mathrm{b}}}$, $\kappa _{{\mathrm{H}} {\mathrm{c}} {\mathrm{c}}}$, $\kappa _{{\mathrm{H}} \uptau \uptau }$, $\kappa _{{\mathrm{H}} {\upmu } {\upmu }}$, $\kappa _{{\mathrm{H}} \mathrm{t} \mathrm{t}}$, $\kappa _{{{\mathrm{H}} {\mathrm{g}} {\mathrm{g}}}}$, $\kappa _{{\mathrm{H}} {\upgamma } {\upgamma }}$ and $\kappa _{{\mathrm{H}} {\mathrm{Z}} {\upgamma }}$. These factors are defined by the ratio of the Higgs partial width divided by the partial width expected in the Standard Model as:

$$\begin{aligned} \kappa _i^2 = \varGamma _i/\varGamma _i^{\text {SM}}. \end{aligned}$$

In this scenario, the total width is given by the sum of the ten partial widths considered, which is equivalent to assuming no non-Standard-Model Higgs decays such as decays into new invisible particles. The ratio of the total width to its SM value is thus given by:

1 $$\begin{aligned} \frac{\varGamma _{{\mathrm{H}},\text {md}}}{\varGamma _{{\mathrm{H}}}^{\text {SM}}} = \sum _i \kappa _i^2 \ BR _i, \end{aligned}$$

where $$BR _i$$ is the SM branching fraction for the respective final state and the subscript “md” stands for “model-dependent”. To obtain these branching fractions, a fixed value for the Higgs mass has to be imposed. For the purpose of this study, $$126\,\text {GeV} $$ is assumed. The branching ratios are taken from the LHC Higgs cross section working group [22]. To exclude effects from numerical rounding errors, the total sum of $$BR $$’s is normalised to unity.

With these definitions, the $C_i$’s in the $\chi ^2$ take the following forms: for the total ${\mathrm{e}^{+}}{\mathrm{e}^{-}} \rightarrow {\mathrm{Z}} {\mathrm{H}}$ cross section:

$$\begin{aligned} C_{{\mathrm{Z}} {\mathrm{H}}} = \kappa _{{\mathrm{H}} {\mathrm{Z}} {\mathrm{Z}}}^2; \end{aligned}$$

while for specific final states such as ${\mathrm{e}^{+}}{\mathrm{e}^{-}} \rightarrow {\mathrm{Z}} {\mathrm{H}}$; ${\mathrm{H}} \rightarrow {\mathrm{b}} {\bar{\mathrm{b}}}$ and ${\mathrm{e}^{+}}{\mathrm{e}^{-}} \rightarrow {\mathrm{H}} {{\nu }}_{\!\mathrm{e}} {\bar{{\nu }}}_{\!\mathrm{e}}$; ${\mathrm{H}} \rightarrow {\mathrm{b}} {\bar{\mathrm{b}}}$:

$$\begin{aligned} C_{{{\mathrm{Z}} {\mathrm{H}}},\,{\mathrm{H}} \rightarrow {\mathrm{b}} {\bar{\mathrm{b}}}} = \frac{\kappa _{{\mathrm{H}} {\mathrm{Z}} {\mathrm{Z}}}^2\kappa _{{\mathrm{H}} {\mathrm{b}} {\mathrm{b}}}^2}{\left( \varGamma _{{\mathrm{H}},\text {md}} / \varGamma _{{\mathrm{H}}}^{\text {SM}}\right) } \end{aligned}$$

and:

$$\begin{aligned} C_{{\mathrm{H}} {{\nu }}_{\!\mathrm{e}} {\bar{{\nu }}}_{\!\mathrm{e}} ,\,{\mathrm{H}} \rightarrow {\mathrm{b}} {\bar{\mathrm{b}}}} = \frac{\kappa _{{\mathrm{H}} {\mathrm{W}} {\mathrm{W}}}^2\kappa _{{\mathrm{H}} {\mathrm{b}} {\mathrm{b}}}^2}{\left( \varGamma _{{\mathrm{H}},\text {md}} / \varGamma _{{\mathrm{H}}}^{\text {SM}}\right) }, \end{aligned}$$

respectively.

Since at the first energy stage of CLIC no significant measurements of the ${\mathrm{H}} \rightarrow {{{\upmu }}^{+} {{\upmu }}^{-}}$, ${\mathrm{H}} \rightarrow {\upgamma } {\upgamma }$ and ${\mathrm{H}} \rightarrow {\mathrm{Z}} {\upgamma }$ decays are possible, the fit is reduced to six free parameters (the coupling to top is also not constrained, but this is without effect on the total width) by setting ${\mathrm{H}} \rightarrow {{{\upmu }}^{+} {{\upmu }}^{-}}$, ${\mathrm{H}} \rightarrow {\upgamma } {\upgamma }$ and ${\mathrm{H}} \rightarrow {\mathrm{Z}} {\upgamma }$ to zero. These branching ratios are much smaller than the derived uncertainty on the total width.

Two versions of the model-dependent fit are performed, one ignoring theoretical uncertainties to illustrate the full potential of the constrained fit, and one taking the present theoretical uncertainties of the branching fractions into account [22]. To avoid systematic biases in the fit results, the uncertainties are symmetrised, preserving the overall size of the uncertainties. Theoretical uncertainties on the production are assumed to be substantially smaller than in the decay, and are ignored in the present study. Depending on the concrete Higgs decay, multiple measurements may enter in the fit, originating from different centre-of-mass energies, different production channels or different signal final states. To account for this, the theoretical uncertainties are treated as fully correlated for each given Higgs decay.

**Table 33:** Results of the model-dependent fit without theoretical uncertainties. Values marked “−” can not be measured with sufficient precision at the given energy. For $g_{{\mathrm{H}} \mathrm{t} \mathrm{t}}$, the $3\,\text {TeV}$ case has not yet been studied, but is not expected to result in substantial improvement due to the significantly reduced cross section at high energy. The uncertainty of the total width is calculated from the fit results following Eq. 1, taking the parameter correlations into account. Operation with $-80\%$ electron beam polarisation is assumed above 1 $\text {TeV}$

Parameter	Relative precision		
	$350\,\text {GeV}$	+ $1.4\,\text {TeV}$	+ $3\,\text {TeV}$
	$500\,\text {fb}^{-1}$(%)	+ $1.5\,\text {ab}^{-1}$ (%)	+ $2\,\text {ab}^{-1}$ (%)
$\kappa _{{\mathrm{H}} {\mathrm{Z}} {\mathrm{Z}}}$	0.6	0.4	0.3
$\kappa _{{\mathrm{H}} {\mathrm{W}} {\mathrm{W}}}$	1.1	0.2	0.1
$\kappa _{{\mathrm{H}} {\mathrm{b}} {\mathrm{b}}}$	1.8	0.4	0.2
$\kappa _{{\mathrm{H}} {\mathrm{c}} {\mathrm{c}}}$	5.8	2.1	1.7
$\kappa _{{\mathrm{H}} \uptau \uptau }$	3.9	1.5	1.1
$\kappa _{{\mathrm{H}} {\upmu } {\upmu }}$	−	14.1	7.8
$\kappa _{{\mathrm{H}} \mathrm{t} \mathrm{t}}$	−	4.1	4.1
$\kappa _{{\mathrm{H}} {\mathrm{g}} {\mathrm{g}}}$	3.0	1.5	1.1
$\kappa _{{\mathrm{H}} {\upgamma } {\upgamma }}$	−	5.6	3.1
$\kappa _{{\mathrm{H}} {\mathrm{Z}} {\upgamma }}$	−	15.6	9.1
$\varGamma _{{\mathrm{H}},\text {md, derived}}$	1.4	0.4	0.3

**Fig. 28:**
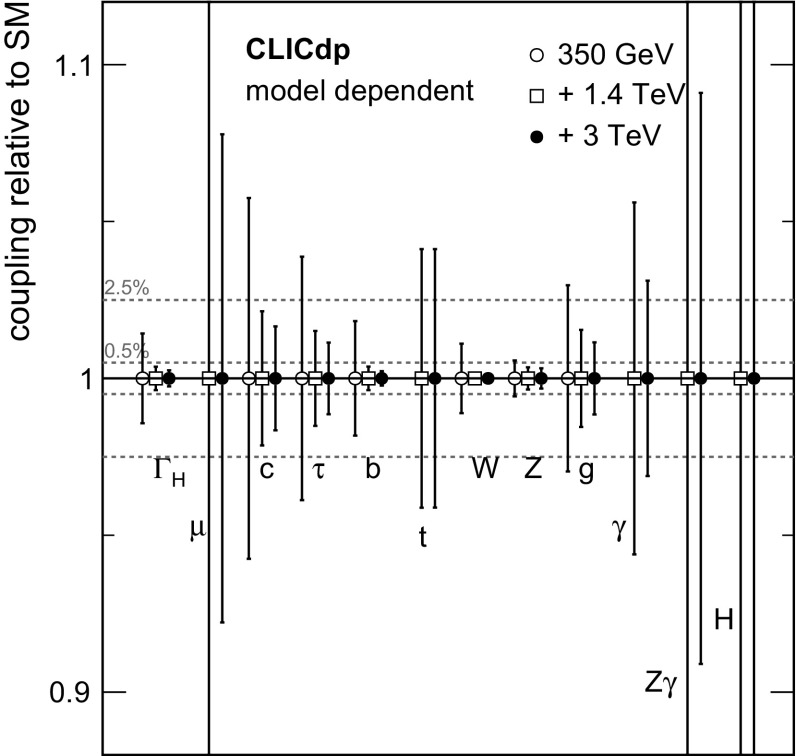
Illustration of the precision of the Higgs couplings of the three-stage CLIC programme determined in a model-dependent fit without systematic or theoretical uncertainties. The *dotted lines* show the relative precisions of 0.5 and 2.5%

As in the model-independent case the fit is performed in three stages, taking the statistical errors of CLIC at the three considered energy stages ($350\,\text {GeV}$, 1.4, $3\,\text {TeV}$) successively into account. Each new stage also includes all measurements of the previous stages. The total width is not a free parameter of the fit. Instead, its uncertainty, based on the assumption given in Eq. 1, is calculated from the fit results, taking the full correlation of all parameters into account. Table 33 summarises the results of the fit without taking theoretical uncertainties into account, and Fig. 28 illustrates the evolution of the precision over the full CLIC programme. Table 34 summarises the results of the model-dependent fit with theoretical uncertainties of the branching fractions.

**Table 34:** Results of the model-dependent fit with the current theoretical uncertainties on the decay branching fractions. Values marked “−” can not be measured with sufficient precision at the given energy. For $g_{{\mathrm{H}} \mathrm{t} \mathrm{t}}$, the $3\,\text {TeV}$ case has not yet been studied, but is not expected to result in substantial improvement due to the significantly reduced cross section at high energy. The uncertainty of the total width is calculated from the fit results following Eq. 1, taking the parameter correlations into account. Operation with $-80\%$ electron beam polarisation is assumed above 1 $\text {TeV}$

Parameter	Relative precision		
	$350\,\text {GeV}$	+ $1.4\,\text {TeV}$	+ $3\,\text {TeV}$
	$500\,\text {fb}^{-1}$(%)	+ $1.5\,\text {ab}^{-1}$ (%)	+ $2\,\text {ab}^{-1}$ (%)
$\kappa _{{\mathrm{H}} {\mathrm{Z}} {\mathrm{Z}}}$	0.6	0.5	0.5
$\kappa _{{\mathrm{H}} {\mathrm{W}} {\mathrm{W}}}$	1.2	0.5	0.5
$\kappa _{{\mathrm{H}} {\mathrm{b}} {\mathrm{b}}}$	2.6	1.5	1.4
$\kappa _{{\mathrm{H}} {\mathrm{c}} {\mathrm{c}}}$	6.3	3.2	2.9
$\kappa _{{\mathrm{H}} \uptau \uptau }$	4.2	2.1	1.8
$\kappa _{{\mathrm{H}} {\upmu } {\upmu }}$	−	14.2	7.9
$\kappa _{{\mathrm{H}} \mathrm{t} \mathrm{t}}$	−	4.2	4.1
$\kappa _{{\mathrm{H}} {\mathrm{g}} {\mathrm{g}}}$	5.1	4.0	3.9
$\kappa _{{\mathrm{H}} {\upgamma } {\upgamma }}$	−	5.9	3.5
$\kappa _{{\mathrm{H}} {\mathrm{Z}} {\upgamma }}$	−	16.0	9.8
$\varGamma _{{\mathrm{H}},\text {md, derived}}$	2.0	1.1	1.1

### Discussion of fit results

**Fig. 29:**
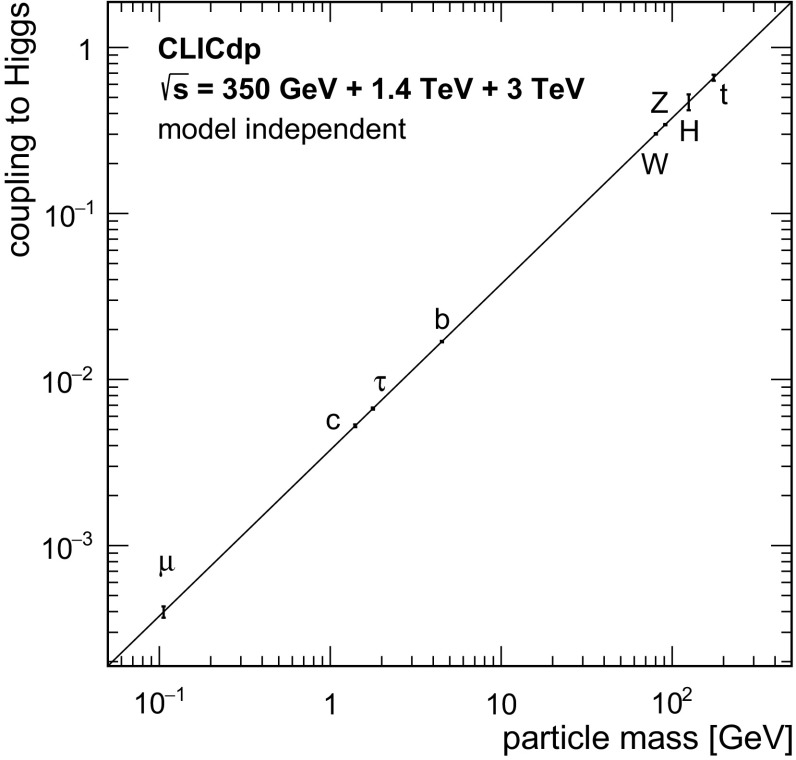
Illustration of the precision of the model-independent Higgs couplings and of the self-coupling as a function of particle mass. The *line* shows the SM prediction that the Higgs coupling of each particle is proportional to its mass

The full Higgs physics programme of CLIC, interpreted with a combined fit of the couplings to fermions and gauge bosons as well as the total width, and combined with the measurement of the self-coupling, will provide a comprehensive picture of the properties of this recently discovered particle. Figure 29 illustrates the expected uncertainties of the various couplings determined in the model-independent fit as well as the self-coupling as a function of the particle mass. Combined with the quasi model-independent measurement of the total width with a precision of $3.5\%$, this illustrates the power of the three-stage CLIC programme. Each of the stages contributes significantly to the total precision, with the first stage at $350\,\text {GeV}$ providing the model-independent “anchor” of the coupling to the ${\mathrm{Z}}$ boson, as well as a first measurement of the total width and coupling measurements to most fermions and bosons. The higher-energy stages add direct measurements of the coupling to top quarks, to muons and photons as well as overall improvements of the branching ratio measurements and with that of the total width and all couplings except the one to the ${\mathrm{Z}}$ already measured in the first stage. They also provide a measurement of the self-coupling of the Higgs boson. In a model-dependent analysis, the improvement with increasing energy is even more significant than in the model-independent fit, since the overall limit of all couplings imposed by the model-independent measurement of the ${\mathrm{Z}} {\mathrm{H}}$ recoil process is removed.

## Summary and conclusions

A detailed study of the Higgs physics reach of CLIC has been presented in the context of CLIC operating in three energy stages, $\sqrt{s} = 350\,\text {GeV}$, 1.4 and $3\,\text {TeV}$. The initial stage of operation, $500\,\text {fb}^{-1}$ at $\sqrt{s} =350\,\text {GeV}$, allows the study of Higgs production from both the ${\mathrm{e}^{+}}{\mathrm{e}^{-}} \rightarrow {\mathrm{Z}} {\mathrm{H}}$ and the ${\mathrm{W}} {\mathrm{W}}$-fusion process. These data yield precise model-independent measurements of the Higgs boson couplings, in particular $\varDelta (g_{{\mathrm{H}} {\mathrm{Z}} {\mathrm{Z}}}) = 0.8\%$, $\varDelta (g_{{\mathrm{H}} {\mathrm{W}} {\mathrm{W}}}) = 1.4\%$ and $\varDelta (g_{{\mathrm{H}} {\mathrm{b}} {\mathrm{b}}}) = 3.0\%$. In addition, the branching ratio to invisible decay modes is constrained to $\varGamma _{\text {invis}}/\varGamma _{{\mathrm{H}}} < 0.01$ at $90\%$ C.L. and the total Higgs width is measured to $\varDelta (\varGamma _{{\mathrm{H}}}) = 6.7\%$. Operation of CLIC at $\sqrt{s} > 1\,\text {TeV}$ provides high-statistics samples of Higgs bosons produced through the ${\mathrm{W}} {\mathrm{W}}$-fusion process and give access to rarer processes such as ${\mathrm{e}^{+}}{\mathrm{e}^{-}} \rightarrow \mathrm{t} {\bar{\mathrm{t}}} {\mathrm{H}}$ and ${\mathrm{e}^{+}}{\mathrm{e}^{-}} \rightarrow {\mathrm{H}} {\mathrm{H}} {{\nu }}_{\!\mathrm{e}} {\bar{{\nu }}}_{\!\mathrm{e}}$. Studies of these rare processes provide measurements of the top Yukawa coupling to $4.2\%$ and the Higgs boson self-coupling to about $20\%$. Furthermore, the full data sample leads to very strong constraints on the Higgs couplings to vector bosons and fermions. In a model-independent treatment, many of the accessible couplings are measured to better than $2\%$, and the model-dependent $\kappa$ parameters are determined with a precision of between 0.1 and $1\%$.

## Footnotes

1 As a result of this paper and other studies, a slightly different staging scenario for CLIC, with a first stage at $\sqrt{s} = 380\,\text {GeV}$ to include precise measurements of top quark properties as a probe for BSM physics, and the next stage at 1.5 TeV, has recently been adopted and will be used for future studies [19].

2 A Higgs boson of $125\,\text {GeV}$ is used in the process ${\mathrm{e}^{+}}{\mathrm{e}^{-}} \rightarrow \mathrm{t} {\bar{\mathrm{t}}} {\mathrm{H}}$.
